# Comparative transcriptomics of 5 high-altitude vertebrates and their low-altitude relatives

**DOI:** 10.1093/gigascience/gix105

**Published:** 2017-11-15

**Authors:** Qianzi Tang, Yiren Gu, Xuming Zhou, Long Jin, Jiuqiang Guan, Rui Liu, Jing Li, Kereng Long, Shilin Tian, Tiandong Che, Silu Hu, Yan Liang, Xuemei Yang, Xuan Tao, Zhijun Zhong, Guosong Wang, Xiaohui Chen, Diyan Li, Jideng Ma, Xun Wang, Miaomiao Mai, An’an Jiang, Xiaolin Luo, Xuebin Lv, Vadim N Gladyshev, Xuewei Li, Mingzhou Li

**Affiliations:** Institute of Animal Genetics and Breeding, College of Animal Science and Technology, Sichuan Agricultural University, Chengdu 611130, China; Animal Breeding and Genetics Key Laboratory of Sichuan Province, Pig Science Institute, Sichuan Animal Science Academy, Chengdu 610066, China; Division of Genetics, Department of Medicine, Brigham and Women's Hospital, Harvard Medical School, Boston, Massachusetts, 02115 USA; Yak Research Institute, Sichuan Academy of Grassland Science, Chengdu 610097, China; Department of Animal Science, Texas A & M University, College Station, Texas, 77843 USA

**Keywords:** high-altitude vertebrates, comparative transcriptomics, gene expression, alternative splicing

## Abstract

**Background:**

Species living at high altitude are subject to strong selective pressures due to inhospitable environments (e.g., hypoxia, low temperature, high solar radiation, and lack of biological production), making these species valuable models for comparative analyses of local adaptation. Studies that have examined high-altitude adaptation have identified a vast array of rapidly evolving genes that characterize the dramatic phenotypic changes in high-altitude animals. However, how high-altitude environment shapes gene expression programs remains largely unknown.

**Findings:**

We generated a total of 910 Gb of high-quality RNA-seq data for 180 samples derived from 6 tissues of 5 agriculturally important high-altitude vertebrates (Tibetan chicken, Tibetan pig, Tibetan sheep, Tibetan goat, and yak) and their cross-fertile relatives living in geographically neighboring low-altitude regions. Of these, ∼75% reads could be aligned to their respective reference genomes, and on average ∼60% of annotated protein coding genes in each organism showed FPKM expression values greater than 0.5. We observed a general concordance in topological relationships between the nucleotide alignments and gene expression–based trees. Tissue and species accounted for markedly more variance than altitude based on either the expression or the alternative splicing patterns. Cross-species clustering analyses showed a tissue-dominated pattern of gene expression and a species-dominated pattern for alternative splicing. We also identified numerous differentially expressed genes that could potentially be involved in phenotypic divergence shaped by high-altitude adaptation.

**Conclusions:**

These data serve as a valuable resource for examining the convergence and divergence of gene expression changes between species as they adapt or acclimatize to high-altitude environments.

## Data Description

### Transcriptome sequencing

**Figure 1:**
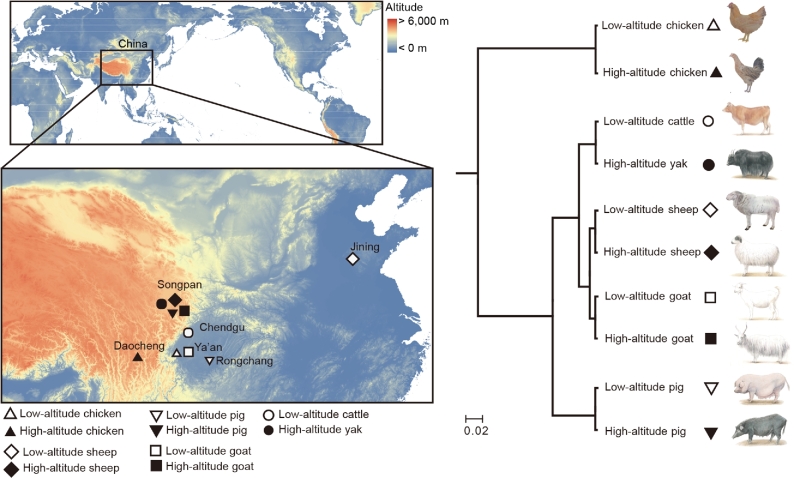
Sampling locations and nucleotide alignment-based tree. (**a**) Geographic locations of the studied animals. (**b**) A neighbor-joining tree constructed based on concatenated coding sequences of single-copy orthologues substituted by SNVs and InDels detected in each animal. We downloaded and extracted the unassembled reads from short-insert (500 bp) libraries of a single yak [1], a Tibetan pig [2], and a Rongchang pig [3] that were used for *de novo* assemblies to roughly ×10 depth coverage. We also randomly selected an individual of the cattle, low- and high-altitude chickens, a goat, and a sheep and sequenced the whole genomes at ∼×10 depth coverage.

Six tissues (heart, kidney, liver, lung, skeletal muscle, and spleen) of 3 unrelated adult females for each of 5 high-altitude vertebrates and their low-altitude relatives were sampled (Fig. 1a and Supplementary Fig. S1). Animals were sacrificed humanely to ameliorate suffering. All animals and samples used in this study were collected according to the guidelines for the care and use of experimental animals established by the Ministry of Agriculture of China. We extracted total RNA, prepared libraries, and sequenced the libraries on Illumina HiSeq 2000 or 2500 platforms. We generated a total of ∼909.6 Gb of high-quality RNA-seq data for 180 samples (∼5.05 Gb per sample) of 30 individuals across 6 tissues (Supplementary Table S1).

### Whole-genome resequencing

To compare the phylogeny derived from gene expression with the phylogenetic relationships of the 5 high-altitude vertebrates and their low-altitude relatives, we constructed the phylogenetic tree based on nucleotide alignments. We extracted the unassembled reads from short-insert (500 bp) libraries of a single yak (NCBI-SRA: SRX103159 to SRX103161, and SRX103175 and SRX103176) [1], a Tibetan pig (NCBI-SRA: SRX219342) [2], and a low-altitude Rongchang pig (NCBI-SRA: SRX1544519) [3] that were used for *de novo* assemblies to roughly ×10 depth coverage. We also randomly selected an individual of the cattle, low- and high-altitude chickens, goats, and sheep and sequenced their whole genomes at ∼×10 depth coverage (NCBI-SRA: SRP096151). Genomic DNA was extracted from the blood tissue of each individual. Sequencing was performed on the Illumina X Ten platform, and a total of 198.64 Gb of paired-end DNA sequence was generated (Supplementary Table S2).

## Data Analysis

### Data filtering

To avoid reads with artificial bias, we removed the following type of reads: (i) reads with ≥10% unidentified nucleotides (N); (ii) reads with >10 nt aligned to the adapter, allowing ≤10% mismatches; (iii) reads with >50% bases having phred quality <5.

### Identification of single-copy orthologous genes

Single-copy orthologous genes across 5 reference genomes, i.e., chicken (Galgal4) [4], pig (Suscrofa 10.2) [5], cattle (UMD3.1) [6], goat (CHIR_1.0) [7], and sheep (Oar_v3.1) [8], were determined using a EnsemblCompara GeneTrees method (Supplementary Fig. S2, Supplementary Methods) [9].

### Construction of phylogenetic tree based on nucleotide alignments

High-quality resequencing data were mapped to their respective reference genomes using BWA software, version 0.7.7 (BWA, RRID:SCR_010910) [10], reads with mapping quality >0 were retained, and potential PCR duplication cases were removed. For each individual, ∼97.01% of reads were mapped to ∼97.40% (at least ×1 depth coverage) or ∼91.86% (at least ×4 depth coverage) of the reference genome assemblies (Supplementary Table S2). Single nucleotide variations (SNVs) and insertion and deletions (InDels) were further detected by following GATK’s best practice, version 3.3–0 (GATK, RRID:SCR_001876) [11]. We substituted SNVs and InDels identified in our study in the coding DNA sequences (CDS) of the respective reference genomes. Single-copy orthologues with the substituted CDS of the 5 vertebrates were applied to Treebest [12] and generated the neighbor-joining tree (Fig. 1b).

#### Analyses of gene expression

High-quality RNA-seq reads were mapped to their respective reference genomes using Tophat version 2.0.11 (TopHat, RRID:SCR_013035) [13]. Cufflinks version 2.2.1 (Cufflinks, RRID:SCR_014597) [14] was applied to quantify gene expression and obtain FPKM expression values. We generated abundance files by applying Cuffquant (part of Cufflinks) to read mapping results. Log_2_-transformed values of (FPKM + 1) for genes with >0.5 FPKM in more than 80% of the samples were used for subsequent analyses.

Pearson's correlations were calculated across 6 samples from low- and high-altitude populations within each group of specific tissues and animals; among pairwise comparisons of 5 animals within each of the 6 tissues; and among pairwise comparisons of 6 tissues within each of the 5 animals. Principal variance component analysis (PVCA) was carried out using R package pvca [15]. Neighbor-joining expression-based trees were generated according to distance matrices composed of pairwise 1-Spearman's correlations implemented in the R package ape [16]. Reproducibility of branching patterns was estimated by bootstrapping genes; i.e., the single-copy orthologues were randomly sampled with replacement 100 times. The fractions of replicate trees that share the branching patterns of the original tree constructed were marked by distinct node colors in the figure.

We generated abundance files by applying Cuffquant (part of Cufflinks) to read mapping results, and further applied abundance files to Cuffdiff (part of Cufflinks) to detect DEGs between population pairs from distinct altitudes within each group of specific tissue and species. Genes with FDR-adjusted *P*-values ≤0.05 were detected as DEGs.

Genes were converted to human orthologues and assessed by the DAVID (DAVID, RRID:SCR_001881) [17] webserver for functional enrichment in gene ontology (GO) terms consisting of molecular function (MF) and biological process (BP), as well as the KEGG (KEGG, RRID:SCR_012773) pathways and InterPro (InterPro, RRID:SCR_006695) databases (Benjamini-adjusted *P* ≤ 0.05).

### Analyses of alternative splicing

Single-copy orthologous exons were identified by finding annotated exons that overlapped with the query exonic region in a multiple alignment of 99 vertebrate genomes including the human genome (hg38) from the UCSC genome browser [18]. Exon groups with multiple overlapping exons in any species were excluded. Each internal exon in every annotated transcript was taken as a “cassette” exon. Each “cassette” alternative splicing (AS) is composed of 3 exons: C1, A, and C2, where A is the alternative exon, C1 the 5’ alternative exon, and C2 the 3’ alternative exon. For each species and read length k, we generated all nonredundant constitutive and alternative junction sequences for the following RNA-seq alignments. The junction sequences were constructed by retrieving k-8 bp from each of the 2 exons making up the junction, and when the exon length is smaller than k-8, the whole sequence of the exon is retrieved. This ensures that there is at least 8-bp overlap between the mapped reads and each of the 2 junction exons.

We then estimated the effective number of uniquely mappable positions of the junctions. We extracted L-k+1 (L being the junction length) k-mers from each junction and mapped such k-mers back to the reference genome, allowing up to 2 mismatches. Those k-mers that failed to align were further mapped to the nonredundant junctions. The number of k-mers that could uniquely align to a junction was counted and deemed the effective number of uniquely mappable positions for the junction.

For each sample, RNA-seq reads were first aligned to the reference genome, allowing up to 2 mismatches, and the unaligned reads were further mapped to the nonredundant junctions. Uniquely mapped reads for each junction were counted and multiplied by the ratio between the maximum number of mappable positions (i.e., k-15) and the effective number of uniquely mappable positions for the junction.

The “percent-spliced in” (PSI) values for each internal exon was defined as PSI = 100 × average (#C1A, #AC2)/(#C1C2 + average(#C1A, #AC2)); here #C1A, #AC2, and #C1C2 are the normalized read counts for the associated junctions. Exons were taken as alternative in a sample if 5 ≤ PSI ≤ 95. We also defined “high-confidence” PSI levels as those that meet the following criteria:

^*^max(min(#C1A, #AC2), #C1C2) ≥ 5 AND min(#C1A, #AC2) + #C1C2 ≥ 10

^*^|log2(#C1A/#AC2)| ≤ 1 OR max(#C1A, #AC2) < #C1C2

For cross-species analyses, we included exons with single-copy orthologues in all species, PSI values in all samples, and confident alternative splicing in at least 1 of the samples.

## Findings

### Data summary

We generated a total of ∼909.6 Gb of high-quality RNA-seq data, of which ∼676.6 Gb (∼74.6%) reads could reliably be aligned to their respective reference genomes (Supplementary Fig. S3 and Table S1). We found that on average 61.2% of annotated protein coding genes in each genome had FPKM expression values greater than 0.5 (Supplementary Fig. S4 and Table S3).

### Concordance in the tree topology based on nucleotide sequence alignments and gene expression data

**Figure 2:**
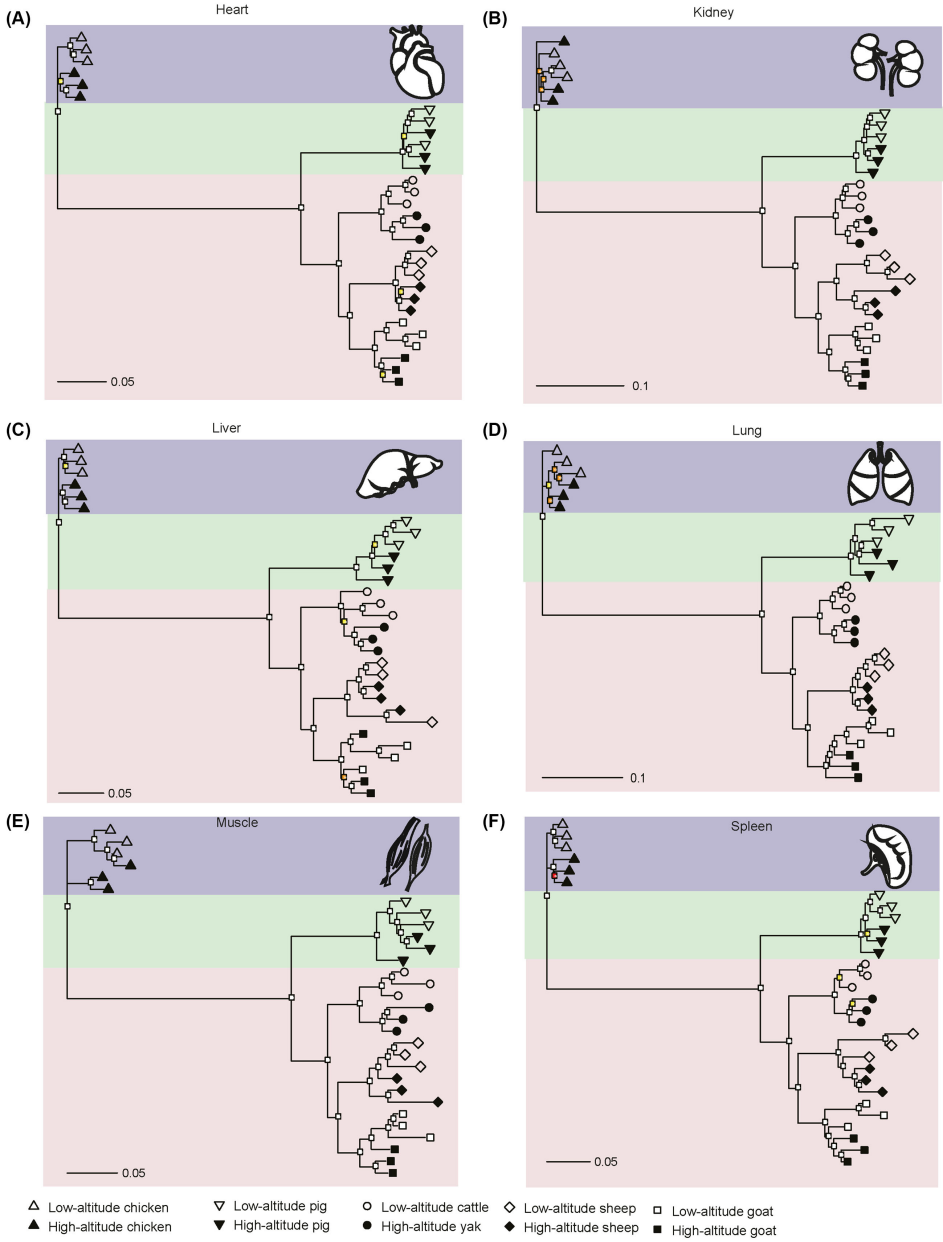
Gene expression phylogenies for 6 tissues across 5 vertebrates. Neighbor-joining expression tree constructed based on 1-Spearman correlation distances in 6 tissues. We performed 100 bootstraps by randomly sampling the single-copy orthologues with replacement. Bootstrap values (fractions of replicate trees that have the branching pattern, as in the shown tree constructed using all the transcribed single-copy orthologues) are indicated by different colors: red color of the node indicates support from less than 50% of bootstraps, while orange, yellow, and white colors indicate support between 50% and 70%, between 70% and 90%, and greater than 90%, respectively.

Nucleotide alignments–based phylogenetic relationships of these high-altitude vertebrates and their low-altitude relatives matched the established morphological species groupings and the known history of population formation (Fig. 1b). The gene expression–based tree of 4746 transcribed single-copy orthologous genes (66.61% of 7125) for each tissue showed a highly consistent topology to the nucleotide sequence alignment-based phylogeny (Fig. 2, Supplementary Methods) [9]: Mammals were mainly divided into omnivore (pig) and ruminant (goat, sheep, and yak/cattle); within the ruminant cluster, the 2 caprinae (goat and sheep) were closer to each other than the bovinae (yak/cattle). This observation lends supports to the idea that gene expression changes evolve together with genetic variation over evolutionary time, resulting in lower expression divergence between more closely species [19].

### Distinctly transcriptomic characteristics between gene expression and alternative splicing

Through comparison of the expression levels of 4746 transcribed single-copy orthologous genes (Supplementary Fig. S2) and the alternative splicing patterns (reflected by PSI values) of 2783 orthologous exons shared by the 5 vertebrates genomes, we observed a tissue-dominated clustering pattern of gene expression, but a species-dominated clustering pattern of alternative splicing [20, 21].

**Figure 3:**
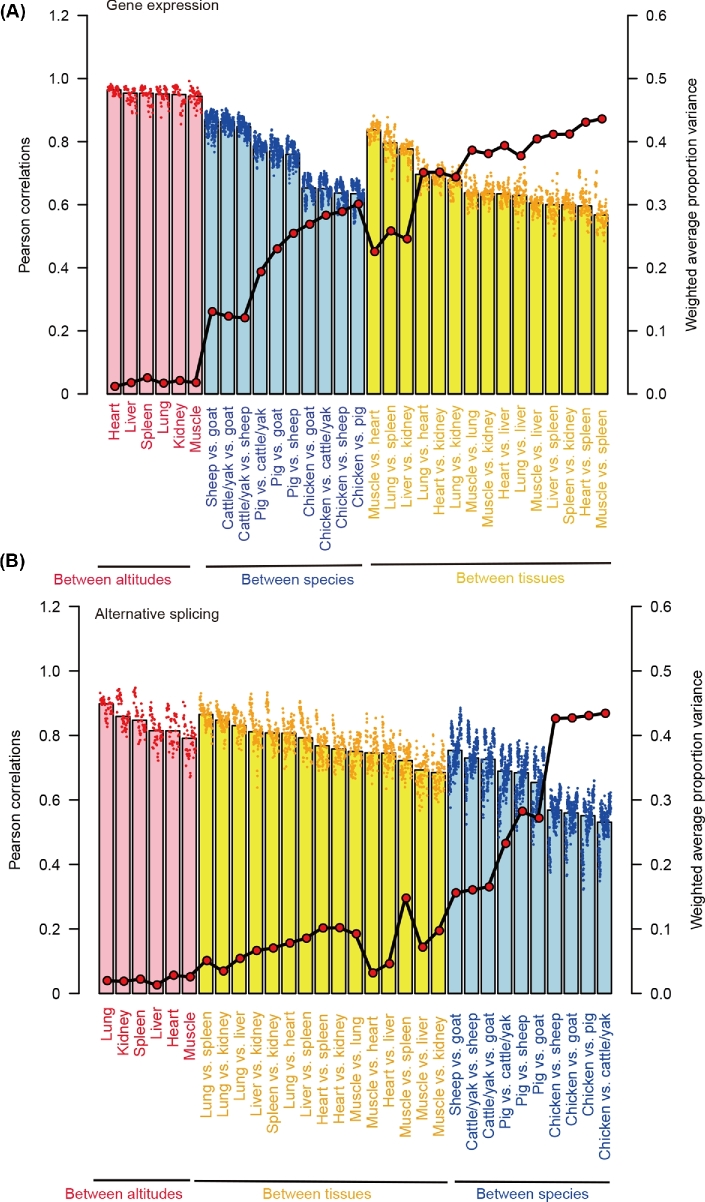
Comparison of variations between altitudes, species, and tissues revealed by (**a**) gene expression and (**b**) alternative splicing pattern. Scatter-point and bar plots represent the pairwise Pearson's correlation between samples. Weighted average proportion variance of the alternative splicing (reflected by PSI values) was determined using the PVCA approach and is depicted as red dots connected by black lines.

For gene expression, there were critical biological differences among tissues (Pearson's *r* = 0.67 and weighted average proportion variance = 0.36), followed by species (Pearson's *r* = 0.75, weighted average proportion variance = 0.22) and local adaptation (Pearson's *r* = 0.95 and weighted average proportion variance = 0.019) (Fig. 3a and Supplementary Fig. S5). By contrast, for alternative splicing, the differences among species (Pearson's *r* = 0.64 and weighted average proportion variance = 0.30) were higher than among tissues (Pearson's *r* = 0.78 and weighted average proportion variance = 0.075), followed by the difference between high- and low-altitude animals (Pearson's *r* = 0.84 and weighted average proportion variance = 0.021) (Fig. 3b and Supplementary Figure S6).

**Figure 4:**
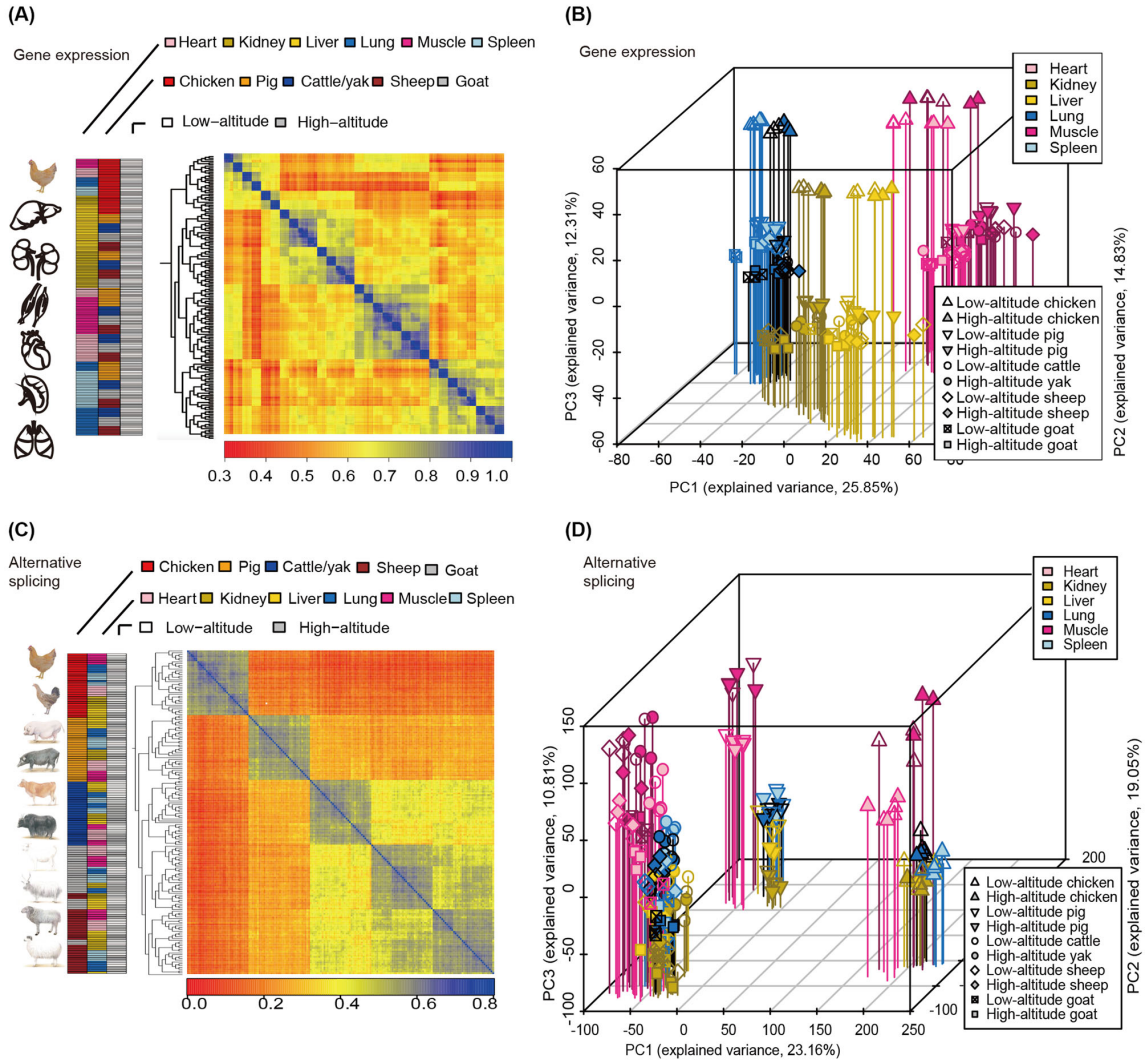
Global pattern of gene expression and alternative splicing pattern. Hierarchical clustering of samples using (**a**) gene expression and (**c**) alternative splicing (reflected by PSI values). Average linkage hierarchical clustering was used with distance between samples measured by Pearson's correlation between the vectors of expression values. (**b, d**) Factorial map of the principal component analysis (PCA) of (b) gene expression levels and (d) the alternative splicing. The proportion of the variance explained by the principal components is indicated in parentheses. The vertical leading lines with different colors from the plotted points dropping to the x/y plane show the separation of points based on the first and second principal components.

Both unsupervised clustering (Fig. 4a and c) and principal components analysis (PCA) (Fig. 4b and d and Supplementary Figs S7 and S8) recapitulated the distinctly transcriptomic characteristics between gene expression and alternative splicing. Tissue-dominated clustering of gene expression indicated that in general tissues possess conserved gene expression signatures and suggested that conserved gene expression differences underlie tissue identity in mammals. On the other hand, greater prominence of species-dominated clustering of alternative splicing suggested that exon splicing is more often affected by species-specific changes in *cis*-regulatory elements and/or *trans*-acting factors than gene expression [20, 21].

Notably, tissue-dominated clustering patterns of gene expression further revealed that the cluster of striated muscle (heart and skeletal muscle) and the cluster of vessel-rich tissues (lung and spleen) were closer to each other than the cluster of metabolic tissues (kidney and liver), followed by the distinct clusters of birds (chicken) and mammals according to the evolutionary distance (Fig. 4a and b). Notably, tissues of birds (chickens) formed a distinct cluster, rather than with their mammalian counterparts, which indicates that divergence in gene expression among these species started to surpass that between different tissues around when birds diverged from mammals (approximately 300 million years ago) (Fig. 4a and b).

### Gene expression plasticity to a high-altitude environment

To exclude the impact of prominence of tissues-dominated clustering of gene expression, so as to comprehensively present transcriptomic differences involved in high-altitude response based on whole annotated genes of their respective genome assembly instead of the single-copy orthologues, we measured the pairwise difference of gene expression between the high-altitude populations and their low-altitude relatives within each tissue for each vertebrate.

We identified ∼1423 DEGs between 30 low- vs high-altitude pairs (177 DEGs in the muscles of chickens to 3853 DEGs in the kidneys of sheep) (Table 1). Notably, among 5 pairs of vertebrates, the highly diverged yak and cattle [1] exhibited the highest number of DEGs (∼2005) across 6 tissues. Among 6 tissues, the highly aerobic kidney [22] exhibited the highest number of DEGs (∼2097) across 5 pairs of vertebrates.

**Table 1:** Number of DEGs between 5 high-altitude vertebrates and their low-altitude relatives for each tissue

Species	Heart No. (%)	Kidney No. (%)	Liver No. (%)	Lung No. (%)	Muscle No. (%)	Spleen No. (%)	Mean No. (%)
Chicken	1283 (8.28)	748 (4.83)	613 (3.96)	1072 (6.92)	177 (1.14)	984 (6.35)	812 (5.25)
Pig	206 (0.95)	532 (2.46)	1199 (5.55)	426 (1.97)	385 (1.78)	994 (4.60)	623 (2.89)
Cattle/yak	1602 (8.02)	1797 (8.99)	869 (4.35)	3092 (15.47)	2403 (12.03)	2268 (11.35)	2005 (10.04)
Sheep	1332 (6.37)	3853 (18.43)	259 (1.24)	1829 (8.75)	1079 (5.16)	2356 (11.27)	1784 (8.54)
Goat	2215 (10.01)	3557 (16.07)	655 (2.96)	1330 (6.01)	2305 (10.42)	1269 (5.73)	1888 (8.53)
Mean	1327 (6.73)	2097 (10.16)	719 (3.61)	1549 (7.82)	1269 (6.11)	1574 (7.86)	

Percentages of the DGEs compared with the total number of annotated protein coding genes in their respective reference genomes are listed in parentheses. There are 15 495, 21 594, 19 981, 22 131, and 20 908 annotated protein coding genes in the reference genomes of the chicken (Galgal4) [4], pig (Suscrofa 10.2) [5], cattle (UMD3.1) [6], goat (CHIR_1.0) [7], and sheep (Oar_v3.1) [8], respectively.

Expectedly, the more closely related vertebrates (Fig. 1) shared more DE genes (Supplementary Figs S9–10 and Additional file 3). Compared with shared DE genes among mammals, especially between the 2 closely related members of Caprinae (goat and sheep), the birds (chickens) exhibited significantly fewer shared DE genes with mammals (Wilcoxon rank sum test, *P* < .0021) (Supplementary Fig. S11). We also identified significantly enriched functional gene categories of DE genes (chi-square test or Fisher's exact test, *P* < 1.03×10^−4^), which were shared among multiple pairwise comparisons (Supplementary Figs S12–13 and Additional file 4) that were potentially related to the dramatic phenotypic changes shaped by high-altitude adaptation, such as response to hypoxia (typically, “oxidation reduction,” “heme binding,” “oxygen binding,” “oxygen transport,” and “oxygen transporter activity”), the cardiovascular system (“angiogenesis” and “positive regulation of angiogenesis”), the efficiency of biomass production in the resource-poor highland (“metabolic pathways,” “cholesterol biosynthetic process,” and “steroid metabolic process”), and immune response (“responses of immune and defense”) (Additional file 2).

## Conclusions

High-altitude adaptive evolution of transcription, and the convergence and divergence of transcriptional alteration across species in response to high-altitude environments, is an important topic of broad interest to the general biology community. Here we provide a comprehensive comparative transcriptome landscape of expression and alternative splicing variation between low- and high-altitude populations across multiple species for distinct tissues. Our data serves a valuable resource for further study on gene regulatory changes to adaptive evolution of complex phenotypes.

## Availability of supporting data

The RNA-seq data for 180 samples was deposited in the NCBI Gene Expression Omnibus (GEO) under accession numbers GSE93855, GSE77020 (note: GSM1617847–GSM1617849 and GSM2042608–GSM2042610 are duplicates and represent the same samples), and GSE66242 (note: 9 goat samples derived from individuals sampled at 2000-meter altitude were not included in this study). The resequencing data for 7 individuals was deposited in the NCBI sequence read archive (SRA) under accession number SRP096151. Supporting data are also available via the *GigaScience* database, *Giga*DB (GigaDB, RRID:SCR_004002) [23].

## Additional files

Supplementary figures and tables are provided as Additional files 1–4.

## Ethics statement

All studies involving animals were conducted according to Regulations for the Administration of Affairs Concerning Experimental Animals (Ministry of Science and Technology, China, revised in June 2004). All experimental procedures and sample collection methods in this study were approved by the Institutional Animal Care and Use Committee of the College of Animal Science and Technology of Sichuan Agricultural University, Sichuan, China, under permit No. DKY-B20121406. Animals were allowed free access to food and water under normal conditions and were humanely sacrificed, as necessary, to ameliorate suffering.

## Competing interests

The authors declare that they have no competing interests.

## Funding

This work was supported by grants from the National High Technology Research and Development Program of China (863 Program; 2013AA102502), the National Natural Science Foundation of China (31402046, 31522055, 31601918, 31530073, 31472081, and 31772576), the Science and Technology Support Program of Sichuan (2016NYZ0042), the Youth Science Fund of Sichuan (2017JQ0011), the China Postdoctoral Science Foundation (2015M572486), China Agriculture Research System (CARS-36), the Program for Innovative Research Team of Sichuan Province (2015TD0012), the Program for Pig Industry Technology System Innovation Team of Sichuan Province (SCCXTD-005), the Project of Sichuan Education Department (15ZA0008, 15ZA0003, 16ZA0025, and 16ZB0037), the National Program for Support of Top-notch Young Professionals, and the Young Scholars of the Yangtze River.

## Author contributions

M.Z.L., Q.Z.T., Y.R.G., and X.W.L. designed and supervised the project. J.Q.G., T.D.C., S.L.H., Y.L., X.M.Y., X.T., Z.J.Z., X.H.C., D.Y.L., X.L.L., and X.B.L. collected the data. L.J., R.L., J.L., K.R.L., S.L.T., G.S.W., J.D.M., X.W., M.M.M., and A.A.J. generated the data. Q.Z.T. and M.Z.L. performed the bioinformatics analyses. Q.Z.T. and M.Z.L. wrote the manuscript. X.M.Z. and V.N.G. revised the manuscript.

## Supplementary Material

GIGA-D-17-00037_Original-Submission.pdf

GIGA-D-17-00037_Revision-1.pdf

GIGA-D-17-00037_Revision-2.pdf

Response-to-Reviewer-Comments_Original-Submission.pdf

Response-to-Reviewer-Comments_Revision-1.pdf

Reviewer-1-Report-(Original-Submission).pdf

Reviewer-1-Report-(Revision-1).pdf

Reviewer-2-Report-(Original-Submission).pdf

Reviewer-2-Report-(Revision-1).pdf

Additional file 1

Additional file 2

Additional file 3

Additional file 4
