# Supplementary material for: Comparative transcriptomics of 5 high-altitude vertebrates and their low-altitude relatives
Source: Gigascience. 2017 Nov 15;6(12):1–9. doi: 10.1093/gigascience/gix105 (PMC5729692; doi:10.1093/gigascience/gix105)
Supplement: GIGA-D-17-00037_Original-Submission.pdf [file gix105_giga-d-17-00037_original-submission.pdf]

# GigaScience

## Comparative transcriptomics of five high-altitude vertebrates and their low-altitude relatives

--Manuscript Draft--

| | |
|---|---|
| **Manuscript Number:** | GIGA-D-17-00037 |
| **Full Title:** | Comparative transcriptomics of five high-altitude vertebrates and their low-altitude relatives |
| **Article Type:** | Data Note |


**Funding Information:**

| | |
|---|---|
| National High Technology Research and Development Program of China (863 Program) (2013AA102502) | Prof. Mingzhou Li |
| National Natural Science Foundation of China (31402046) | Dr Qianzi Tang |
| National Natural Science Foundation of China (31522055) | Prof. Mingzhou Li |
| National Natural Science Foundation of China (31601918) | Dr Jideng Ma |
| National Natural Science Foundation of China (31530073) | Prof Xuewei Li |
| National Natural Science Foundation of China (31472081) | Prof. Mingzhou Li |
| Science & Technology Support Program of Sichuan (2016NYZ0042) | Dr Yiren Gu |
| Youth Science Fund of Sichuan (2017JQ0011) | Dr Yiren Gu |
| China Postdoctoral Science Foundation (2015M572486) | Dr Qianzi Tang |
| China Agriculture Research System (CARS-36) | Dr Yiren Gu |
| Program for Innovative Research Team of Sichuan Province (2015TD0012) | Prof. Mingzhou Li |
| Program for Pig Industry Technology System Innovation Team of Sichuan Province (SCCXTD-005) | Dr Yiren Gu |
| Project of Sichuan Education Department (15ZA0008) | Dr Xun Wang |
| Project of Sichuan Education Department (15ZA0003) | Dr Miaomiao Mai |
| Project of Sichuan Education Department (16ZA0025) | Dr Jideng Ma |
| Project of Sichuan Education Department (16ZB0037) | Dr An'an Jiang |
| National Program for Support of Top-notch Young Professionals | Prof. Mingzhou Li |
| Young Scholars of the Yangtze River | Prof. Mingzhou Li |



**Abstract:**

Background: Species living at high altitude are subject to strong selective pressures due to inhospitable environments (e.g., hypoxia, low temperature, high solar radiation, and lack of biological production), making these species valuable models for comparative analyses of local adaptation. Studies that examined high-altitude adaptation identified a vast array of rapidly evolving genes that characterize the dramatic phenotypic changes in high-altitude animals. However, how high-altitude environment shapes gene expression programs remains largely unknown.



Powered by Editorial Manager® and ProduXion Manager® from Aries Systems Corporation

Findings: We generated a total of 910 Gb high-quality RNA-seq data for 180 samples derived from six tissues of five agriculturally important high-altitude vertebrates (Tibetan chicken, Tibetan pig, Tibetan sheep, Tibetan goat and yak), and their cross-fertile relatives living in geographically neighboring low-altitude regions. Of these, ~75% reads could be aligned to their respective reference genomes, and on average ~70% of annotated protein coding genes in each organism showed FPKM expression values greater than 0.1. We observed a general concordance in topological relationships between the nucleotide alignments and gene expression-based trees. Tissue and species accounted for markedly more variance than altitude based on either the expression or the alternative splicing patterns. Cross-species clustering analyses showed a tissue-dominated pattern of gene expression, and a species-dominated pattern for alternative splicing. We also identified numerous differentially expressed genes were potentially involved in phenotypic divergence shaped by high-altitude adaptation.
Conclusions: This data serves as a valuable resource for examining the convergence and divergence of gene expression changes between species as they adapt or acclimatize to high-altitude environments.


Keywords: high-altitude vertebrates, comparative transcriptomics, gene expression, alternative splicing



| | |
|---|---|
| **Corresponding Author:** | Mingzhou Li, Ph.D.<br>Sichuan Agricultural University<br>Chengdu, Sichuan CHINA |
| **Corresponding Author Secondary Information:** | |
| **Corresponding Author's Institution:** | Sichuan Agricultural University |
| **Corresponding Author's Secondary Institution:** | |
| **First Author:** | Qianzi Tang |
| **First Author Secondary Information:** | |
| **Order of Authors:** | Qianzi Tang |
| | Yiren Gu |
| | Xuming Zhou |
| | Long Jin |
| | Jiuqiang Guan |
| | Rui Liu |
| | Jing Li |
| | Keren Long |
| | Shilin Tian |
| | Tiandong Che |
| | Silu Hu |
| | Yan Liang |
| | Xuemei Yang |
| | Xuan Tao |
| | Zhijun Zhong |
| | Guosong Wang |
| | Xiaohui Chen |
| | Diyan Li |
| | Jideng Ma |
| | |

| | |
|---|---|
| | Xun Wang |
| | Miaomiao Mai |
| | An'an Jiang |
| | Xiaolin Luo |
| | Xuebin Lv |
| | Vadim N. Gladyshev |
| | Xuewei Li |
| | Mingzhou Li, Ph.D. |
| **Order of Authors Secondary Information:** | |
| **Opposed Reviewers:** | Martien A.M. Groenen<br>professor, Wageningen University<br>martien.groenen@wur.nl |
| | Yaping Zhang<br>Professor, Kunming Institute of Zoology Chinese Academy of Sciences<br>zhangyp@mail.kiz.ac.cn |
| **Additional Information:** | |
| **Question** | **Response** |
| Are you submitting this manuscript to a special series or article collection? | No |
| **Experimental design and statistics**<br><br>Full details of the experimental design and statistical methods used should be given in the Methods section, as detailed in our Minimum Standards Reporting Checklist. Information essential to interpreting the data presented should be made available in the figure legends.<br><br>Have you included all the information requested in your manuscript? | Yes |
| **Resources**<br><br>A description of all resources used, including antibodies, cell lines, animals and software tools, with enough information to allow them to be uniquely identified, should be included in the Methods section. Authors are strongly encouraged to cite Research Resource Identifiers (RRIDs) for antibodies, model organisms and tools, where possible.<br><br>Have you included the information requested as detailed in our Minimum Standards Reporting Checklist? | Yes |

| | |
|---|---|
| **Availability of data and materials**<br><br>All datasets and code on which the conclusions of the paper rely must be either included in your submission or deposited in publicly available repositories (where available and ethically appropriate), referencing such data using a unique identifier in the references and in the “Availability of Data and Materials” section of your manuscript.<br><br>Have you have met the above requirement as detailed in our Minimum Standards Reporting Checklist? | Yes |

*Powered by Editorial Manager® and ProduXion Manager® from Aries Systems Corporation*

Manuscript
Click here to download Manuscript Manuscript.docx

# Comparative transcriptomics of five high-altitude vertebrates and their low-altitude relatives

Qianzi Tang[1†], Yiren Gu[2†*], Xuming Zhou[3†], Long Jin[1], Jiuqiang Guan[4], Rui Liu[1], Jing Li[1], Kereng Long[1], Shilin Tian[1], Tiandong Che[1], Silu Hu[1], Yan Liang[2], Xuemei Yang[2], Xuan Tao[2], Zhijun Zhong[2], Guosong Wang[1,5], Xiaohui Chen[2], Diyan Li[1], Jideng Ma[1], Xun Wang[1], Miaomiao Mai[1], An'an Jiang[1], Xiaolin Luo[4], Xuebin Lv[2], Vadim N. Gladyshev[3], Xuewei Li[1*] and Mingzhou Li[1*]

[1] Institute of Animal Genetics and Breeding, College of Animal Science and Technology, Sichuan Agricultural University, Chengdu 611130, China;

[2] Animal Breeding and Genetics Key Laboratory of Sichuan Province, Pig Science Institute, Sichuan Animal Science Academy, Chengdu 610066, China

[3] Division of Genetics, Department of Medicine, Brigham and Women's Hospital, Harvard Medical School, Boston, Massachusetts, 02115 USA;

[4] Yak Research Institute, Sichuan Academy of Grassland Science, Chengdu 610097, China;

[5] Department of Animal Science, Texas A & M University, College Station, Texas, 77843 USA.

[†] These authors contributed equally to this work.

Corresponding authors. E-mail: Mingzhou Li: mingzhou.li@sicau.edu.cn, Xuewei Li: xuewei.li@sicau.edu.cn, Yiren Gu: guyiren1128@163.com.

**Abstract**

**Background:** Species living at high altitude are subject to strong selective pressures due to inhospitable environments (e.g., hypoxia, low temperature, high solar radiation, and lack of biological production), making these species valuable models for comparative analyses of local adaptation. Studies that examined high-altitude adaptation identified a vast array of rapidly evolving genes that characterize the dramatic phenotypic changes in high-altitude animals. However, how high-altitude environment shapes gene expression programs remains largely unknown.

**Findings:** We generated a total of 910 Gb high-quality RNA-seq data for 180 samples derived from six tissues of five agriculturally important high-altitude vertebrates (Tibetan chicken, Tibetan pig, Tibetan sheep, Tibetan goat and yak), and their cross-fertile relatives living in geographically neighboring low-altitude regions. Of these, ~75% reads could be aligned to their respective reference genomes, and on average ~70% of annotated protein coding genes in each organism showed FPKM expression values greater than 0.1. We observed a general concordance in topological relationships between the nucleotide alignments and gene expression-based trees. Tissue and species accounted for markedly more variance than altitude based on either the expression or the alternative splicing patterns. Cross-species clustering analyses showed a tissue-dominated pattern of gene expression, and a species-dominated pattern for alternative splicing. We also identified numerous differentially expressed genes were potentially involved in phenotypic divergence shaped by high-altitude adaptation.

**Conclusions:** This data serves as a valuable resource for examining the

convergence and divergence of gene expression changes between species as they adapt or acclimatize to high-altitude environments.

**Keywords:** high-altitude vertebrates, comparative transcriptomics, gene expression, alternative splicing

## Data description

### *Transcriptome sequencing*

Six tissues (heart, kidney, liver, lung, skeletal muscle and spleen) of three unrelated adult females for each of five high-altitude vertebrates and their low-altitude relatives were sampled **(Fig. 1a** and **Supplementary Fig. S1)**. Animals were sacrificed humanely to ameliorate suffering. All animals and samples used in this study were collected according to the guidelines for the care and use of experimental animals established by the Ministry of Agriculture of China. We extracted total RNA, prepared libraries and sequenced the libraries on Illumina HiSeq 2000 or 2500 platforms. We generated a total of ~909.6 Gb high-quality RNA-seq data for 180 samples (~5.05 Gb per sample) of 30 individuals across 6 tissues **(Supplementary Table S1)**.

### *Whole-genome re-sequencing*

To compare the phylogeny derived from gene expression with the phylogenetic relationships of the five high-altitude vertebrates and their low-altitude relatives, we constructed the phylogenetic tree based on nucleotide alignments. We extracted the unassembled reads from short-insert (500 bp) libraries of a single yak [1] (NCBI-SRA: SRX103159 to SRX103161, and SRX103175 and SRX103176), a Tibetan pig [2] (NCBI-SRA: SRX219342) and a low-altitude Rongchang pig (NCBI-SRA: SRX1544519) [3] that were used for *de novo*

assemblies to roughly 10 × depth coverage. We also randomly selected an individual of the cattle, low- and high-altitude chicken, goat and sheep, and sequenced their whole genomes at ~10 × depth coverage (NCBI-SRA: SRP096151). Genomic DNA was extracted from blood tissue of each individual. Sequencing was performed on the Illumina X Ten platform, and a total of 198.64 Gb of paired-end DNA sequence was generated **(Supplementary Table S2)**.

## Data analysis

***Data filtering***

To avoid reads with artificial bias, we removed the following type of reads: (a) Reads with ≥ 10% unidentified nucleotides (N); (b) Reads with > 10 nt aligned to the adapter, allowing ≤ 10% mismatches; (c) Reads with > 50% bases having phred quality < 5.

***Identification of single-copy orthologous genes***

Single-copy orthologous genes across five reference genomes, i.e. chicken (Galgal4) [4], pig (Suscrofa 10.2) [5], cattle (UMD3.1) [6], goat (CHIR_1.0) [7] and sheep (Oar_v3.1) [8] were determined using a EnsemblCompara GeneTrees method [9] **(Supplementary Fig. S2).**

***Construction of phylogenetic tree based on nucleotide alignments***

High-quality re-sequencing data were mapped to their respective reference genomes using BWA software (version 0.7.7) [10], reads with mapping quality > 0 were retained and potential PCR duplication cases were removed. For each individual, ~97.01% of reads were mapped to ~97.40% (at least 1 × depth coverage) or ~91.86% (at least 4 × depth coverage) of the reference genome

assemblies **(Supplementary Table S2)**. Single nucleotide variations (SNVs) and insertion and deletions (InDels) were further detected by following GATK's best practice (version 3.3-0) [11]. We substituted SNVs and InDels identified in our study in the coding DNA sequences (CDS) of the respective reference genomes. Single copy orthologues with substituted CDS of the five vertebrates were applied to Treebest [12] and generating the neighbor-joining tree **(Fig. 1b)**.

***Analyses of gene expression***

High-quality RNA-seq reads were mapped to their respective reference genomes using Tophat (version 2.0.11) [13]. Cufflinks (version 2.2.1) [14] was applied to quantify gene expression and obtain FPKM expression values. We generated abundance files by applying Cuffquant (part of Cufflinks) to read mapping results. $Log_2$-transformed values of (FPKM + 1) were used in subsequent analyses.

Pearson's correlations were calculated across six samples from low- and high-altitudes populations within each group of specific tissue and animals; among pairwise comparisons of five animals within each of the six tissues; and among pairwise comparisons of six tissues within each of the five animals. Principal Variance Component Analysis (PVCA) was carried out using R package pvca [15]. Neighbor-joining expression-based trees were generated according to distance matrices composed of pairwise (1-Spearman's correlations) implemented in R package ape [16]. Reproducibility of branching patterns was estimated by bootstrapping genes, that is, the single copy orthologues were randomly sampled with replacement 100 times. The fractions of replicate trees that share the branching patterns of the original tree constructed were marked by distinct node colors in the figure.

We generated abundance files by applying Cuffquant (part of Cufflinks) to read mapping results, and further applied abundance files to Cuffdiff (part of Cufflinks) to detect DEGs between population pairs from distinct altitudes within each group of specific tissue and species. Genes with FDR-adjusted p-values ≤ 0.05 were detected as DEGs.

Genes were converted to human orthologs, and assessed by DAVID [17] webserver for functional enrichment in GO (Gene Ontology) terms consisting of molecular function (MF) and biological process (BP) as well as the KEGG pathways and InterPro databases (Benjamini adjusted p-values ≤ 0.05).

***Analyses of alternative splicing***

Single-copy orthologous exons were identified by finding annotated exons that overlapped with the query exonic region in a multiple alignment of 99 vertebrate genomes including human genome (hg38) from the UCSC genome browser [18]. Exon groups with multiple overlapping exons in any species were excluded. Each internal exon in every annotated transcript was taken as an “cassette” exon. Each “cassett” alternative splicing (AS) is composed of three exons: C1, A and C2, where A is alternative exon, C1 the 5’ alternative exon, C2 the 3’ alternative exon. For each species and read length k, we generated all non-redundant constitutive and alternative junction sequences for the following RNA-seq alignments. The junction sequences were constructed by retrieving k-8 bp from each of the two exons making up the junction, and when the exon length is smaller than k-8, the whole sequence of the exon is retrieved. This ensures that there is at least 8 bp overlap between the mapped reads and each of the two junction exons.

We then estimated the effective number of uniquely mappable positions of

the junctions. We extracted L-k+1 (L being the junction length) k-mers from each junction and mapped such k-mers back to the reference genome allowing up to two mismatches. Those k-mers that failed to align were further mapped to the non-redundant junctions. The number of k-mers that could uniquely align to a junction was counted and deemed as the effective number of uniquely mappable positions for the junction.

For each sample, RNA-seq reads were first aligned to the reference genome allowing up to two mismatches, and the unaligned reads were further mapped to the non-redundant junctions. Uniquely mapped reads for each junction were counted, and multiplied by the ratio between the maximum number of mappable positions (i.e. k-15) to the effective number of uniquely mappable positions for the junction.

The “percent-spliced in” (PSI) values for each internal exon was defined as $PSI = 100 \times average(\#C1A, \#AC2) / (\#C1C2 + average(\#C1A, \#AC2))$, here #C1A, #AC2 and #C1C2 are the normalized read counts for the associated junctions. Exons were taken as alternative in a sample if $5 \leq PSI \leq 95$. We also defined “high-confidence” PSI levels as those that meet the following criteria:

*$max(min(\#C1A, \#AC2), \#C1C2) \geq 5$ AND $min(\#C1A, \#AC2) + \#C1C2 \geq 10$

*$|log2(\#C1A / \#AC2)| \leq 1$ OR $max(\#C1A, \#AC2) < \#C1C2$

For cross-species analyses, we included exons with single-copy orthologues in all species, PSI values in all samples, and confidently alternative spliced in at least one of the samples.

# Findings

## *Data summary*

We generated a total of ~909.6 Gb high-quality RNA-seq data, of which ~676.6 Gb (~74.6%) reads could reliably aligned to their respective reference genomes **(Supplementary Fig. S3** and **Table S1)**. We found that on average 69.7% annotated protein coding genes in each genome had FPKM expression values greater than 0.1 **(Supplementary Fig. S4** and **Table S3)**.

## *Concordance in the tree topology based on nucleotide sequence alignments and gene expression data*

Nucleotide alignments-based phylogenetic relationships of these high-altitude vertebrates and their low-altitude relatives matched the established morphological species groupings and the known history of population formation **(Fig. 1b)**. The gene expression-based tree based 7,125 high-confidence single-copy orthologous genes for each tissue showed a highly consistent topology to the nucleotide sequence alignment-based phylogeny **(Fig. 2)**: mammals were mainly divided into omnivore (pig) and ruminant (goat, sheep and yak/cattle); within the ruminant cluster, the two caprinae (goat and sheep) were closer to each other than the bovinae (yak/cattle). This observation lends supports the idea that gene expression changes evolve together with genetic variation over evolutionary time, resulting in lower expression divergence between more closely species [19].

## *Distinctly transcriptomic characteristics between gene expression and alternative splicing*

Through comparison of expression levels of 7,125 high-confidence single-copy orthologous genes **(Supplementary Fig. S2)** and alternative splicing

patterns (reflected by PSI values) of 2,783 orthologous exons shared by the five vertebrates genomes, we observed a tissue-dominated clustering pattern of gene expression, but a species-dominated clustering pattern of alternative splicing [20, 21].

For gene expression, there were critical biological differences among tissues (Pearson's $r$ = 0.71 and weighted average proportion variance = 0.42), followed by species (Pearson's $r$ = 0.84, weighted average proportion variance = 0.16) and local adaptation (Pearson's $r$ = 0.97 and weighted average proportion variance = 0.019) **(Fig. 3a** and **Supplementary Fig. S5)**. By contrast, for alternative splicing, the differences among species (Pearson's $r$ = 0.64 and weighted average proportion variance = 0.30) were higher than among tissues (Pearson's $r$ = 0.78 and weighted average proportion variance = 0.075), followed by between high- and low-altitude animals (Pearson's $r$ = 0.84 and weighted average proportion variance = 0.021) **(Fig. 3b** and **Supplementary Figure S6)**.

Unsupervised clustering **(Figs. 4a and 4c)** and principal components analysis (PCA) **(Figs. 4b and 4d** and **Supplementary Figs. S7 and S8)** both recapitulated the distinctly transcriptomic characteristics between gene expression and alternative splicing. Tissue-dominated clustering of gene expression indicated that in general tissues possess conserved gene expression signatures and suggested that conserved gene expression differences underlie tissue identity in mammals. On the other hand, greater prominence of species-dominated clustering of alternative splicing suggested that exon splicing is more often affected by species-specific changes in *cis*-regulatory elements and/or *trans*-acting factors than gene expression [20, 21].

Notably, tissue-dominated clustering patterns of gene expression further revealed that the cluster of striated muscle (heart and skeletal muscle) and the cluster of vessel-rich tissues (lung and spleen) were closer to each other than the cluster of metabolic tissues (kidney and liver), followed by the distinct clusters of bird (chicken) and mammals according to the evolutionary distance **(Figs. 4a and 4b)**. The exceptions to tissue dominance were that chicken heart, lung and liver clustered with chicken skeletal muscle, spleen and kidney, respectively, rather than with their mammalian counterparts, which implied that divergence in gene expression among these species started to surpass those between different tissues at about the time when birds split from mammals (~300 million years) **(Figs. 4a and 4b)**.

### *Gene expression plasticity to a high-altitude environment*

To exclude the impact of prominence of tissues-dominated clustering of gene expression, so as to comprehensively present transcriptomic differences involved in high-altitude response based on whole annotated genes of their respective genome assembly instead of the single-copy orthologous, we measured the pairwise difference of gene expression between the high-altitude populations and their low-altitude relatives within each tissue for each vertebrate.

We identified ~1,512 DEGs between 30 low- versus high-altitude pairs (225 DEGs in liver of pigs to 4,014 DEGs in kidney of sheep) (**Table 1**). Notably, among five pairs of vertebrate, the highly-diverged yak and cattle [1] exhibited the highest number of DEG (~2,242) across six tissues. Among six tissues, the highly aerobic kidney [22] exhibited the highest number of DEGs (~2,103) across five pairs of vertebrates. As expected, respectable significantly enriched

functional gene categories by DGEs, which shared in multiple pair-wise comparisons, were potentially related to the dramatic phenotypic changes shaped by high-altitude adaptation, such as response to hypoxia (typically, ‘oxidation reduction’, ‘heme binding’, ‘oxygen binding’ , ‘response to oxygen levels’ and ‘response to hypoxia’), cardiovascular system (‘blood vessel development’, ‘blood vessel morphogenesis’, ‘blood circulation’ and ‘development of lung and heart’), the efficiency of biomass production in the resource-poor highland (processes of ‘steroid biosynthesis’ and ‘fatty acid metabolism’) as well as immune response (‘‘responses of immune and defense’) (**Additional file 2**).

## Conclusions

High-altitude adaptive evolution of transcription, and the convergence and divergence of transcriptional alteration across species in response to high-altitude environments, is an important topic of broad interest to the general biology community. Here we provide a comprehensive comparative transcriptome landscape of expression and alternative splicing variation between low- and high-altitude populations across multiple species for distinct tissues. Our data serves a valuable resource for further study on gene regulatory changes to adaptive evolution of complex phenotypes.

**Availability of supporting data**

The RNA-seq data for 180 samples was deposited in the NCBI Gene Expression Omnibus (GEO) under accession numbers GSE93855, GSE77020 and GSE66242. The re-sequencing data for 7 individuals was deposited in the

NCBI-sequence read archive (SRA) under accession number SRP096151.

All supplementary figures and tables are provided in Additional file.

**Reviewer links:**

GSE93855:

https://www.ncbi.nlm.nih.gov/geo/query/acc.cgi?token=irqtigkgvtatnqt&acc=GSE93855

GSE77020:

https://www.ncbi.nlm.nih.gov/geo/query/acc.cgi?token=kpolsqsothybrcv&acc=GSE77020 (GSM1617847-GSM1617849 and GSM2042608-GSM2042610 are duplicates and represent the same samples)

GSE66242:

https://www.ncbi.nlm.nih.gov/geo/query/acc.cgi?token=absxuuywtfyhncx&acc=GSE66242 (9 goat samples derived from individuals sampled at 2000m altitude were not included in this study)

**Ethics statement**

All studies involving animals were conducted according to Regulations for the Administration of Affairs Concerning Experimental Animals (Ministry of Science and Technology, China, revised in June 2004). All experimental procedures and sample collection methods in this study were approved by the Institutional Animal Care and Use Committee of the College of Animal Science and Technology of Sichuan Agricultural University, Sichuan, China, under permit No. DKY-B20121406. Animals were allowed free access to food and water under

normal conditions, and were humanely sacrificed as necessary, to ameliorate suffering.

**Consent for publication**

Not applicable.

**Competing interests**

The authors declare that they have no competing interests.

**Funding**

This work was supported by grants from the National High Technology Research and Development Program of China (863 Program) (2013AA102502), the National Natural Science Foundation of China (31402046, 31522055, 31601918, 31530073 and 31472081), the Science & Technology Support Program of Sichuan (2016NYZ0042), the Youth Science Fund of Sichuan (2017JQ0011), the China Postdoctoral Science Foundation (2015M572486), China Agriculture Research System (CARS-36), the Program for Innovative Research Team of Sichuan Province (2015TD0012), the Program for Pig Industry Technology System Innovation Team of Sichuan Province (SCCXTD-005), the Project of Sichuan Education Department (15ZA0008, 15ZA0003, 16ZA0025 and 16ZB0037), the National Program for Support of Top-notch Young Professionals and the Young Scholars of the Yangtze River.

**Authors' contributions**

MZ.L., QZ.T., YR.G. and XW.L. designed and supervised the project. JQ.G., TD.C., SL.H., Y.L., XM.Y., X.T., ZJ.Z., XH.C., DY.L., XL.L. and XB.L. collected the data, L.J., R.L., J.L., KR.L., SL.T., GS.W., JD.M., X.W., MM.M. and AA.J. generated the data. QZ.T. and MZ.L. performed the bioinformatics analyses.

QZ.T. and MZ.L. wrote the manuscript. XM.Z. and VN.G. revised the manuscript.

## References


1. Qiu Q, Zhang G, Ma T, Qian W, Wang J, Ye Z, et al. The yak genome and adaptation to life at high altitude. Nat Genet. 2012;44 8:946-9. doi:10.1038/ng.2343.
2. Li M, Tian S, Jin L, Zhou G, Li Y, Zhang Y, et al. Genomic analyses identify distinct patterns of selection in domesticated pigs and Tibetan wild boars. Nature genetics. 2013;45 12:1431-8. doi:10.1038/ng.2811.
3. Li M, Chen L, Tian S, Lin Y, Tang Q, Zhou X, et al. Comprehensive variation discovery and recovery of missing sequence in the pig genome using multiple *de novo* assemblies. Genome Res. 2016; doi:10.1101/gr.207456.116.
4. International Chicken Genome Sequencing Consortium. Sequence and comparative analysis of the chicken genome provide unique perspectives on vertebrate evolution. Nature. 2004;432 7018:695-716. doi:10.1038/nature03154.
5. Groenen MA, Archibald AL, Uenishi H, Tuggle CK, Takeuchi Y, Rothschild MF, et al. Analyses of pig genomes provide insight into porcine demography and evolution. Nature. 2012;491 7424:393-8. doi:10.1038/nature11622.
6. Bovine Genome S, Analysis C, Elsik CG, Tellam RL, Worley KC, Gibbs RA, et al. The genome sequence of taurine cattle: a window to ruminant biology and evolution. Science. 2009;324 5926:522-8. doi:10.1126/science.1169588.

7. Dong Y, Xie M, Jiang Y, Xiao N, Du X, Zhang W, et al. Sequencing and automated whole-genome optical mapping of the genome of a domestic goat *(Capra hircus)*. Nat Biotechnol. 2013;31 2:135-41. doi:10.1038/nbt.2478.

8. Jiang Y, Xie M, Chen W, Talbot R, Maddox JF, Faraut T, et al. The sheep genome illuminates biology of the rumen and lipid metabolism. Science. 2014;344 6188:1168-73. doi:10.1126/science.1252806.

9. Vilella AJ, Severin J, Ureta-Vidal A, Heng L, Durbin R and Birney E. EnsemblCompara GeneTrees: Complete, duplication-aware phylogenetic trees in vertebrates. Genome Res. 2009;19 2:327-35. doi:10.1101/gr.073585.107.

10. Li H and Durbin R. Fast and accurate long-read alignment with Burrows-Wheeler transform. Bioinformatics. 2010;26 5:589-95. doi:10.1093/bioinformatics/btp698.

11. McKenna A, Hanna M, Banks E, Sivachenko A, Cibulskis K, Kernytsky A, et al. The Genome Analysis Toolkit: a MapReduce framework for analyzing next-generation DNA sequencing data. Genome Res. 2010;20 9:1297-303. doi:10.1101/gr.107524.110.

12. Li H, Coghlan A, Ruan J, Coin LJ, Heriche JK, Osmotherly L, et al. TreeFam: a curated database of phylogenetic trees of animal gene families. Nucleic Acids Res. 2006;34 Database issue:D572-80. doi:10.1093/nar/gkj118.

13. Trapnell C, Pachter L and Salzberg SL. TopHat: discovering splice junctions with RNA-Seq. Bioinformatics. 2009;25 9:1105-11. doi:10.1093/bioinformatics/btp120.

14. Trapnell C, Williams BA, Pertea G, Mortazavi A, Kwan G, van Baren MJ, et al.

Transcript assembly and quantification by RNA-Seq reveals unannotated transcripts and isoform switching during cell differentiation. Nat Biotechnol. 2010;28 5:511-5. doi:10.1038/nbt.1621.

15. The pvca R package. https://bioconductor.org/packages/release/bioc/html/pvca.html. Accessed Feb 16 2017.

16. Paradis E, Claude J and Strimmer K. APE: Analyses of Phylogenetics and Evolution in R language. Bioinformatics. 2004;20 2:289-90.

17. Dennis G, Jr., Sherman BT, Hosack DA, Yang J, Gao W, Lane HC, et al. DAVID: Database for Annotation, Visualization, and Integrated Discovery. Genome Biol. 2003;4 5:P3.

18. Kent WJ, Sugnet CW, Furey TS, Roskin KM, Pringle TH, Zahler AM, et al. The human genome browser at UCSC. Genome Res. 2002;12 6:996-1006. doi:10.1101/gr.229102. Article published online before print in May 2002.

19. Brawand D, Soumillon M, Necsulea A, Julien P, Csardi G, Harrigan P, et al. The evolution of gene expression levels in mammalian organs. Nature. 2011;478 7369:343-8. doi:10.1038/nature10532.

20. Merkin J, Russell C, Chen P and Burge CB. Evolutionary dynamics of gene and isoform regulation in Mammalian tissues. Science. 2012;338 6114:1593-9. doi:10.1126/science.1228186.

21. Barbosa-Morais NL, Irimia M, Pan Q, Xiong HY, Gueroussov S, Lee LJ, et al. The evolutionary landscape of alternative splicing in vertebrate species. Science. 2012;338 6114:1587-93. doi:10.1126/science.1230612.

22. Mele M, Ferreira PG, Reverter F, DeLuca DS, Monlong J, Sammeth M, et al. Human genomics. The human transcriptome across tissues and individuals. Science. 2015;348 6235:660-5. doi:10.1126/science.aaa0355.

## Figures 1-4

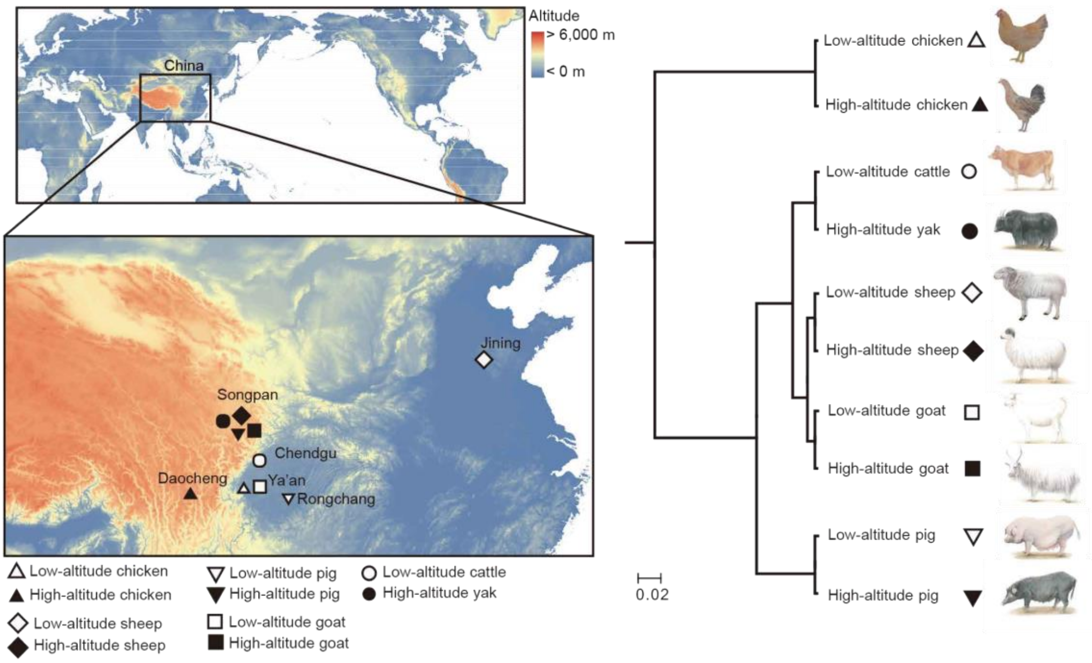


**Figure 1. Sampling locations and nucleotide alignment-based tree.**

**(a)** Geographic locations of the studied animals.

**(b)** A neighbour-joining tree constructed based on concatenated coding sequences of single-copy orthologues substituted by SNVs and InDels detected in each animal.

We downloaded and extracted the unassembled reads from short-insert (500 bp) libraries of a single yak [1], a Tibetan pig [2] and a Rongchang pig [3] that were used for *de novo*

assemblies to roughly 10 × depth coverage. We also randomly selected an individual of the cattle, low- and high-altitude chicken, goat and sheep and sequenced the whole genomes at ~10 × depth coverage.

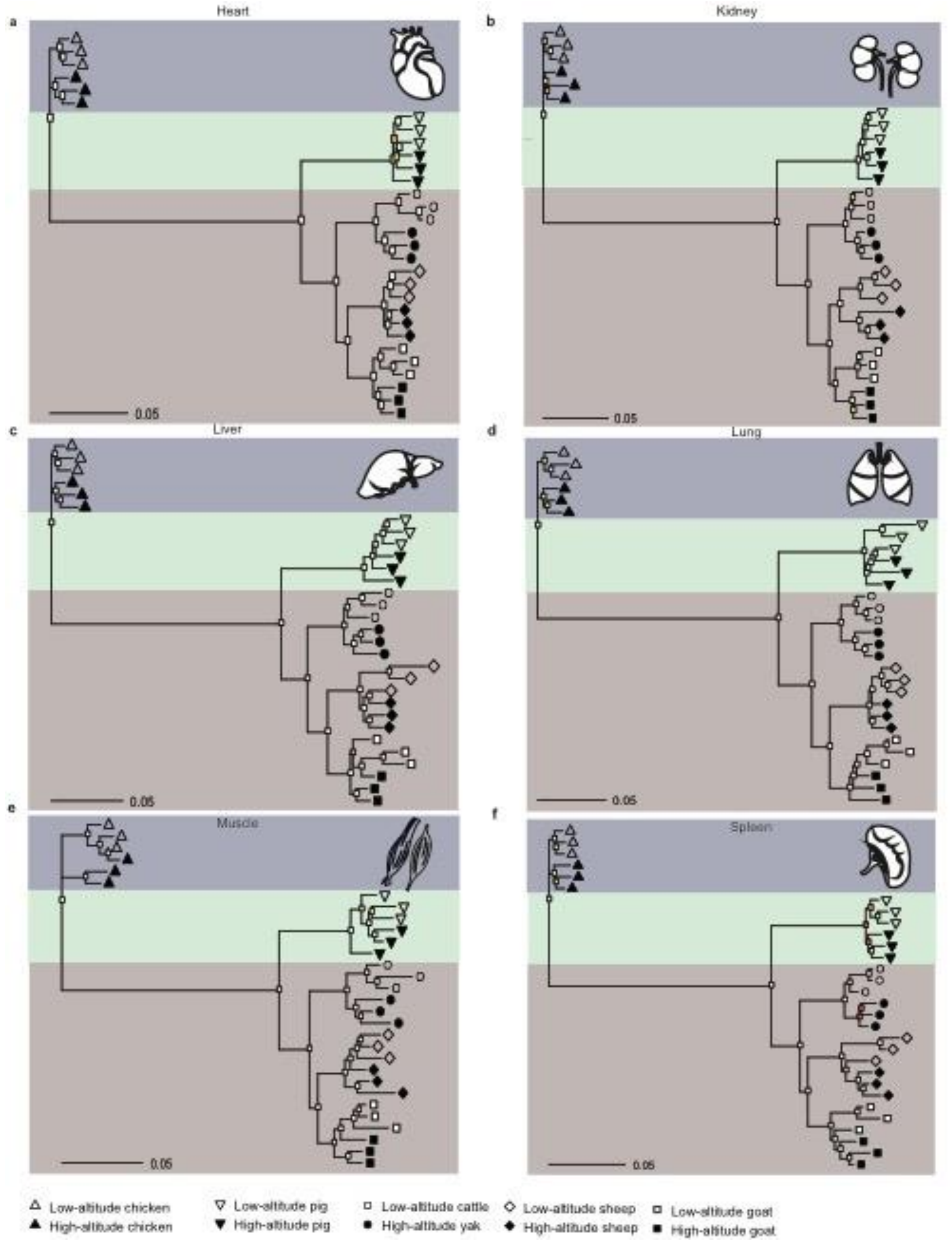


**Figure 2. Gene expression phylogenies for six tissues across five vertebrates.** Neighbour-joining expression tree constructed based on (1-Spearman correlation) distances in six tissues. We performed 100 bootstraps by randomly sampling the single copy orthologues with replacement. Bootstrap values (fractions of replicate trees that have the branching pattern as in the shown tree constructed using all the single copy orthologues) are indicated by different colors: red color of the node indicates support from

less than 50% bootstraps, while orange, yellow and white colors indicate support between 50% and 70%, between 70% and 90% and more than 90%, respectively.

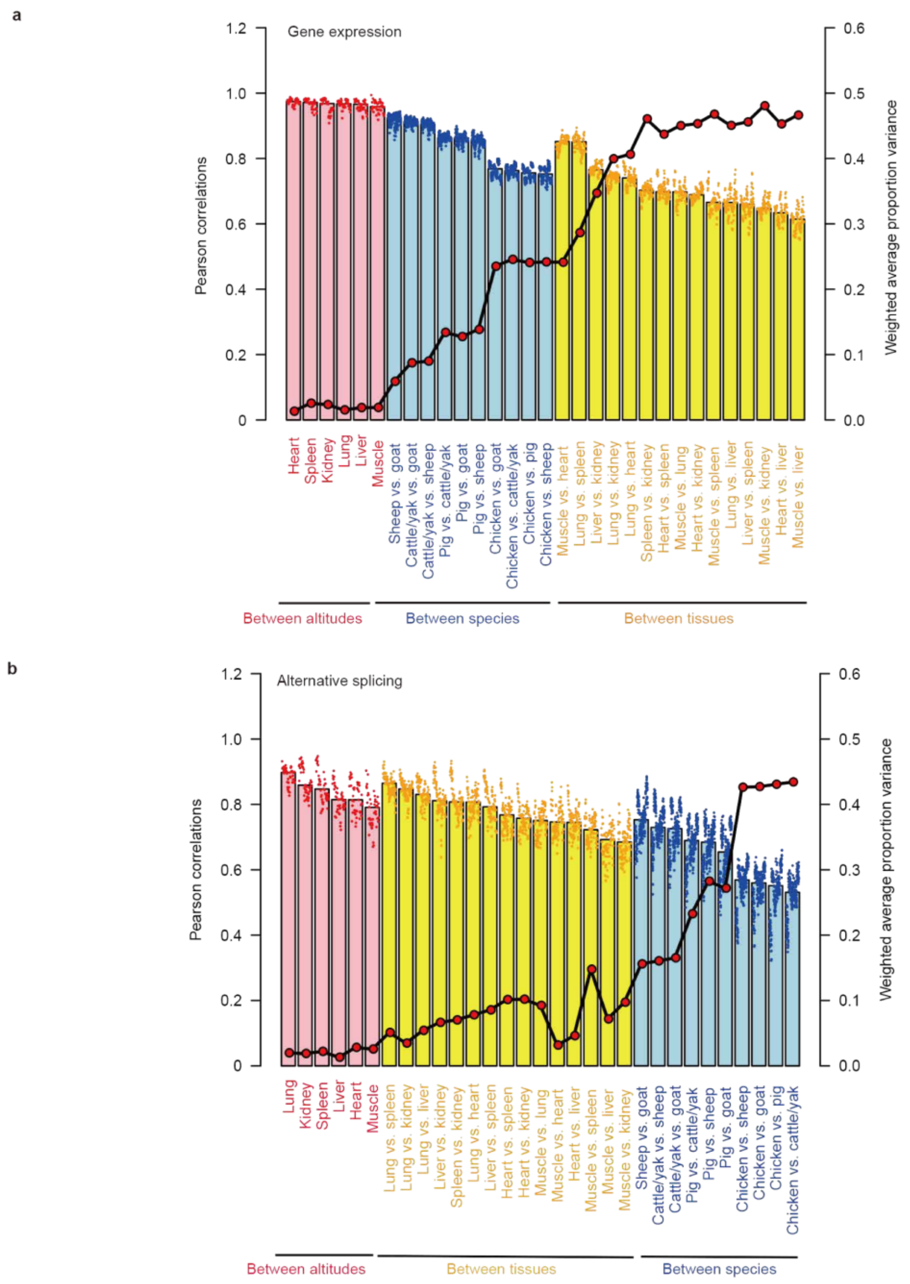


**Figure 3. Comparison of variations between altitudes, species and tissues revealed by (a) gene expression and (b) alternative splicing pattern.**

Scatter-point and bar plots represent the pairwise Pearson's correlation between samples. Weighted average proportion variance of the alternative splicing (reflected by PSI values) were determined using the Principal Variance Component Analysis (PVCA) approach and depicted as red dots connected by black lines.

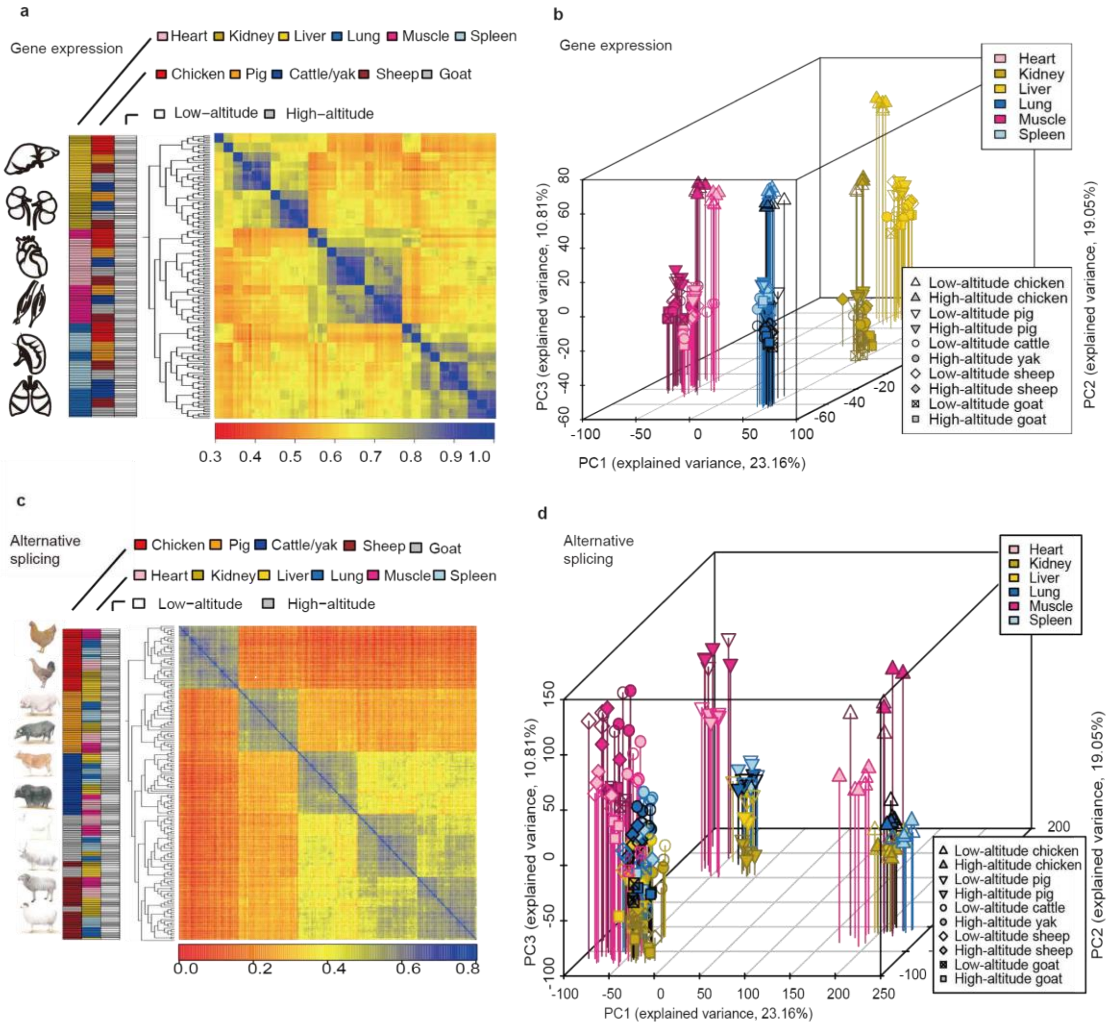


**Figure 4. Global pattern of gene expression and alternative splicing pattern.** Hierarchical clustering of samples using **(a)** the gene expression and **(c)** the alternative splicing (reflected by PSI values). Average linkage hierarchical clustering was used with distance between samples measured by the Pearson's correlation between the vectors of expression values. Factorial map of the principal-component analysis (PCA) of **(b)** gene expression levels and **(d)** the alternative splicing. The proportion of the variance explained by the principal components is indicated in parentheses.

**Table 1. Number of DEGs between five high-altitude vertebrates and their low-altitude relatives for each tissue**

| Species | Heart | Kidney | Liver | Lung | Muscle | Spleen | Mean |
|---|---|---|---|---|---|---|---|
| Chicken | 1357 (8.76%) | 784 (5.06%) | 651 (4.20%) | 1142 (7.37%) | 191 (1.23%) | 1011 (6.52%) | 856 (5.52%) |
| Pig | 225 (1.04%) | 572 (2.65%) | 1218 (5.64%) | 453 (2.10%) | 421 (1.95%) | 1015 (4.70%) | 651 (3.01%) |
| Cattle/yak | 1692 (8.47%) | 1868 (9.35%) | 938 (4.69%) | 3229 (16.16%) | 2520 (12.61%) | 2369 (11.86%) | 2103 (10.52%) |
| Sheep | 1371 (6.56%) | 4014 (19.20%) | 271 (1.30%) | 1881 (9.00%) | 1136 (5.43%) | 2458 (11.76%) | 1855 (8.87%) |
| Goat | 2401 (10.85%) | 3973 (17.95%) | 729 (3.29%) | 1461 (6.60%) | 2597 (11.73%) | 1421 (6.42%) | 2097 (9.48%) |
| Mean | 1409 (7.13%) | 2242 (10.84%) | 761 (3.83%) | 1633 (8.25%) | 1373 (6.59%) | 1655 (8.25%) | |

Percentage of the DGEs compared with the total number of annotated protein coding genes in their respective reference genomes are listed in parenthesis. There are 15495, 21594, 19981, 22131, 20908 annotated protein coding genes in reference genomes of Chicken (Galgal4) [4], pig (Suscrofa 10.2) [5], cattle (UMD3.1) [6], goat (CHIR_1.0) [7] and sheep (Oar_v3.1) [8], respectively.

 Click here to download Table Table_1.docx

**Table 1. Number of DEGs between five high-altitude vertebrates and their low-altitude relatives for each tissue**

| Species | Heart | Kidney | Liver | Lung | Muscle | Spleen | Mean |
|---|---|---|---|---|---|---|---|
| Chicken | 1357 (8.76%) | 784 (5.06%) | 651 (4.20%) | 1142 (7.37%) | 191 (1.23%) | 1011 (6.52%) | 856 (5.52%) |
| Pig | 225 (1.04%) | 572 (2.65%) | 1218 (5.64%) | 453 (2.10%) | 421 (1.95%) | 1015 (4.70%) | 651 (3.01%) |
| Cattle/yak | 1692 (8.47%) | 1868 (9.35%) | 938 (4.69%) | 3229 (16.16%) | 2520 (12.61%) | 2369 (11.86%) | 2103 (10.52%) |
| Sheep | 1371 (6.56%) | 4014 (19.20%) | 271 (1.30%) | 1881 (9.00%) | 1136 (5.43%) | 2458 (11.76%) | 1855 (8.87%) |
| Goat | 2401 (10.85%) | 3973 (17.95%) | 729 (3.29%) | 1461 (6.60%) | 2597 (11.73%) | 1421 (6.42%) | 2097 (9.48%) |
| Mean | 1409 (7.13%) | 2242 (10.84%) | 761 (3.83%) | 1633 (8.25%) | 1373 (6.59%) | 1655 (8.25%) | |

Percentage of the DGEs compared with the total number of annotated protein coding genes in their respective reference genomes are listed in parenthesis. There are 15495, 21594, 19981, 22131, 20908 annotated protein coding genes in reference genomes of Chicken (Galgal4) [4], pig (Suscrofa 10.2) [5], cattle (UMD3.1) [6], goat (CHIR_1.0) [7] and sheep (Oar_v3.1) [8], respectively.

Figure_1

Click here to download Figure Figure1.pdf

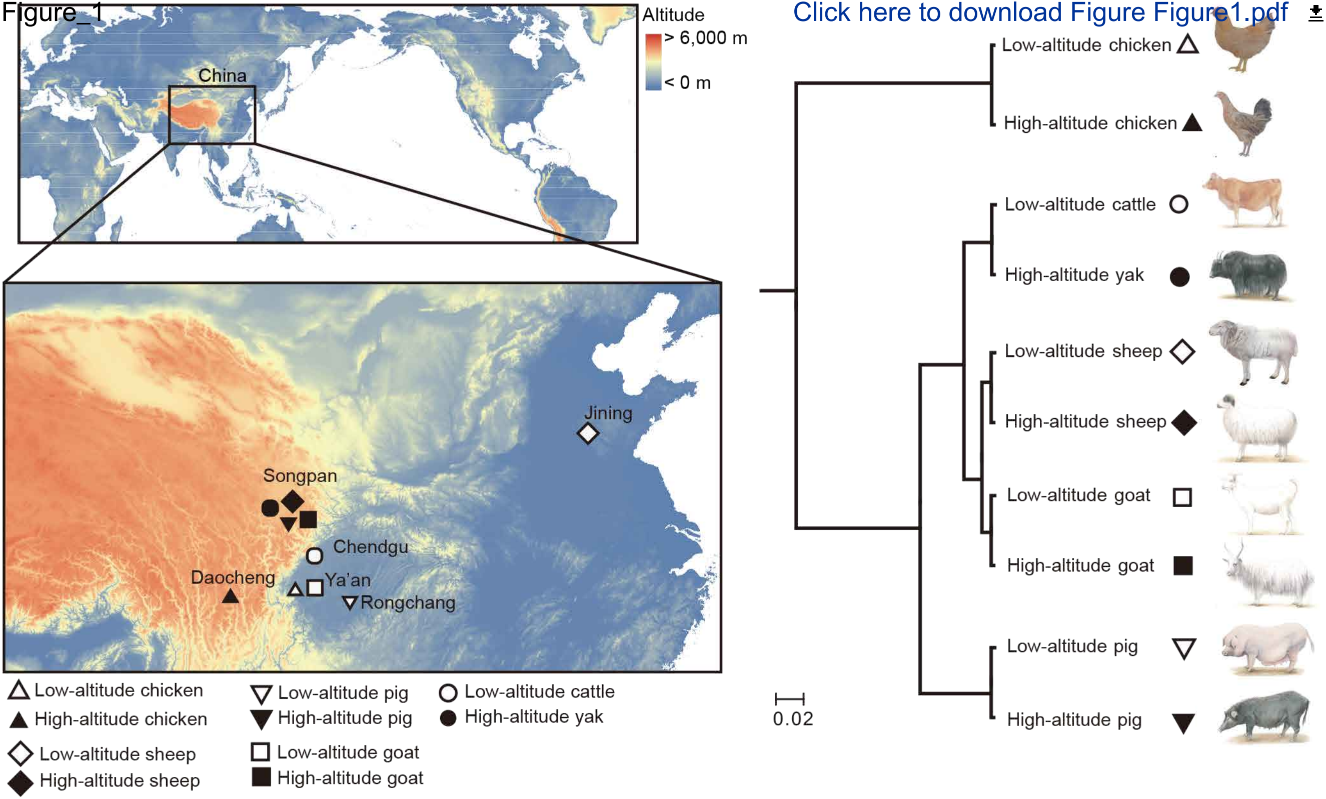

Figure_2

Click here to download Figure Figure2.pdf

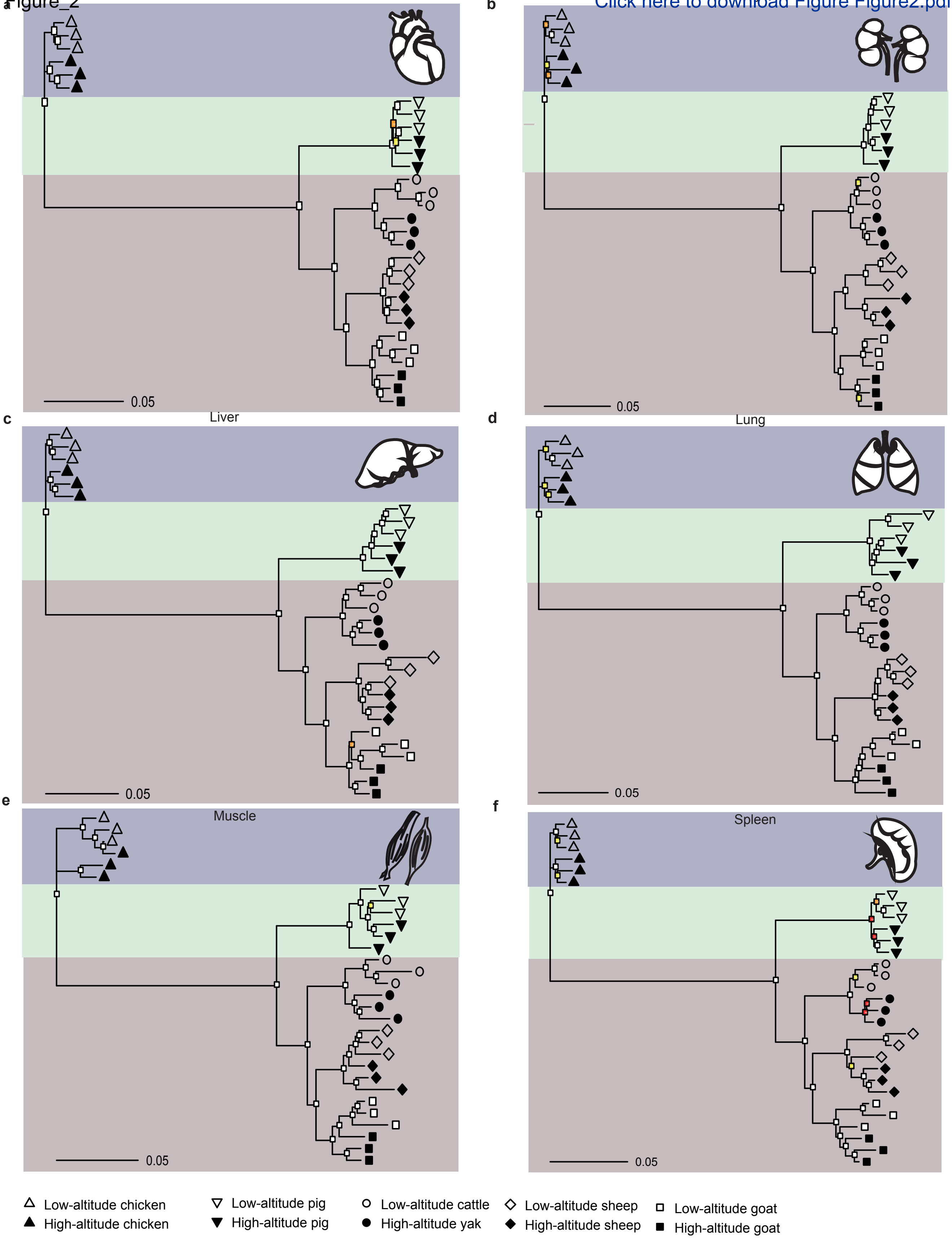

Figure_3

Click here to download Figure Figure3.pdf

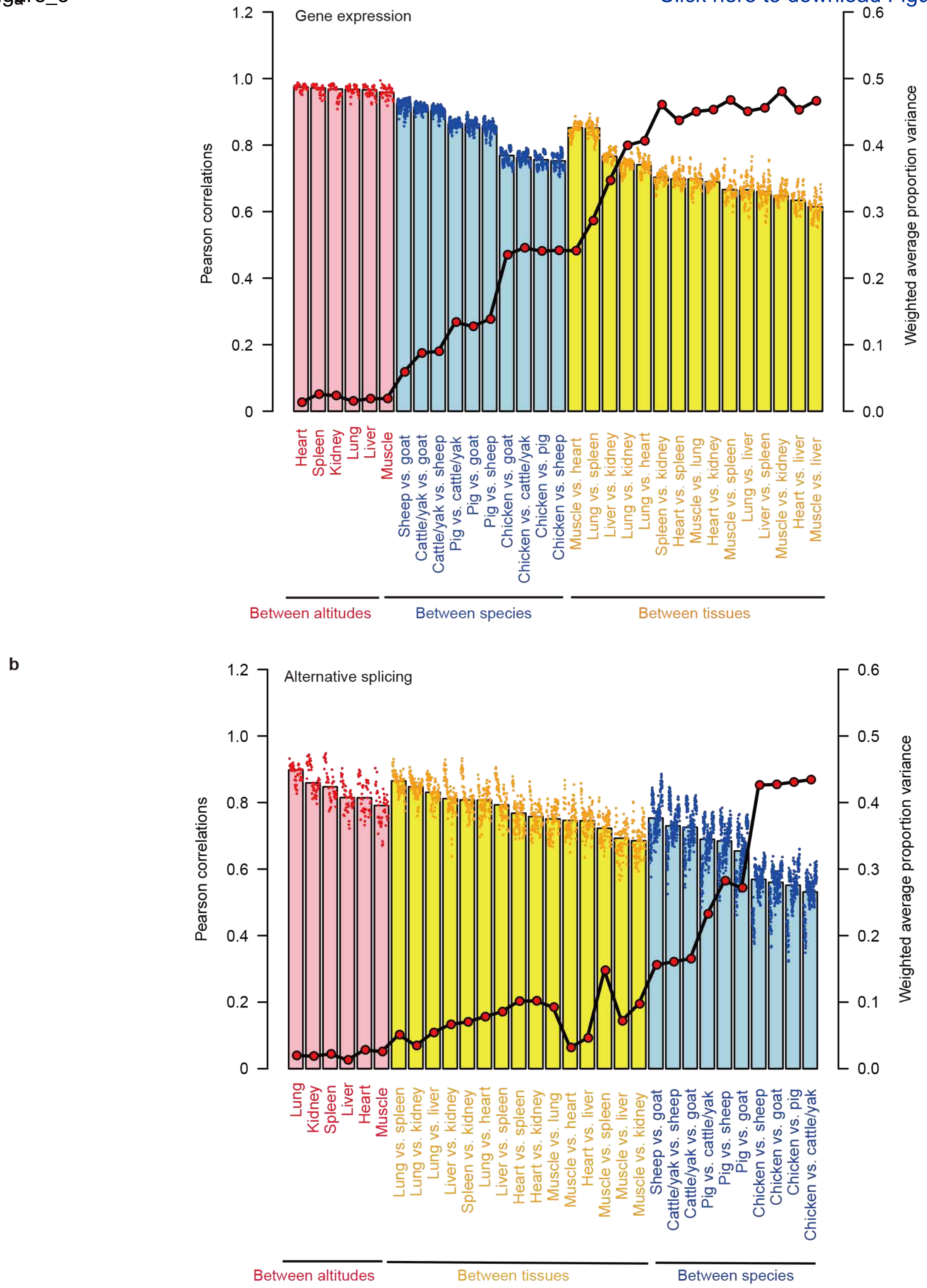

Figure_4

Click here to download Figure Figure4.pdf

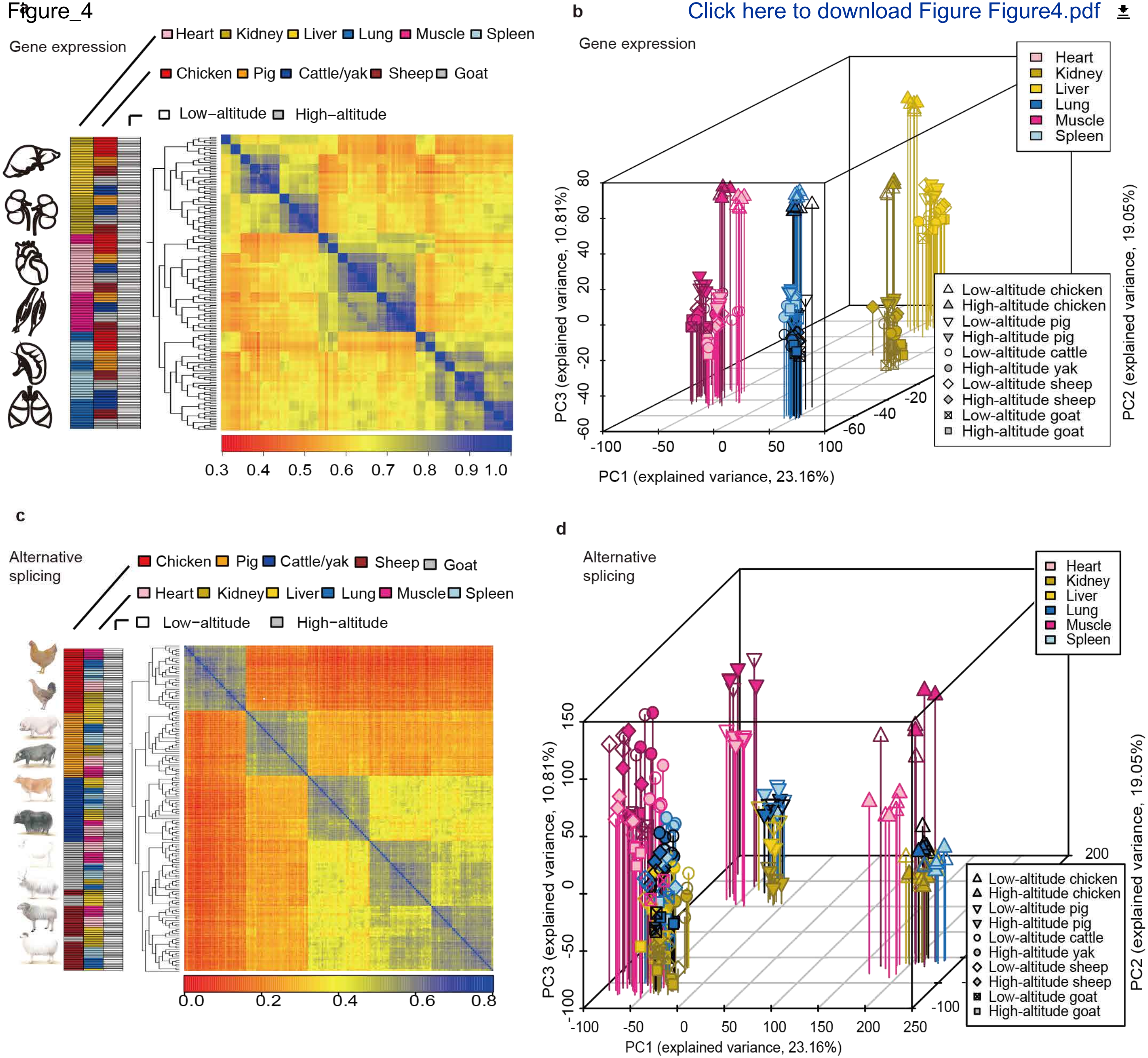

Additional_file_1

Click here to access/download
**Supplementary Material**
Additional_file_1.pdf

Additional_file_2

Click here to access/download
**Supplementary Material**
Additional_file_2.xlsx

 Click here to download Personal Cover CoverLetter.docx

Dear Editor,

We are pleased to submit our manuscript entitled ‘***Comparative transcriptomics of five high-altitude vertebrates and their low-altitude relatives***’ for consideration of publication in *GigaScience as a Data Note*.

A major objective of evolutionary genetics is to provide a mechanistic account of the genetic basis for inter- and intra-species phenotypic variation. Although the relative contribution of changes in gene regulation to adaptation continues to be debated, it has become clear that variation in gene expression patterns often plays a key part in the evolution of morphological phenotypes. The current challenge is to understand what extent the environment affects gene expression phenotypes and whether it would be possible to detect genetic contributions to variation in gene regulation within or between species.

High-altitude livings undergo strong selective pressures by the extremely inhospitable environments, making these attractive species valuable resources for comparatively investigating the adaptive evolution. To fully characterize the transcriptomic differences between distinct species in adaptation or acclimatization to high-altitude environments, so as to further comprehensively investigate evolutionary changes in different regulatory genetic mechanisms. We generated a total of ~909.6 Gb high-quality RNA-seq data using Illumina sequencing technology from 180 samples derived from six biologically representative tissues (heart, kidney, liver, lung, skeletal muscle and spleen) of three unrelated adult females for each of five agriculturally important high-altitude vertebrates (Tibetan chicken, Tibetan pig, Tibetan sheep, Tibetan goat and yak), and their cross-fertile relatives lived in the geographically neighboring low-altitude regions. Comparative transcriptomics analysis preliminarily reveals the distinctly transcriptomic characteristics between gene expression and alternative splicing. We also identified numerous differentially expressed genes were potentially involved in phenotypic divergence shaped by high-altitude adaptation.

We believe the particularly valuable RNA-seq dataset described by this manuscript will be of interest and potentially be useful to a broad range of *GigaScience* readers as it pertains to two important research areas (i.e., gene regulation and adaptive evolution) as well as the agri-food industry.

This manuscript includes 4 figures comprising 14 separate panels in total, and a table, and 8 Supplemental Figures and 3 Supplemental Tables. The manuscript has been seen and approved by all listed authors.

We hope the Editorial Board will find this study worthy of review and consideration for publication in *GigaScience as a Data Note*.

With best regards,

Dr. Mingzhou Li

Professor of Animal Genetics and Genomics

Sichuan Agricultural University, Sichuan, China

E-mail: mingzhou.li@sicau.edu.cn

**Suggested Reviewers:**

1. Prof. Marc Kirschner

   Email: marc@hms.harvard.edu

   Department of Systems Biology,

   Harvard Medical School, USA.

   Research area: Systems biology, Transcriptomics

2. Prof. Menghua Li

   Email: menghua.li@ioz.ac.cn

CAS Key Laboratory of Animal Ecology and Conservation Biology,

Institute of Zoology,

Chinese Academy of Sciences (CAS), China

Research area: Ecological and evolutionary genomics

3. Prof. Sudhir Kumar

Email: s.kumar@temple.edu

Department of Biology,

Institute for Genomics and Evolutionary Medicine,

Temple University, USA.

Research area: Phylogenomics and phylomedicine

**Excluded reviewers:**

1. Prof. Martien A. M. Groenen

Email: martien.groenen@wur.nl

Animal Breeding and Genomics Centre,

Wageningen University, The Netherlands.

2. Prof. Yaping Zhang

Email: zhangyp@mail.kiz.ac.cn

Kunming Institute of Zoology,

Chinese Academy of Sciences, China.
