# Supplementary material for: Comparative transcriptomics of 5 high-altitude vertebrates and their low-altitude relatives
Source: Gigascience. 2017 Nov 15;6(12):1–9. doi: 10.1093/gigascience/gix105 (PMC5729692; doi:10.1093/gigascience/gix105)
Supplement: GIGA-D-17-00037_Revision-1.pdf [file gix105_giga-d-17-00037_revision-1.pdf]

GigaScience

Comparative transcriptomics of five high-altitude vertebrates and their low-altitude relatives

--Manuscript Draft--

**Manuscript Number:**	GIGA-D-17-00037R1
**Full Title:**	Comparative transcriptomics of five high-altitude vertebrates and their low-altitude relatives
**Article Type:**	Data Note

Funding Information:

National High Technology Research and Development Program of China (863 Program) (2013AA102502)	Prof. Mingzhou Li
National Natural Science Foundation of China (31402046)	Dr Qianzi Tang
National Natural Science Foundation of China (31522055)	Prof. Mingzhou Li
National Natural Science Foundation of China (31601918)	Dr Jideng Ma
National Natural Science Foundation of China (31530073)	Prof Xuewei Li
National Natural Science Foundation of China (31472081)	Prof. Mingzhou Li
Science & Technology Support Program of Sichuan (2016NYZ0042)	Dr Yiren Gu
Youth Science Fund of Sichuan (2017JQ0011)	Dr Yiren Gu
China Postdoctoral Science Foundation (2015M572486)	Dr Qianzi Tang
China Agriculture Research System (CARS-36)	Dr Yiren Gu
Program for Innovative Research Team of Sichuan Province (2015TD0012)	Prof. Mingzhou Li
Program for Pig Industry Technology System Innovation Team of Sichuan Province (SCCXTD-005)	Dr Yiren Gu
Project of Sichuan Education Department (15ZA0008)	Dr Xun Wang
Project of Sichuan Education Department (15ZA0003)	Dr Miaomiao Mai
Project of Sichuan Education Department (16ZA0025)	Dr Jideng Ma
Project of Sichuan Education Department (16ZB0037)	Dr An'an Jiang
National Program for Support of Top-notch Young Professionals	Prof. Mingzhou Li
Young Scholars of the Yangtze River	Prof. Mingzhou Li

Abstract:

Background: Species living at high altitude are subject to strong selective pressures due to inhospitable environments (e.g., hypoxia, low temperature, high solar radiation, and lack of biological production), making these species valuable models for comparative analyses of local adaptation. Studies that examined high-altitude adaptation identified a vast array of rapidly evolving genes that characterize the dramatic phenotypic changes in high-altitude animals. However, how high-altitude environment shapes gene expression programs remains largely unknown.

Powered by Editorial Manager® and ProduXion Manager® from Aries Systems Corporation

	Findings: We generated a total of 910 Gb high-quality RNA-seq data for 180 samples derived from six tissues of five agriculturally important high-altitude vertebrates (Tibetan chicken, Tibetan pig, Tibetan sheep, Tibetan goat and yak), and their cross-fertile relatives living in geographically neighboring low-altitude regions. Of these, ~75% reads could be aligned to their respective reference genomes, and on average ~70% of annotated protein coding genes in each organism showed FPKM expression values greater than 0.1. We observed a general concordance in topological relationships between the nucleotide alignments and gene expression-based trees. Tissue and species accounted for markedly more variance than altitude based on either the expression or the alternative splicing patterns. Cross-species clustering analyses showed a tissue-dominated pattern of gene expression, and a species-dominated pattern for alternative splicing. We also identified numerous differentially expressed genes were potentially involved in phenotypic divergence shaped by high-altitude adaptation.<br>Conclusions: This data serves as a valuable resource for examining the convergence and divergence of gene expression changes between species as they adapt or acclimatize to high-altitude environments.<br>Keywords: high-altitude vertebrates, comparative transcriptomics, gene expression, alternative splicing
**Corresponding Author:**	Mingzhou Li, Ph.D.<br>Sichuan Agricultural University<br>Chengdu, Sichuan CHINA
**Corresponding Author Secondary Information:**	
**Corresponding Author's Institution:**	Sichuan Agricultural University
**Corresponding Author's Secondary Institution:**	
**First Author:**	Qianzi Tang
**First Author Secondary Information:**	
**Order of Authors:**	Qianzi Tang
	Yiren Gu
	Xuming Zhou
	Long Jin
	Jiuqiang Guan
	Rui Liu
	Jing Li
	Keren Long
	Shilin Tian
	Tiandong Che
	Silu Hu
	Yan Liang
	Xuemei Yang
	Xuan Tao
	Zhijun Zhong
	Guosong Wang
	Xiaohui Chen
	Diyan Li
	Jideng Ma

	Xun Wang
	Miaomiao Mai
	An'an Jiang
	Xiaolin Luo
	Xuebin Lv
	Vadim N. Gladyshev
	Xuewei Li
	Mingzhou Li, Ph.D.
**Order of Authors Secondary Information:**	
**Response to Reviewers:**	Reviewer 1:<br><br>Comment 2-1<br>The authors report a well-developed project to better understand the gene expression differences in multiple tissues from 5 species (with the cattle-yak comparison counted as one). The data collected is enormous and clearly appears to be sufficient for the analyses proposed, but there are a number of questions regarding both the methods and results presented.<br><br>Response 2-1<br>Thank you for your positive comments. We would fully address your concerns and provide our point-to-point responses as follows.<br><br>Comment 2-2<br>Of highest importance, the authors present a set of analyses in which the output is a list of genes and a calculated expression level; these lists are then used in a number of ways to calculate expression and enriched function per tissue in several comparison. These lists (and not even the numbers of genes in each list) are not provided, so it is impossible to see these lists or use the lists as a resource for other work. Since one aspect of this publication would be as a resource for others, the authors must provide these lists as well as the calculated expression value for each gene. I realize these lists are extensive, but are a crucial component of the resource, especially for those readers who will not start with the raw data, but also for those who can repeat the analyses and compare their resulting normalized expression data with those that the authors created.<br><br>Response 2-2<br>Thank you for your reminder. According to the submission guidelines of GigaScience, we uploaded the complete gene lists with normalized expression values to the GigaScience temporary FTP server.<br><br>Comment 2-3<br>Further, the authors describe some biological results on comparisons between high and low altitude, but fail to provide sufficient description of the results. The Supplementary file is incomplete (see below), but also the text on all tissue and species comparisons is only a few sentences. More is needed to justify this reporting. For example, a strength of the work is the multi-species comparison of the same question of adaptation to high altitude. A comparison of the high/low differentially expressed gene lists in the same tissue across species would seem minimal and potentially very interesting- i.e., are the genes and pathways identified similar (more similar than expectation?). This would provide more insight, as well as more evidence the analyses are providing biologically relevant information.<br><br>Response 2-3<br>Thank you for your valuable suggestions. Based on your suggestions, we evaluated the amount of shared DE genes between the high- and low-altitude populations in each tissue among five vertebrates (Supplemental Figs. S9–10 and Additional File 3), and found that more closely related vertebrates shared more common DE genes (Supplemental Fig. S11). We also discovered that the enriched functional categories of DE genes substantially overlapped (Supplemental Figs. S12–13 and Additional File 4).

We added Supplemental Figs. S9–13 and Additional Files 3–4 to the manuscript.

As shown in the newly added Supplemental Figs. S9-13 and Additional Files 3-4, expectedly, the more closely related vertebrates (Fig. 1) shared more DE genes (Supplementary Figs. S9–10 and Additional File 3). Compared with shared DE genes among mammals, especially between the two closely related members of Caprinae (goat and sheep), the birds (chickens) exhibited significantly fewer shared DE genes with mammals (Wilcoxon rank sum test, P<0.0021) (Supplementary Fig. S11). We also identified significantly enriched functional gene categories of DE genes (Chi-square test or Fisher's exact test, P<1.03 × 10-4), which were shared among multiple pairwise comparisons (Supplementary Figs. S12–13 and Additional File 4), that were potentially related to the dramatic phenotypic changes shaped by high-altitude adaptation, such as response to hypoxia (typically, 'oxidation reduction', 'heme binding', 'oxygen binding' , 'oxygen transport' and 'oxygen transporter activity'), cardiovascular system ('angiogenesis' and 'positive regulation of angiogenesis'), the efficiency of biomass production in the resource-poor highland ('metabolic pathways', 'cholesterol biosynthetic process' and 'steroid metabolic process') as well as immune response ('responses of immune and defense') (Additional file 2) (the statement has been added to the main text, page 11, line 251-267).

Comment 2-4

1. Criterion for expression.

a) On line 40, the authors indicate they are using a FPKM of 0.1. I was unable to find specific details on the sequencing data so that I could determine the number of counts this represents. I could not find the read length nor whether this was SE or PE. Assuming 100 nt read length and PE for the average of 5 Gb for each tissue reported, a FPKM of 0.1 is 2.5 counts for a 1 kb transcript. This is very low. The authors should justify this low cutoff, which affects all subsequent analyses. I would like to see the median expression level for each tissue, as well.

Response 2-4

Thank you for your valuable suggestions. Our data are paired-end reads of 100 nt for three tissues (heart, lung, and muscle), and 125 nt for the other three tissues (kidney, liver, and spleen). Although some previous reports used FPKM >0.1 as the cutoff for transcribed genes [1-3], based on your suggestions, we used a stricter cut-off of FPKM>0.5 (>0.5 FPKM for over 80% of the samples) in the subsequent analyses and updated all of the figures and tables. Our findings did not conflict with those in the initial manuscript, and were further strengthened, typically the 3D PCA result: chickens formed a distinct cluster from the mammals, which indicates that divergence in gene expression among these species started to surpass that between different tissues around when birds diverged from mammals (approximately 300 million years). We revised the corresponding text from "The exceptions to tissue dominance were that chicken heart, lung and liver clustered with chicken skeletal muscle, spleen and kidney, respectively, rather than with their mammalian counterparts, which implied that divergence in gene expression among these species started to surpass those between different tissues at about the time when birds split from mammals (~300 million years)" to "Notably, tissues of birds (chickens) formed a distinct cluster, rather than with their mammalian counterparts, which indicates that divergence in gene expression among these species started to surpass that between different tissues around when birds diverged from mammals (approximately 300 million years ago)." (Main text, page 10, lines 232-236). After adding the FPKM 0.5 cut-off filtering for genes and 5 as the gene number cut-off for enriched terms, some of the specific over-represented terms changed even though the enriched general categories remained unchanged. We have revised the corresponding text from "As expected, respectable significantly enriched functional gene categories by DGEs, which shared in multiple pair-wise comparisons, were potentially related to the dramatic phenotypic changes shaped by high-altitude adaptation, such as response to hypoxia (typically, 'oxidation reduction', 'heme binding', 'oxygen binding' , 'response to oxygen levels' and 'response to hypoxia'), cardiovascular system ('blood vessel development', 'blood vessel morphogenesis', 'blood circulation' and 'development of lung and heart'), the efficiency of biomass production in the resource-poor highland (processes of 'steroid biosynthesis' and 'fatty acid metabolism') as well as immune response ('responses of immune and defense')" to "Expectedly, the more closely related vertebrates (Fig. 1) shared more DE genes (Supplementary Figs. S9–10 and Additional File 3). Compared with shared DE genes among mammals, especially between the two closely related members of Caprinae

	(goat and sheep), the birds (chickens) exhibited significantly fewer shared DE genes with mammals (Wilcoxon rank sum test, P<0.0021) (Supplementary Fig. S11). We also identified significantly enriched functional gene categories of DE genes (Chi-square test or Fisher's exact test, P<1.03 × 10-4), which were shared among multiple pairwise comparisons (Supplementary Figs. S12–13 and Additional File 4), that were potentially related to the dramatic phenotypic changes shaped by high-altitude adaptation, such as response to hypoxia (typically, 'oxidation reduction', 'heme binding', 'oxygen binding' , 'oxygen transport' and 'oxygen transporter activity'), cardiovascular system ('angiogenesis' and 'positive regulation of angiogenesis'), the efficiency of biomass production in the resource-poor highland ('metabolic pathways', 'cholesterol biosynthetic process' and 'steroid metabolic process') as well as immune response ('responses of immune and defense') (Additional file 2)." (Main text, page 11, lines 251-267). We also revised the corresponding text from "Of these, ~75% reads could be aligned to their respective reference genomes, and on average ~70% of annotated protein coding genes in each organism showed FPKM expression values greater than 0.1" to "Of these, ~75% reads could be aligned to their respective reference genomes, and on average ~60% of annotated protein coding genes in each organism showed FPKM expression values greater than 0.5" (Main text, page 2, lines 40-41); from "Log2-transformed values of (FPKM + 1) for genes were used in subsequent analyses" to "Log2-transformed values of (FPKM + 1) for genes with >0.5 FPKM in over 80% of the samples were used in subsequent analyses" (Main text, page 5, lines 113-114); from "We found that on average 69.7% annotated protein coding genes in each genome had FPKM expression values greater than 0.1" to "We found that on average 61.2% annotated protein coding genes in each genome had FPKM expression values greater than 0.5" (Main text, page 8, lines 181-183); from "The gene expression-based tree based 7,125 single-copy orthologous genes for each tissue showed a highly consistent topology to the nucleotide sequence alignment-based phylogeny" to "The gene expression-based tree based 4,746 transcribed single-copy orthologous genes (66.61% of 7125) for each tissue showed a highly consistent topology to the nucleotide sequence alignment-based phylogeny (Fig. 2, Supplementary Methods) [9]" (Main text, page 8, lines 189-192); from "Through comparison of expression levels of 7,125 single-copy orthologous genes" to "Through comparison of expression levels of 4,746 transcribed single-copy orthologous genes" (Main text, page 9, lines 200-201); from "For gene expression, there were critical biological differences among tissues (Pearson's r = 0.71 and weighted average proportion variance = 0.42), followed by species (Pearson's r = 0.84, weighted average proportion variance = 0.16) and local adaptation (Pearson's r = 0.97 and weighted average proportion variance = 0.019)" to "For gene expression, there were critical biological differences among tissues (Pearson's r = 0.67 and weighted average proportion variance = 0.36), followed by species (Pearson's r = 0.75, weighted average proportion variance = 0.22) and local adaptation (Pearson's r = 0.95 and weighted average proportion variance = 0.019)" (Main text, page 9, lines 206-210); from "We identified ~1,512 DEGs between 30 low-versus high-altitude pairs (225 DEGs in liver of pigs to 4,014 DEGs in kidney of sheep) (Table 1). Notably, among five pairs of vertebrate, the highly-diverged yak and cattle exhibited the highest number of DEG (~2,242) across six tissues. Among six tissues, the highly aerobic kidney exhibited the highest number of DEGs (~2,103) across five pairs of vertebrates." to "We identified ~1,423 DEGs between 30 low- versus high-altitude pairs (177 DEGs in muscle of chickens to 3,853 DEGs in kidney of sheep) (Table 1). Notably, among five pairs of vertebrate, the highly-diverged yak and cattle exhibited the highest number of DEG (~2,005) across six tissues. Among six tissues, the highly aerobic kidney exhibited the highest number of DEGs (~2,097) across five pairs of vertebrates" (Main text, page 11, lines 245-250).<br>The median of gene expression values (reflected by FPKM values) increased from 6.86 to 8.65, which corresponds to the increase of filtering cut-offs from 0.1 to 0.5 (Table R1 can be accessed from RL_FiguresandTables.pdf at: https://www.dropbox.com/s/shgpb4784s409zw/RL_FiguresandTables.pdf?dl=0).<br><br>Comment 2-5<br>b) On line 188, the authors use the term "high confidence single-copy orthologs" this is not defined. And is this homology based or expression based?<br><br>Response 2-5<br>Thank you for your valuable suggestions. We are sorry for our descriptive statement of approaches. We adopted the Ensemble pipeline that is more accurate than more

feasible OrthMCL method:
We applied the most recent Ensemble pipeline (www.ensemble.org/info/genome/compara/homolog_method.html) to calculate 1:1 orthologues of five species. We downloaded the corresponding protein and CDS sequences of five species from Ensemble website with the exception of goat, whose protein and CDS sequences were downloaded from Goat Genome website. The sequences of an additional outgroup species zebrafish were also downloaded from Ensemble website. The longest protein sequence for each protein coding gene was kept for further analysis. Such protein sequences were concatenated to a single fasta file and makeblastdb function of NCBI blast+ version 2.2.28 [4] was applied to generate the reference file. The concatenated protein sequence fasta file was blasted against the reference file using blastp function of NCBI blast+: in effect, each gene of six species were blasted against each other (both within and between species), using parameters -seg no -max_hsps_per_subject 1 -use_sw_tback -evalue 1e-10 -num_threads 1. Blast e-values were converted to weights based on MIN(100,ROUND (-LOG10(evalue)/2)), and Hcluster_sg (http://sourceforge.net/p/treesoft/code/HEAD/tree/) was utilized to cluster genes into families according to weights with parameters -m 750 -w 0 -s 0.34. Zebrafish was used as an outgroup species in this analysis by setting zebrafish genes to value 2 and non-zebrafish genes to value 1 in the category file, which was integrated into the analysis via –C option. Large clusters with more than 400 genes were recursively split into sub-clusters by QuickTree version 1.1 [5] until the largest sub-cluster contained less than 400 genes. In detail, multiple sequences of each large cluster were first aligned via Mafft version 7.149b [6] with parameter –auto and then converted to stockholm format by esl-reformat function in hmmer version 3.1b1 [7]. QuickTree were used to build unrooted tree and custom python scripts were utilized to find the branch that roughly split the tree into two parts of comparable nodes, by making sure one of the two parts contained the smallest possible number of nodes over half of the total number. This splitting process was repeated until the largest of the final sub-clusters had less than 400 genes. The split clusters were combined with the original clusters with less than 400 genes. Multiple alignment of protein sequences for each cluster was then generated by Mafft if there were over 200 genes, or by a mixture of four aligners of mafftgins_msa, muscle_msa, kalign_msa and t_coffee_msa consensified of M-coffee version 10.00.r1613 [8] if otherwise. For each aligned cluster, we back-translated the protein sequences to CDS and applied TreeBeST (http://treesoft.sourceforge.net/treebest.shtml) to build phylogenetic trees reconciled with an inputted species tree. Custom python scripts were utilized to retrieve one-to-one orthologues.

We also added the detailed method to the Supplementary Methods, hoping such information will help readers better understand our work.

Comment 2-6

2. Comparison of expression differences between high and low altitude animals and functional annotation analysis.

a) Supplemental Figure S3 shows that in some tissues there are large differences in mapping rate that are not reflected in the other altitude type. Did the authors check that mapping rate did not affect their differential expression calls? Also, please report the tissue type in this graph.

Response 2-6

As you suggested, we redrew the figures and compared the mapping ratios between low- and high-altitude populations for each vertebrate. Interestingly, we found that populations with a relatively lower mapping ratio of RNA-seq data had relatively higher genomic divergence from the reference genome (which was reflected by more SNPs based on whole-genome sequence data), and vice versa (Supplementary Fig. S3).

Thank you for pointing out that several tissues exhibited relatively lower mapping ratios. For example, hearts of high- and low-altitude pigs (Illumina HiSeq 2000 with 100-nt paired-end reads) and kidneys of low-altitude goats (Illumina HiSeq 2500 with 125-nt paired-end reads) (Supplementary Fig. S3) exhibited the lowest mapping ratios. This result indicated that the relatively lower mapping ratios may not be attributed to the idiosyncrasies of the different sequencing platforms.

We then considered that the discrepancies in mapping ratios might be attributable to bias from library construction, which can be effectively corrected during the

Powered by Editorial Manager® and ProduXion Manager® from Aries Systems Corporation

normalization steps implemented in cuffdiff [9]: to correct for library sizes (i.e., sequencing depths), FPKMs and fragment counts are scaled via the median of the geometric means of fragment counts across all libraries, as described by Anders and Huber [10].

Comment 2-7
b) In Additional File 2, a large table provided the GO/KEGG/InterPro terms and whether lists of genes with specific difference in high/low altitude expression are significantly enriched for that term. The authors should show the number of genes in the list for each comparison, or only show those with at least 5-10 genes in a list. Low representation in a pathway or term can be misleading for enrichment.

Response 2-7
Thank you for your valuable suggestions. We compared the similarities and differences of DE genes and their enriched categories between high altitude vertebrates and their low-altitude relatives within each tissue for each species (Supplementary Figs. S9-13, Additional Files 3-4). Then we retained gene lists with at least 5 genes, and updated all the relevant figure and tables accordingly.

Comment 2-8
c) More importantly, the authors do not indicate the background used for these analyses. It would be most appropriate to use the total number of genes expressed in each tissue for such analyses, so that the background reflects the genes that could possibly be shown to be differentially expressed, not the genome-wide background which is often the default.

Response 2-8
Thank you for your valuable comment. As previously reported [11-19], we used the annotated genes of whole-genome as the background for gene functional enrichment analysis in our initial submission. However, as you noted, it is more appropriate to use the genes expressed in each tissue as the background for gene functional enrichment analysis, which is more representative and could prevent the potential bias of over-representation of the tissue-specific expressed genes [20]. Based on your suggestion, we re-performed gene functional enrichment analysis by using ONLY the transcribed genes as the background, and found that the updated results were consistent with our initial results (Supplementary Figs. 12–13 and Additional Files 2, 4).

Reviewer 2:

Comment 3-1
First of all let me congratulate you and all authors for this piece of research. I have although some questions that I believe are important in order to improve your manuscript:
In Data Analysis:

Response 3-1
Thank you so much for your positive comments.

Comment 3-2
page 4, lines 85-88: may you specify how the data filtering was performed? which software did you use, or in case you have used in house developed scripts may you please provide them as supplemental information?

Response 3-2
Thank you so much for your questions. I used prinseq-0.20.4 [21], cutadapt-1.12 [22] and in house developed script to perform the filtering. The parameters used are 'prinseq-lite.pl -fastq R1.fastq -fastq2 R2.fastq -out_format 3 -ns_max_p 10 -out_good output -out_bad null', and 'cutadapt -a AGATCGGAAGAGCACACGTCTGAACTCCAGTCAC --overlap=10 --error-rate=0.1 --discard-trimmed --paired-output tmp.2.fastq -o tmp.1.fastq R1_1.fastq R2_2.fastq', 'cutadapt -a AGATCGGAAGAGCGTCGTGTAGGGAAAGAGTGTAGATCTCGGTGGTCGCCGTATCATT --overlap=10 --error-rate=0.1 --discard-trimmed --paired-output result_1_filteradapt.fastq -o result_2_filteradapt.fastq tmp.2.fastq tmp.1.fastq'

Powered by Editorial Manager® and ProduXion Manager® from Aries Systems Corporation

(Supplementary Methods).

Comment 3-3
page 4 line 93, may you specify the parameters used for the analysis performed with EnsemblComparaGeneTrees method?

Response 3-3
Thank you for your valuable suggestions. We applied the most recent Ensemble pipeline (www.ensemble.org/info/genome/compara/homolo g_method.html) to calculate 1:1 orthologues of five species. We downloaded the corresponding protein and CDS sequences of five species from Ensemble website with the exception of goat, whose protein and CDS sequences were downloaded from Goat Genome website. The sequences of an additional outgroup species zebrafish were also downloaded from Ensemble website. The longest protein sequence for each protein coding gene was kept for further analysis. Such protein sequences were concatenated to a single fasta file and makeblastdb function of NCBI blast+ version 2.2.28[4] was applied to generate the reference file. The merged protein sequence fasta file was blasted against the reference file using blastp function of NCBI blast+: in effect, each gene of six species were blasted against each other (both within and between species), using parameters -seg no -max_hsps_per_subject 1 -use_sw_tback -evalue 1e-10 -num_threads 1. Blast e-values were converted to weights based on MIN(100,ROUND (-LOG10(evalue)/2)), and Hcluster_sg (http://sourceforge.net/p/treesoft/code/HEAD/tree/) was utilized to cluster genes into families according to weights with parameters -m 750 -w 0 -s 0.34. Zebrafish was used as an outgroup species in this analysis by setting zebrafish genes to value 2 and non-zebrafish genes to value 1 in the category file, which was integrated into the analysis via –C option. Large clusters with more than 400 genes were recursively split into sub-clusters by QuickTree version 1.1 [5] until the largest sub-cluster contained less than 400 genes. In detail, multiple sequences of each large cluster were first aligned via Mafft version 7.149b [6] with parameter –auto and then converted to stockholm format by esl-reformat function in hmmer version 3.1b1 [7]. QuickTree were used to build unrooted tree and custom python scripts were utilized to find the branch that roughly split the tree into two parts of comparable nodes, by making sure one of the two parts contained the smallest possible number of nodes over half of the total number. This splitting process was repeated until the largest of the final sub-clusters had less than 400 genes. The split clusters were combined with the original clusters with less than 400 genes. Multiple alignment of protein sequences for each cluster was then generated by Mafft if there were over 200 genes, or by a mixture of four aligners of mafftgins_msa, muscle_msa, kalign_msa and t_coffee_msa consensified by M-coffee version 10.00.r1613 [8] if otherwise. For each aligned cluster, we back-translated the protein sequences to CDS and applied TreeBeST (http://treesoft.sourceforge.net/treebest.shtml) to build phylogenetic trees reconciled with an inputted species tree. Custom python scripts were utilized to retrieve one-to-one orthologues (Supplementary Methods).

Comment 3-4
page 4- line 96, may you please detail the parameters used for the BWA alignment?

Response 3-4
Thank you for the valuable suggestions. The parameters are 'bwa mem -t 10 -k 32 -M' (Supplementary Methods).

Comment 3-5
page 5- line 101- which were the parameters defined for GATK detection of SNPs and Indels? Parameters like Calling confidence and minimum read depth?

Response 3-5
Thank you for your valuable suggestions. AddOrReplaceReadGroups and BuildBamIndex function in Picard version 1.14 (http://sourceforge.net/projects/picard/) was applied to add read group information and index, separately. Indel realignment was performed using RealignerTargetCreator and IndelRealigner tools in GATK. We called variants by HaplotypeCaller, separated SNVs and Indels using SelectVariants, filtered SNVs with Fisher Strand values>60 or Qual By Depth values<2 or Mapping

Powered by Editorial Manager® and ProduXion Manager® from Aries Systems Corporation

Quality values<40 or Mapping Quality Rank Sum Test values<-12.5 or Read Position Rank Sum Test values<-8, and filtered Indels with Fisher Strand values>200 or Qual By Depth values<2 or Read Position Rank Sum Test values<-20 (Supplementary Methods).

Comment 3-6
page 5 line 108- which parameters were used for the TopHat alignment?

Response 3-6
Thank you for your valuable suggestions. The parameters we used are '--library-type fr-firststrand -p 4 --output-dir myoutputdir –G myspecies.gtf myspecies_genomeindex read1.fq.gz read2.fq.gz' (Supplementary Methods).

Comment 3-7
In Findings:
I am missing analysis that I was expecting in a study of adaptation to altitude which generated so much WGS data. I suggest that you study genetic divergence by Fst or by Tajima's D and make identification of selection footprints. It would be great then to compare the genes being harbored in selective sweeps and the changes at transcriptomic level.

Response 3-7
We greatly appreciate your valuable comments.

At present, few studies have sufficiently characterized the direct relationship between genes embedded in selected regions and expression changes. Consequently, exploring the potential impact of positive selection on gene transcription is of great interest. As far as we know, only three vertebrates have publicly available whole-genome sequences for multiple individuals of both low-altitude populations (Pengxian chickens, Rongchang pigs, and Jersey cattle) and their high-altitude relatives (Tibetan chickens, Tibetan pigs, and yak) [23-26] (Table R2 can be accessed from RL_FiguresandTables.pdf at: https://www.dropbox.com/s/shgpb4784s409zw/RL_FiguresandTables.pdf?dl=0).

To investigate the effects of positive selection on gene expression, we downloaded the above datasets and identified the genes embedded in selected regions (see Fig. R1) for high-altitude populations (Tibetan chickens, Tibetan pigs, and yak) against their low-altitude relatives (Pengxian chickens, Rongchang pigs, and Jersey cattle) (see Figs. R2–4) (Figs. R1-4 can be can be accessed from RL_FiguresandTables.pdf at: https://www.dropbox.com/s/shgpb4784s409zw/RL_FiguresandTables.pdf?dl=0).

We found the genes embedded in selected regions exhibited highly comparable expression levels between the high-altitude populations and their low-altitude relatives within each tissue for each vertebrate, which was similar (P values of Wilcoxon rank sum test range from 0.120 to 0.939) to the genes outside selected regions (see Fig. R2).

We further observed expression levels of genes embedded in selected regions are highly comparable with the genes outside selected regions within each tissue for high-altitude population of each vertebrate (P values of Wilcoxon rank sum test range from 0.297 to 0.934) (see Fig. R3), this tendency also exists in their respective low altitude relatives (P values of Wilcoxon rank sum test range from 0.346 to 0.940) (see Fig. R4).

In this study, we did not observe the effects of positive selection on gene expression, which was most likely due to the distinct functional roles of variations with highly skewed frequency spectra. Generally, SNPs can be classified as coding (synonymous, missense, and nonsense) and non-coding. It is essential to perform further functional analyses to assess the impact of variations on gene expression; it is especially necessary to decipher the impact of non-coding variations that are located in regulatory regions (in particular, promoters, enhancers, and silencers) on gene expression.

Additionally, it is worth noting that our investigation is based on different individuals and had a small sample size; further large-scale experiments with proper design would be beneficial for answering this question.

Comment 3-8
page 10 lines 230-235: Did this happen in the low altitude chicken or only in one? its hard to see this in the figure

Response 3-8

Powered by Editorial Manager® and ProduXion Manager® from Aries Systems Corporation

	Thank you for your thoughtful comment. As shown in the updated Fig. 4a and 4b (see Response 2–4), the Tibetan chickens and their low-altitude relatives formed a distinct cluster from the mammals. We revised this part of the manuscript to: "Notably, tissues of birds (chickens) formed a distinct cluster, rather than with their mammalian counterparts, which indicates that divergence in gene expression among these species started to surpass that between different tissues around when birds diverged from mammals (approximately 300 million years ago)." (Figs. 4a and 4b)<br><br>Comment 3-9<br>page 11 lines 251-259: The way these results are presented its hard to infer if the pathways affected by adaptation to altitude if these were the same between species or not. This is an important question that your results would enable to answer. I would suggest that a table per species should be made as well as venn diagrams that would lead us to understand which pathways were commonly affected or were different between species and if these were the same also at tissue level. I would like to see this part of the manuscript more enhanced, giving a larger value to the high value data that you have generated in your research.<br><br>Response 3-9<br>Thank you for your valuable suggestions, which are also commented by reviewer 1 (please see Response 2-3 as follows).<br>Thank you for your valuable suggestions. Based on your suggestions, we evaluated the amount of shared DE genes between the high- and low-altitude populations in each tissue among five vertebrates (Supplemental Figs. S9–10 and Additional File 3), and found that more closely related vertebrates shared more common DE genes (Supplemental Fig. S11). We also discovered that the enriched functional categories of DE genes substantially overlapped (Supplemental Figs. S12–13 and Additional File 4). We added Supplemental Figs. S9–13 and Additional Files 3–4 to the manuscript.<br>As shown in the newly added Supplemental Figs. S9-13 and Additional Files 3-4, expectedly, the more closely related vertebrates (Fig. 1) shared more DE genes (Supplementary Figs. S9–10 and Additional File 3). Compared with shared DE genes among mammals, especially between the two closely related members of Caprinae (goat and sheep), the birds (chickens) exhibited significantly fewer shared DE genes with mammals (Wilcoxon rank sum test, $P<0.0021$) (Supplementary Fig. S11). We also identified significantly enriched functional gene categories of DE genes (Chi-square test or Fisher's exact test, $P<1.03 \times 10\text{-}4$), which were shared among multiple pairwise comparisons (Supplementary Figs. S12–13 and Additional File 4), that were potentially related to the dramatic phenotypic changes shaped by high-altitude adaptation, such as response to hypoxia (typically, 'oxidation reduction', 'heme binding', 'oxygen binding' , 'oxygen transport' and 'oxygen transporter activity'), cardiovascular system ('angiogenesis' and 'positive regulation of angiogenesis'), the efficiency of biomass production in the resource-poor highland ('metabolic pathways', 'cholesterol biosynthetic process' and 'steroid metabolic process') as well as immune response ('responses of immune and defense') (Additional file 2) (the statement has been added to the main text, page 11, line 251-267).<br><br>1.Brooks MJ, Rajasimha HK, Roger JE and Swaroop A. Next-generation sequencing facilitates quantitative analysis of wild-type and Nrl(-/-) retinal transcriptomes. Mol Vis. 2011;17:3034-54.<br>2.Hugo W, Shi H, Sun L, Piva M, Song C, Kong X, et al. Non-genomic and Immune Evolution of Melanoma Acquiring MAPKi Resistance. Cell. 2015;162(6):1271-85. doi:10.1016/j.cell.2015.07.061.<br>3.Mele M, Ferreira PG, Reverter F, DeLuca DS, Monlong J, Sammeth M, et al. Human genomics. The human transcriptome across tissues and individuals. Science. 2015;348(6235):660-5. doi:10.1126/science.aaa0355.<br>4.Camacho C, Coulouris G, Avagyan V, Ma N, Papadopoulos J, Bealer K, et al. BLAST+: architecture and applications. BMC Bioinformatics. 2009;10:421. doi:10.1186/1471-2105-10-421.<br>5.Howe K, Bateman A and Durbin R. QuickTree: building huge Neighbour-Joining trees of protein sequences. Bioinformatics. 2002;18(11):1546-7.<br>6.Katoh K and Standley DM. MAFFT multiple sequence alignment software version 7: improvements in performance and usability. Mol Biol Evol. 2013;30(4):772-80. doi:10.1093/molbev/mst010.<br>7.Finn RD, Clements J and Eddy SR. HMMER web server: interactive sequence

Powered by Editorial Manager® and ProduXion Manager® from Aries Systems Corporation

similarity searching. Nucleic Acids Res. 2011;39(Web Server issue):W29-37. doi:10.1093/nar/gkr367.
8.Wallace IM, O'Sullivan O, Higgins DG and Notredame C. M-Coffee: combining multiple sequence alignment methods with T-Coffee. Nucleic Acids Res. 2006;34 (6):1692-9. doi:10.1093/nar/gkl091.
9.Trapnell C, Hendrickson DG, Sauvageau M, Goff L, Rinn JL and Pachter L. Differential analysis of gene regulation at transcript resolution with RNA-seq. Nat Biotechnol. 2013;31(1):46-53. doi:10.1038/nbt.2450.
10.Anders S and Huber W. Differential expression analysis for sequence count data. Genome Biol. 2010;11(10):R106. doi:10.1186/gb-2010-11-10-r106.
11.Yamaji M, Jishage M, Meyer C, Suryawanshi H, Der E, Yamaji M, et al. DND1 maintains germline stem cells via recruitment of the CCR4-NOT complex to target mRNAs. Nature. 2017;543(7646):568-72. doi:10.1038/nature21690.
12.Zhang Y, Sloan SA, Clarke LE, Caneda C, Plaza CA, Blumenthal PD, et al. Purification and characterization of progenitor and mature human astrocytes reveals transcriptional and functional differences with mouse. Neuron. 2016;89 (1):37-53. doi:10.1016/j.neuron.2015.11.013.
13.Andor N, Graham TA, Jansen M, Xia LC, Aktipis CA, Petritsch C, et al. Pan-cancer analysis of the extent and consequences of intratumor heterogeneity. Nat Med. 2016;22(1):105-13. doi:10.1038/nm.3984.
14.Treutlein B, Lee QY, Camp JG, Mall M, Koh W, Shariati SA, et al. Dissecting direct reprogramming from fibroblast to neuron using single-cell RNA-seq. Nature. 2016;534(7607):391-5. doi:10.1038/nature18323.
15.Okin D and Medzhitov R. The effect of sustained inflammation on hepatic mevalonate pathway results in hyperglycemia. Cell. 2016;165(2):343-56. doi:10.1016/j.cell.2016.02.023.
16.Pimentel H, Parra M, Gee SL, Mohandas N, Pachter L and Conboy JG. A dynamic intron retention program enriched in RNA processing genes regulates gene expression during terminal erythropoiesis. Nucleic Acids Res. 2016;44(2):838-51. doi:10.1093/nar/gkv1168.
17.Mahat DB, Salamanca HH, Duarte FM, Danko CG and Lis JT. Mammalian heat shock response and mechanisms underlying its genome-wide transcriptional regulation. Mol Cell. 2016;62(1):63-78. doi:10.1016/j.molcel.2016.02.025.
18.Ortiz-Ramirez C, Hernandez-Coronado M, Thamm A, Catarino B, Wang M, Dolan L, et al. A transcriptome atlas of physcomitrella patens provides insights into the evolution and development of land plants. Mol Plant. 2016;9(2):205-20. doi:10.1016/j.molp.2015.12.002.
19.Lacar B, Linker SB, Jaeger BN, Krishnaswami S, Barron J, Kelder M, et al. Nuclear RNA-seq of single neurons reveals molecular signatures of activation. Nat Commun. 2016;7:11022. doi:10.1038/ncomms11022.
20.Timmons JA, Szkop KJ and Gallagher IJ. Multiple sources of bias confound functional enrichment analysis of global -omics data. Genome Biol. 2015;16:186. doi:10.1186/s13059-015-0761-7.
21.Schmieder R and Edwards R. Quality control and preprocessing of metagenomic datasets. Bioinformatics. 2011;27(6):863-4. doi:10.1093/bioinformatics/btr026.
22.Martin M. Cutadapt removes adapter sequences from high-throughput sequencing reads. EMBnetJournal. 2011;17 1:10-2.
23.Qiu Q, Wang L, Wang K, Yang Y, Ma T, Wang Z, et al. Yak whole-genome resequencing reveals domestication signatures and prehistoric population expansions. Nat Commun. 2015;6:10283. doi:10.1038/ncomms10283.
24.Daetwyler HD, Capitan A, Pausch H, Stothard P, van Binsbergen R, Brondum RF, et al. Whole-genome sequencing of 234 bulls facilitates mapping of monogenic and complex traits in cattle. Nat Genet. 2014;46(8):858-65. doi:10.1038/ng.3034.
25.Ai H, Fang X, Yang B, Huang Z, Chen H, Mao L, et al. Adaptation and possible ancient interspecies introgression in pigs identified by whole-genome sequencing. Nat Genet. 2015;47(3):217-25. doi:10.1038/ng.3199.
26.Li D, Che T, Chen B, Tian S, Zhou X, Zhang G, et al. Genomic data for 78 chickens from 14 populations. GigaScience. 2017;6(6):1-5. doi:10.1093/gigascience/gix026.

**Additional Information:**	
**Question**	**Response**
Are you submitting this manuscript to a	No

special series or article collection?	
**Experimental design and statistics**<br><br>Full details of the experimental design and statistical methods used should be given in the Methods section, as detailed in our Minimum Standards Reporting Checklist. Information essential to interpreting the data presented should be made available in the figure legends.<br><br>Have you included all the information requested in your manuscript?	Yes
**Resources**<br><br>A description of all resources used, including antibodies, cell lines, animals and software tools, with enough information to allow them to be uniquely identified, should be included in the Methods section. Authors are strongly encouraged to cite Research Resource Identifiers (RRIDs) for antibodies, model organisms and tools, where possible.<br><br>Have you included the information requested as detailed in our Minimum Standards Reporting Checklist?	Yes
**Availability of data and materials**<br><br>All datasets and code on which the conclusions of the paper rely must be either included in your submission or deposited in publicly available repositories (where available and ethically appropriate), referencing such data using a unique identifier in the references and in the "Availability of Data and Materials" section of your manuscript.<br><br>Have you have met the above requirement as detailed in our Minimum Standards Reporting Checklist?	Yes

Manuscript

Click here to download Manuscript Manuscriptnew_mostrecent_noEndNote.docx

Comparative transcriptomics of five high-altitude vertebrates and their low-altitude relatives

Qianzi Tang[1†], Yiren Gu[2†*], Xuming Zhou[3†], Long Jin[1], Jiuqiang Guan[4], Rui Liu[1], Jing Li[1], Kereng Long[1], Shilin Tian[1], Tiandong Che[1], Silu Hu[1], Yan Liang[2], Xuemei Yang[2], Xuan Tao[2], Zhijun Zhong[2], Guosong Wang[1,5], Xiaohui Chen[2], Diyan Li[1], Jideng Ma[1], Xun Wang[1], Miaomiao Mai[1], An'an Jiang[1], Xiaolin Luo[4], Xuebin Lv[2], Vadim N. Gladyshev[3], Xuewei Li[1*] and Mingzhou Li[1*]

[1] Institute of Animal Genetics and Breeding, College of Animal Science and Technology, Sichuan Agricultural University, Chengdu 611130, China;

[2] Animal Breeding and Genetics Key Laboratory of Sichuan Province, Pig Science Institute, Sichuan Animal Science Academy, Chengdu 610066, China

[3] Division of Genetics, Department of Medicine, Brigham and Women's Hospital, Harvard Medical School, Boston, Massachusetts, 02115 USA;

[4] Yak Research Institute, Sichuan Academy of Grassland Science, Chengdu 610097, China;

[5] Department of Animal Science, Texas A & M University, College Station, Texas, 77843 USA.

[†] These authors contributed equally to this work.

Corresponding authors. E-mail: Mingzhou Li: mingzhou.li@sicau.edu.cn, Xuewei Li: xuewei.li@sicau.edu.cn, Yiren Gu: guyiren1128@163.com.

Abstract

Background: Species living at high altitude are subject to strong selective pressures due to inhospitable environments (e.g., hypoxia, low temperature, high solar radiation, and lack of biological production), making these species valuable models for comparative analyses of local adaptation. Studies that examined high-altitude adaptation identified a vast array of rapidly evolving genes that characterize the dramatic phenotypic changes in high-altitude animals. However, how high-altitude environment shapes gene expression programs remains largely unknown.

Findings: We generated a total of 910 Gb high-quality RNA-seq data for 180 samples derived from six tissues of five agriculturally important high-altitude vertebrates (Tibetan chicken, Tibetan pig, Tibetan sheep, Tibetan goat and yak), and their cross-fertile relatives living in geographically neighboring low-altitude regions. Of these, ~75% reads could be aligned to their respective reference genomes, and on average ~60% of annotated protein coding genes in each organism showed FPKM expression values greater than 0.5. We observed a general concordance in topological relationships between the nucleotide alignments and gene expression-based trees. Tissue and species accounted for markedly more variance than altitude based on either the expression or the alternative splicing patterns. Cross-species clustering analyses showed a tissue-dominated pattern of gene expression, and a species-dominated pattern for alternative splicing. We also identified numerous differentially expressed genes were potentially involved in phenotypic divergence shaped by high-

altitude adaptation.

Conclusions: This data serves as a valuable resource for examining the convergence and divergence of gene expression changes between species as they adapt or acclimatize to high-altitude environments.

Keywords: high-altitude vertebrates, comparative transcriptomics, gene expression, alternative splicing

Data description

Transcriptome sequencing

Six tissues (heart, kidney, liver, lung, skeletal muscle and spleen) of three unrelated adult females for each of five high-altitude vertebrates and their low-altitude relatives were sampled **(Fig. 1a** and **Supplementary Fig. S1)**. Animals were sacrificed humanely to ameliorate suffering. All animals and samples used in this study were collected according to the guidelines for the care and use of experimental animals established by the Ministry of Agriculture of China. We extracted total RNA, prepared libraries and sequenced the libraries on Illumina HiSeq 2000 or 2500 platforms. We generated a total of ~909.6 Gb high-quality RNA-seq data for 180 samples (~5.05 Gb per sample) of 30 individuals across 6 tissues **(Supplementary Table S1)**.

Whole-genome re-sequencing

To compare the phylogeny derived from gene expression with the phylogenetic relationships of the five high-altitude vertebrates and their low-altitude relatives, we constructed the phylogenetic tree based on nucleotide alignments. We extracted the unassembled reads from short-insert (500 bp) libraries of a single yak [1] (NCBI-SRA: SRX103159 to SRX103161, and

SRX103175 and SRX103176), a Tibetan pig [2] (NCBI-SRA: SRX219342) and a low-altitude Rongchang pig (NCBI-SRA: SRX1544519) [3] that were used for *de novo* assemblies to roughly 10 × depth coverage. We also randomly selected an individual of the cattle, low- and high-altitude chicken, goat and sheep, and sequenced their whole genomes at ~10 × depth coverage (NCBI-SRA: SRP096151). Genomic DNA was extracted from blood tissue of each individual. Sequencing was performed on the Illumina X Ten platform, and a total of 198.64 Gb of paired-end DNA sequence was generated **(Supplementary Table S2)**.

Data analysis

Data filtering

To avoid reads with artificial bias, we removed the following type of reads: (a) Reads with ≥ 10% unidentified nucleotides (N); (b) Reads with > 10 nt aligned to the adapter, allowing ≤ 10% mismatches; (c) Reads with > 50% bases having phred quality < 5.

Identification of single-copy orthologous genes

Single-copy orthologous genes across five reference genomes, i.e. chicken (Galgal4) [4], pig (Suscrofa 10.2) [5], cattle (UMD3.1) [6], goat (CHIR_1.0) [7] and sheep (Oar_v3.1) [8] were determined using a EnsemblCompara GeneTrees method [9] **(Supplementary Fig. S2, Supplementary Methods) [9].**

Construction of phylogenetic tree based on nucleotide alignments

High-quality re-sequencing data were mapped to their respective reference

genomes using BWA software (version 0.7.7) [10], reads with mapping quality > 0 were retained and potential PCR duplication cases were removed. For each individual, ~97.01% of reads were mapped to ~97.40% (at least 1 × depth coverage) or ~91.86% (at least 4 × depth coverage) of the reference genome assemblies **(Supplementary Table S2)**. Single nucleotide variations (SNVs) and insertion and deletions (InDels) were further detected by following GATK's best practice (version 3.3-0) [11]. We substituted SNVs and InDels identified in our study in the coding DNA sequences (CDS) of the respective reference genomes. Single copy orthologues with substituted CDS of the five vertebrates were applied to Treebest [12] and generating the neighbor-joining tree **(Fig. 1b)**.

Analyses of gene expression

High-quality RNA-seq reads were mapped to their respective reference genomes using Tophat (version 2.0.11) [13]. Cufflinks (version 2.2.1) [14] was applied to quantify gene expression and obtain FPKM expression values. We generated abundance files by applying Cuffquant (part of Cufflinks) to read mapping results. Log_2-transformed values of (FPKM + 1) for genes with >0.5 FPKM in over 80% of the samples were used for subsequent analyses.

Pearson's correlations were calculated across six samples from low- and high-altitudes populations within each group of specific tissue and animals; among pairwise comparisons of five animals within each of the six tissues; and among pairwise comparisons of six tissues within each of the five animals. Principal Variance Component Analysis (PVCA) was carried out using R package pvca [15]. Neighbor-joining expression-based trees were generated according to distance matrices composed of pairwise (1-Spearman's correlations) implemented in R package ape [16]. Reproducibility of branching

patterns was estimated by bootstrapping genes, that is, the single copy orthologues were randomly sampled with replacement 100 times. The fractions of replicate trees that share the branching patterns of the original tree constructed were marked by distinct node colors in the figure.

We generated abundance files by applying Cuffquant (part of Cufflinks) to read mapping results, and further applied abundance files to Cuffdiff (part of Cufflinks) to detect DEGs between population pairs from distinct altitudes within each group of specific tissue and species. Genes with FDR-adjusted p-values ≤ 0.05 were detected as DEGs.

Genes were converted to human orthologs, and assessed by DAVID [17] webserver for functional enrichment in GO (Gene Ontology) terms consisting of molecular function (MF) and biological process (BP) as well as the KEGG pathways and InterPro databases (Benjamini adjusted p-values ≤ 0.05).

Analyses of alternative splicing

Single-copy orthologous exons were identified by finding annotated exons that overlapped with the query exonic region in a multiple alignment of 99 vertebrate genomes including human genome (hg38) from the UCSC genome browser [18]. Exon groups with multiple overlapping exons in any species were excluded. Each internal exon in every annotated transcript was taken as an “cassette” exon. Each “cassett” alternative splicing (AS) is composed of three exons: C1, A and C2, where A is alternative exon, C1 the 5’ alternative exon, C2 the 3’ alternative exon. For each species and read length k, we generated all non-redundant constitutive and alternative junction sequences for the

following RNA-seq alignments. The junction sequences were constructed by retrieving k-8 bp from each of the two exons making up the junction, and when the exon length is smaller than k-8, the whole sequence of the exon is retrieved. This ensures that there is at least 8 bp overlap between the mapped reads and each of the two junction exons.

We then estimated the effective number of uniquely mappable positions of the junctions. We extracted L-k+1 (L being the junction length) k-mers from each junction and mapped such k-mers back to the reference genome allowing up to two mismatches. Those k-mers that failed to align were further mapped to the non-redundant junctions. The number of k-mers that could uniquely align to a junction was counted and deemed as the effective number of uniquely mappable positions for the junction.

For each sample, RNA-seq reads were first aligned to the reference genome allowing up to two mismatches, and the unaligned reads were further mapped to the non-redundant junctions. Uniquely mapped reads for each junction were counted, and multiplied by the ratio between the maximum number of mappable positions (i.e. k-15) to the effective number of uniquely mappable positions for the junction.

The “percent-spliced in” (PSI) values for each internal exon was defined as $PSI = 100 \times \text{average}(\#C1A, \#AC2) / (\#C1C2 + \text{average}(\#C1A, \#AC2))$, here #C1A, #AC2 and #C1C2 are the normalized read counts for the associated junctions. Exons were taken as alternative in a sample if $5 \le PSI \le 95$. We also defined “high-confidence” PSI levels as those that meet the following criteria:

*$\max(\min(\#C1A, \#AC2), \#C1C2) \ge 5$ AND $\min(\#C1A, \#AC2) + \#C1C2 \ge 10$

*$|\log2(\#C1A / \#AC2)| \le 1$ OR $\max(\#C1A, \#AC2) < \#C1C2$

For cross-species analyses, we included exons with single-copy orthologues in all species, PSI values in all samples, and confidently alternative spliced in at least one of the samples.

Findings

Data summary

We generated a total of ~909.6 Gb high-quality RNA-seq data, of which ~676.6 Gb (~74.6%) reads could reliably aligned to their respective reference genomes **(Supplementary Fig. S3** and **Table S1)**. We found that on average 61.2% annotated protein coding genes in each genome had FPKM expression values greater than 0.5 **(Supplementary Fig. S4** and **Table S3)**.

Concordance in the tree topology based on nucleotide sequence alignments and gene expression data

Nucleotide alignments-based phylogenetic relationships of these high-altitude vertebrates and their low-altitude relatives matched the established morphological species groupings and the known history of population formation **(Fig. 1b)**. The gene expression-based tree based 4,746 transcribed single-copy orthologous genes (66.61% of 7125) for each tissue showed a highly consistent topology to the nucleotide sequence alignment-based phylogeny **(Fig. 2, Supplementary Methods) [9]**: mammals were mainly divided into omnivore (pig) and ruminant (goat, sheep and yak/cattle); within the ruminant cluster, the two caprinae (goat and sheep) were closer to each other than the bovinae (yak/cattle). This observation lends supports the idea that gene expression

changes evolve together with genetic variation over evolutionary time, resulting in lower expression divergence between more closely species [19].

Distinctly transcriptomic characteristics between gene expression and alternative splicing

Through comparison of expression levels of 4,746 transcribed single-copy orthologous genes **(Supplementary Fig. S2)** and alternative splicing patterns (reflected by PSI values) of 2,783 orthologous exons shared by the five vertebrates genomes, we observed a tissue-dominated clustering pattern of gene expression, but a species-dominated clustering pattern of alternative splicing [20, 21].

For gene expression, there were critical biological differences among tissues (Pearson's *r* = 0.67 and weighted average proportion variance = 0.36), followed by species (Pearson's *r* = 0.75, weighted average proportion variance = 0.22) and local adaptation (Pearson's *r* = 0.95 and weighted average proportion variance = 0.019) **(Fig. 3a** and **Supplementary Fig. S5)**. By contrast, for alternative splicing, the differences among species (Pearson's *r* = 0.64 and weighted average proportion variance = 0.30) were higher than among tissues (Pearson's *r* = 0.78 and weighted average proportion variance = 0.075), followed by between high- and low-altitude animals (Pearson's *r* = 0.84 and weighted average proportion variance = 0.021) **(Fig. 3b** and **Supplementary Figure S6)**.

Unsupervised clustering **(Figs. 4a and 4c)** and principal components analysis (PCA) **(Figs. 4b and 4d** and **Supplementary Figs. S7 and S8)** both recapitulated the distinctly transcriptomic characteristics between gene expression and alternative splicing. Tissue-dominated clustering of gene

expression indicated that in general tissues possess conserved gene expression signatures and suggested that conserved gene expression differences underlie tissue identity in mammals. On the other hand, greater prominence of species-dominated clustering of alternative splicing suggested that exon splicing is more often affected by species-specific changes in *cis*-regulatory elements and/or *trans*-acting factors than gene expression [20, 21].

Notably, tissue-dominated clustering patterns of gene expression further revealed that the cluster of striated muscle (heart and skeletal muscle) and the cluster of vessel-rich tissues (lung and spleen) were closer to each other than the cluster of metabolic tissues (kidney and liver), followed by the distinct clusters of bird (chicken) and mammals according to the evolutionary distance **(Figs. 4a and 4b)**. Notably, tissues of birds (chickens) formed a distinct cluster, rather than with their mammalian counterparts, which indicates that divergence in gene expression among these species started to surpass that between different tissues around when birds diverged from mammals (approximately 300 million years ago) **(Figs. 4a and 4b)**.

Gene expression plasticity to a high-altitude environment

To exclude the impact of prominence of tissues-dominated clustering of gene expression, so as to comprehensively present transcriptomic differences involved in high-altitude response based on whole annotated genes of their respective genome assembly instead of the single-copy orthologous, we measured the pairwise difference of gene expression between the high-altitude populations and their low-altitude relatives within each tissue for each vertebrate.

We identified ~1,423 DEGs between 30 low- versus high-altitude pairs (177 DEGs in muscle of chickens to 3,853 DEGs in kidney of sheep) (**Table 1**). Notably, among five pairs of vertebrate, the highly-diverged yak and cattle [1] exhibited the highest number of DEG (~2,005) across six tissues. Among six tissues, the highly aerobic kidney [22] exhibited the highest number of DEGs (~2,097) across five pairs of vertebrates.

Expectedly, the more closely related vertebrates (**Fig. 1**) shared more DE genes (**Supplementary Figs. S9–10** and **Additional File 3**). Compared with shared DE genes among mammals, especially between the two closely related members of Caprinae (goat and sheep), the birds (chickens) exhibited significantly fewer shared DE genes with mammals (Wilcoxon rank sum test, P<0.0021) (**Supplementary Fig. S11**). We also identified significantly enriched functional gene categories of DE genes (Chi-square test or Fisher's exact test, $P<1.03 \times 10^{-4}$), which were shared among multiple pairwise comparisons (**Supplementary Figs. S12–13** and **Additional File 4**), that were potentially related to the dramatic phenotypic changes shaped by high-altitude adaptation, such as response to hypoxia (typically, 'oxidation reduction', 'heme binding', 'oxygen binding' , 'oxygen transport' and 'oxygen transporter activity'), cardiovascular system ('angiogenesis' and 'positive regulation of angiogenesis'), the efficiency of biomass production in the resource-poor highland ('metabolic pathways', 'cholesterol biosynthetic process' and 'steroid metabolic process') as well as immune response ('responses of immune and defense') (**Additional file 2**).

Conclusions

High-altitude adaptive evolution of transcription, and the convergence and divergence of transcriptional alteration across species in response to high-altitude environments, is an important topic of broad interest to the general biology community. Here we provide a comprehensive comparative transcriptome landscape of expression and alternative splicing variation between low- and high-altitude populations across multiple species for distinct tissues. Our data serves a valuable resource for further study on gene regulatory changes to adaptive evolution of complex phenotypes.

Availability of supporting data

The RNA-seq data for 180 samples was deposited in the NCBI Gene Expression Omnibus (GEO) under accession numbers GSE93855, GSE77020 and GSE66242. The re-sequencing data for 7 individuals was deposited in the NCBI-sequence read archive (SRA) under accession number SRP096151.

All supplementary figures and tables are provided in Additional file.

Reviewer links:

GSE93855:

https://www.ncbi.nlm.nih.gov/geo/query/acc.cgi?token=irqtigkgvtatnqt&acc=GSE93855

GSE77020:

https://www.ncbi.nlm.nih.gov/geo/query/acc.cgi?token=kpolsqsothybrcv&acc=GSE77020 (GSM1617847-GSM1617849 and GSM2042608-GSM2042610 are duplicates and represent the same samples)

GSE66242:

https://www.ncbi.nlm.nih.gov/geo/query/acc.cgi?token=absxuuywtfyhncx&acc=GSE66242 (9 goat samples derived from individuals sampled at 2000m altitude were not included in this study)

Ethics statement

All studies involving animals were conducted according to Regulations for the Administration of Affairs Concerning Experimental Animals (Ministry of Science and Technology, China, revised in June 2004). All experimental procedures and sample collection methods in this study were approved by the Institutional Animal Care and Use Committee of the College of Animal Science and Technology of Sichuan Agricultural University, Sichuan, China, under permit No. DKY-B20121406. Animals were allowed free access to food and water under normal conditions, and were humanely sacrificed as necessary, to ameliorate suffering.

Consent for publication

Not applicable.

Competing interests

The authors declare that they have no competing interests.

Funding

This work was supported by grants from the National High Technology Research and Development Program of China (863 Program) (2013AA102502), the National Natural Science Foundation of China (31402046, 31522055, 31601918, 31530073 and 31472081), the Science & Technology Support

Program of Sichuan (2016NYZ0042), the Youth Science Fund of Sichuan (2017JQ0011), the China Postdoctoral Science Foundation (2015M572486), China Agriculture Research System (CARS-36), the Program for Innovative Research Team of Sichuan Province (2015TD0012), the Program for Pig Industry Technology System Innovation Team of Sichuan Province (SCCXTD-005), the Project of Sichuan Education Department (15ZA0008, 15ZA0003, 16ZA0025 and 16ZB0037), the National Program for Support of Top-notch Young Professionals and the Young Scholars of the Yangtze River.

Authors' contributions

MZ.L., QZ.T., YR.G. and XW.L. designed and supervised the project. JQ.G., TD.C., SL.H., Y.L., XM.Y., X.T., ZJ.Z., XH.C., DY.L., XL.L. and XB.L. collected the data, L.J., R.L., J.L., KR.L., SL.T., GS.W., JD.M., X.W., MM.M. and AA.J. generated the data. QZ.T. and MZ.L. performed the bioinformatics analyses. QZ.T. and MZ.L. wrote the manuscript. XM.Z. and VN.G. revised the manuscript.

References

1. Qiu Q, Zhang G, Ma T, Qian W, Wang J, Ye Z, et al. The yak genome and adaptation to life at high altitude. Nat Genet. 2012;44(8):946-9. doi:10.1038/ng.2343.
2. Li M, Tian S, Jin L, Zhou G, Li Y, Zhang Y, et al. Genomic analyses identify distinct patterns of selection in domesticated pigs and Tibetan wild boars. Nat Genet. 2013;45(12):1431-8. doi:10.1038/ng.2811.

3. Li M, Chen L, Tian S, Lin Y, Tang Q, Zhou X, et al. Comprehensive variation discovery and recovery of missing sequence in the pig genome using multiple de novo assemblies. Genome Res. 2017 May;27(5):865-874. doi:10.1101/gr.207456.116.

4. International Chicken Genome Sequencing Consortium. Sequence and comparative analysis of the chicken genome provide unique perspectives on vertebrate evolution. Nature. 2004;432(7018):695-716. doi:10.1038/nature03154.

5. Groenen MA, Archibald AL, Uenishi H, Tuggle CK, Takeuchi Y, Rothschild MF, et al. Analyses of pig genomes provide insight into porcine demography and evolution. Nature. 2012;491(7424):393-8. doi:10.1038/nature11622.

6. Bovine Genome Sequencing and Analysis Consortium, Elsik CG, Tellam RL, Worley KC, Gibbs RA, et al. The genome sequence of taurine cattle: a window to ruminant biology and evolution. Science. 2009;324(5926):522-8. doi:10.1126/science.1169588.

7. Dong Y, Xie M, Jiang Y, Xiao N, Du X, Zhang W, et al. Sequencing and automated whole-genome optical mapping of the genome of a domestic goat (Capra hircus). Nat Biotechnol. 2013;31(2):135-41. doi:10.1038/nbt.2478.

8. Jiang Y, Xie M, Chen W, Talbot R, Maddox JF, Faraut T, et al. The sheep genome illuminates biology of the rumen and lipid metabolism. Science. 2014;344 (6188):1168-73. doi:10.1126/science.1252806.

9. Vilella AJ, Severin J, Ureta-Vidal A, Heng L, Durbin R and Birney E. EnsemblCompara GeneTrees: Complete, duplication-aware phylogenetic trees in vertebrates. Genome Res. 2009;19(2):327-35. doi:10.1101/gr.073585.107.

10. Li H and Durbin R. Fast and accurate long-read alignment with Burrows-Wheeler transform. Bioinformatics. 2010;26(5):589-95. doi:10.1093/bioinformatics/btp698.

11. McKenna A, Hanna M, Banks E, Sivachenko A, Cibulskis K, Kernytsky A, et al. The Genome Analysis Toolkit: a MapReduce framework for analyzing next-generation DNA sequencing data. Genome Res. 2010;20(9):1297-303.

doi:10.1101/gr.107524.110.

12. Li H, Coghlan A, Ruan J, Coin LJ, Heriche JK, Osmotherly L, et al. TreeFam: a curated database of phylogenetic trees of animal gene families. Nucleic Acids Res. 2006;34(Database issue):D572-80. doi:10.1093/nar/gkj118.

13. Trapnell C, Pachter L and Salzberg SL. TopHat: discovering splice junctions with RNA-Seq. Bioinformatics. 2009;25(9):1105-11. doi:10.1093/bioinformatics/btp120.

14. Trapnell C, Williams BA, Pertea G, Mortazavi A, Kwan G, van Baren MJ, et al. Transcript assembly and quantification by RNA-Seq reveals unannotated transcripts and isoform switching during cell differentiation. Nat Biotechnol. 2010;28(5):511-5. doi:10.1038/nbt.1621.

15. The pvca R package. https://bioconductor.org/packages/release/bioc/html/pvca.html. Accessed Feb 16 2017.

16. Paradis E, Claude J and Strimmer K. APE: Analyses of phylogenetics and evolution in R language. Bioinformatics. 2004;20(2):289-90.

17. Dennis G, Jr., Sherman BT, Hosack DA, Yang J, Gao W, Lane HC, et al. DAVID: Database for annotation, visualization, and integrated discovery. Genome Biol. 2003;4(5):P3.

18. Kent WJ, Sugnet CW, Furey TS, Roskin KM, Pringle TH, Zahler AM, et al. The human genome browser at UCSC. Genome Res. 2002;12(6):996-1006. doi:10.1101/gr.229102.

19. Brawand D, Soumillon M, Necsulea A, Julien P, Csardi G, Harrigan P, et al. The evolution of gene expression levels in mammalian organs. Nature. 2011;478 (7369):343-8. doi:10.1038/nature10532.

20. Merkin J, Russell C, Chen P and Burge CB. Evolutionary dynamics of gene and isoform regulation in Mammalian tissues. Science. 2012;338(6114):1593-9. doi:10.1126/science.1228186.

21. Barbosa-Morais NL, Irimia M, Pan Q, Xiong HY, Gueroussov S, Lee LJ, et al. The

evolutionary landscape of alternative splicing in vertebrate species. Science. 2012;338(6114):1587-93. doi:10.1126/science.1230612.

22. Mele M, Ferreira PG, Reverter F, DeLuca DS, Monlong J, Sammeth M, et al. Human genomics. The human transcriptome across tissues and individuals. Science. 2015;348(6235):660-5. doi:10.1126/science.aaa0355.

Figures 1-4

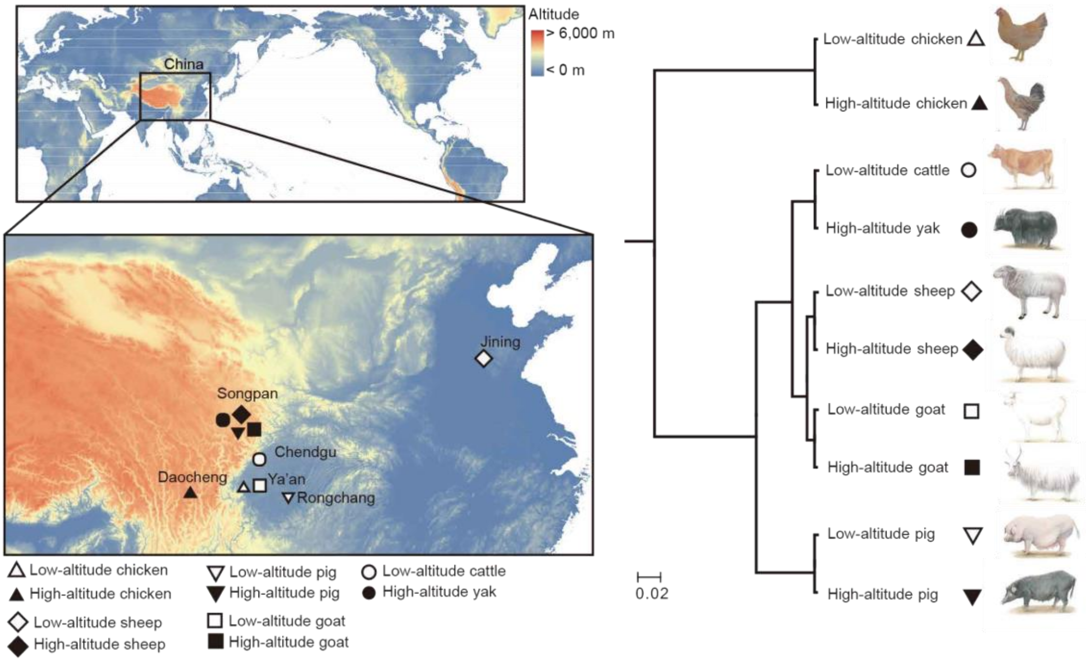

Figure 1. Sampling locations and nucleotide alignment-based tree.

(a) Geographic locations of the studied animals.

(b) A neighbour-joining tree constructed based on concatenated coding sequences of single-copy orthologues substituted by SNVs and InDels detected in each animal.

We downloaded and extracted the unassembled reads from short-insert (500 bp) libraries of a single yak [1], a Tibetan pig [2] and a Rongchang pig [3] that were used for *de novo* assemblies to roughly 10 × depth coverage. We also randomly selected an individual of the cattle, low- and high-altitude chicken, goat and sheep and sequenced the whole genomes at ~10 × depth coverage.

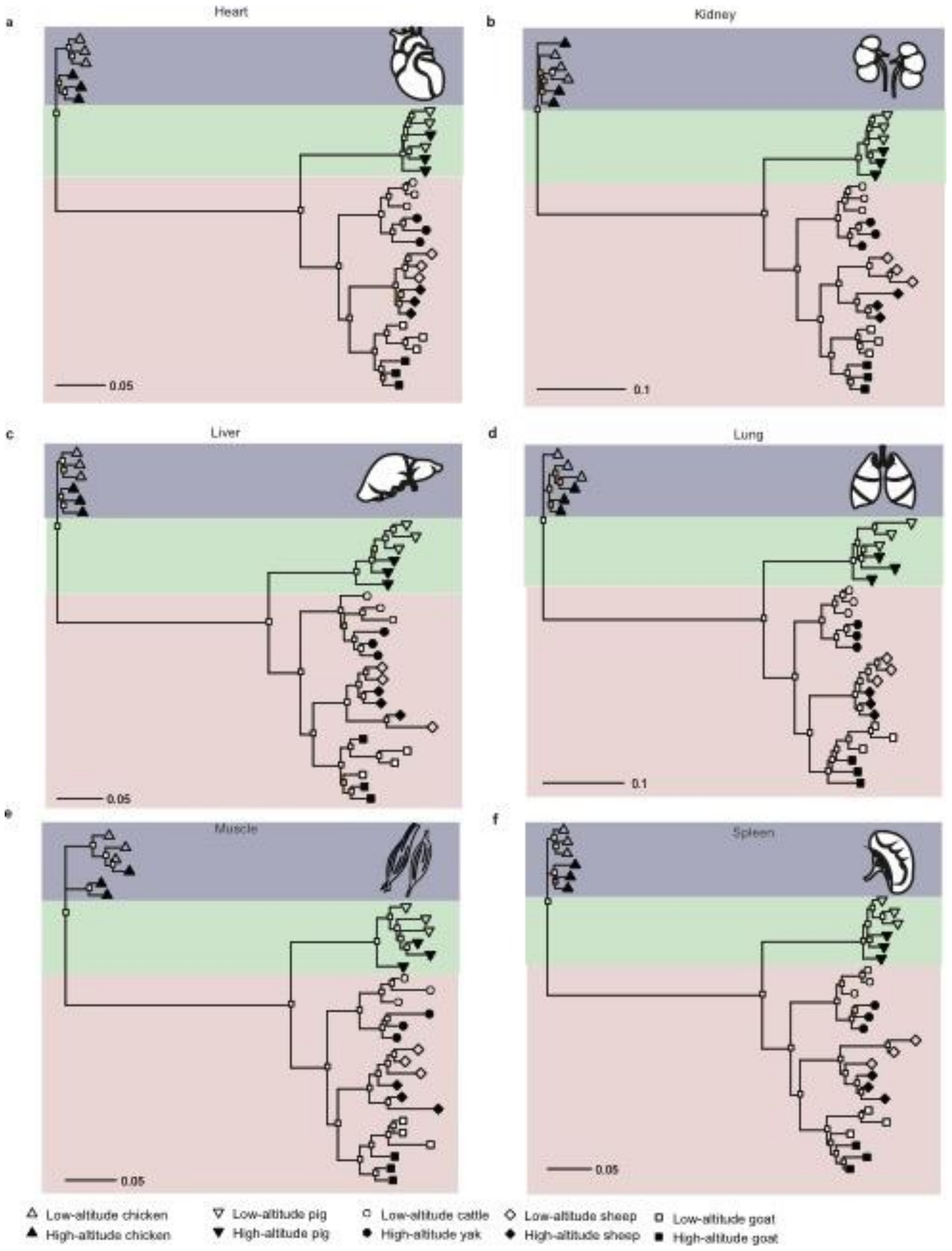

Figure 2. Gene expression phylogenies for six tissues across five vertebrates. Neighbour-joining expression tree constructed based on (1-Spearman correlation) distances in six tissues. We performed 100 bootstraps by randomly sampling the single copy orthologues with replacement. Bootstrap values (fractions of replicate trees that have the branching pattern as in the shown tree constructed using all the transcribed single copy orthologues) are indicated by different colors: red color of the node indicates support from

less than 50% bootstraps, while orange, yellow and white colors indicate support between 50% and 70%, between 70% and 90% and more than 90%, respectively.

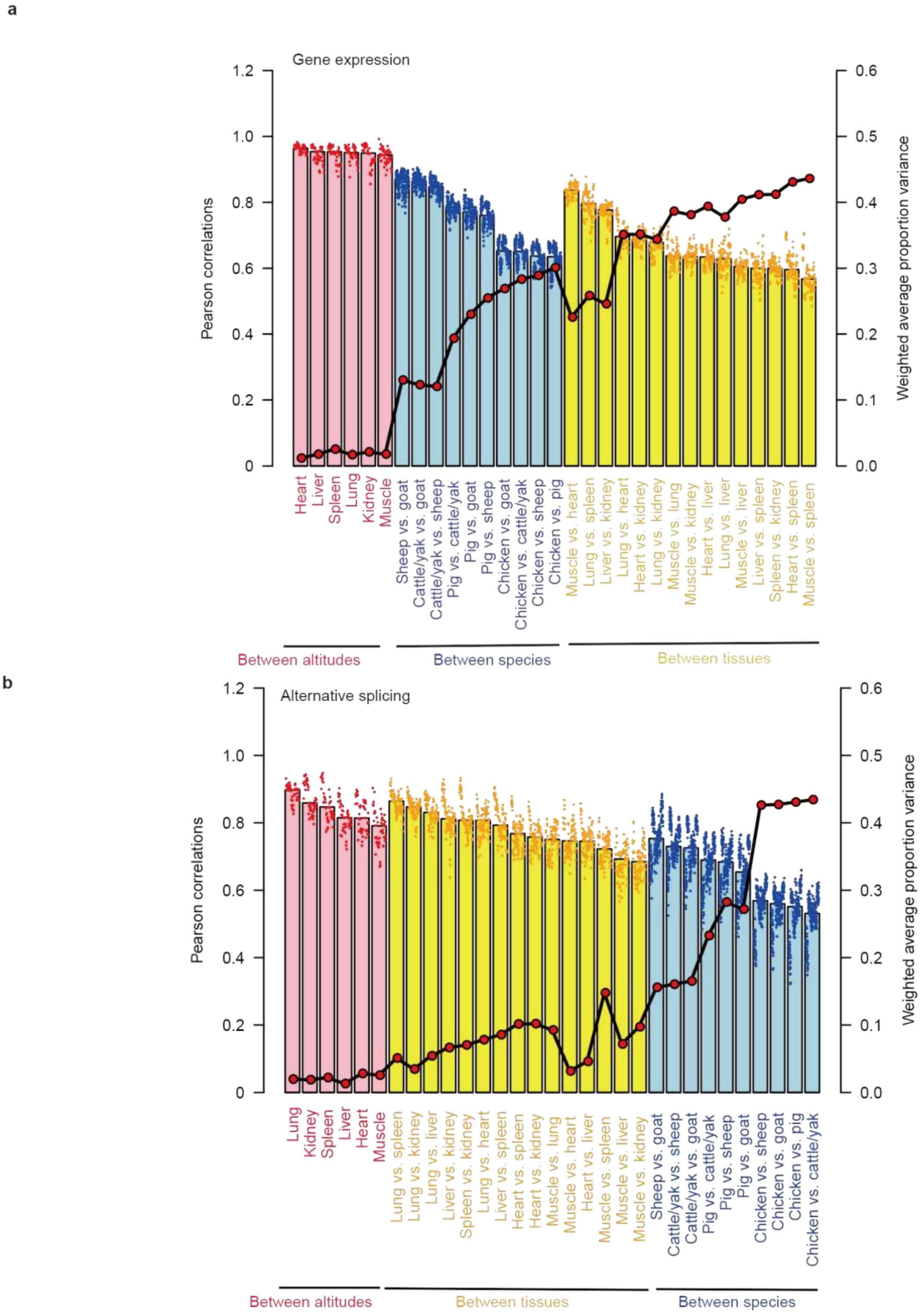

Figure 3. Comparison of variations between altitudes, species and tissues revealed by (a) gene expression and (b) alternative splicing pattern.

Scatter-point and bar plots represent the pairwise Pearson's correlation between samples. Weighted average proportion variance of the alternative splicing (reflected by PSI values) were determined using the Principal Variance Component Analysis (PVCA) approach and depicted as red dots connected by black lines.

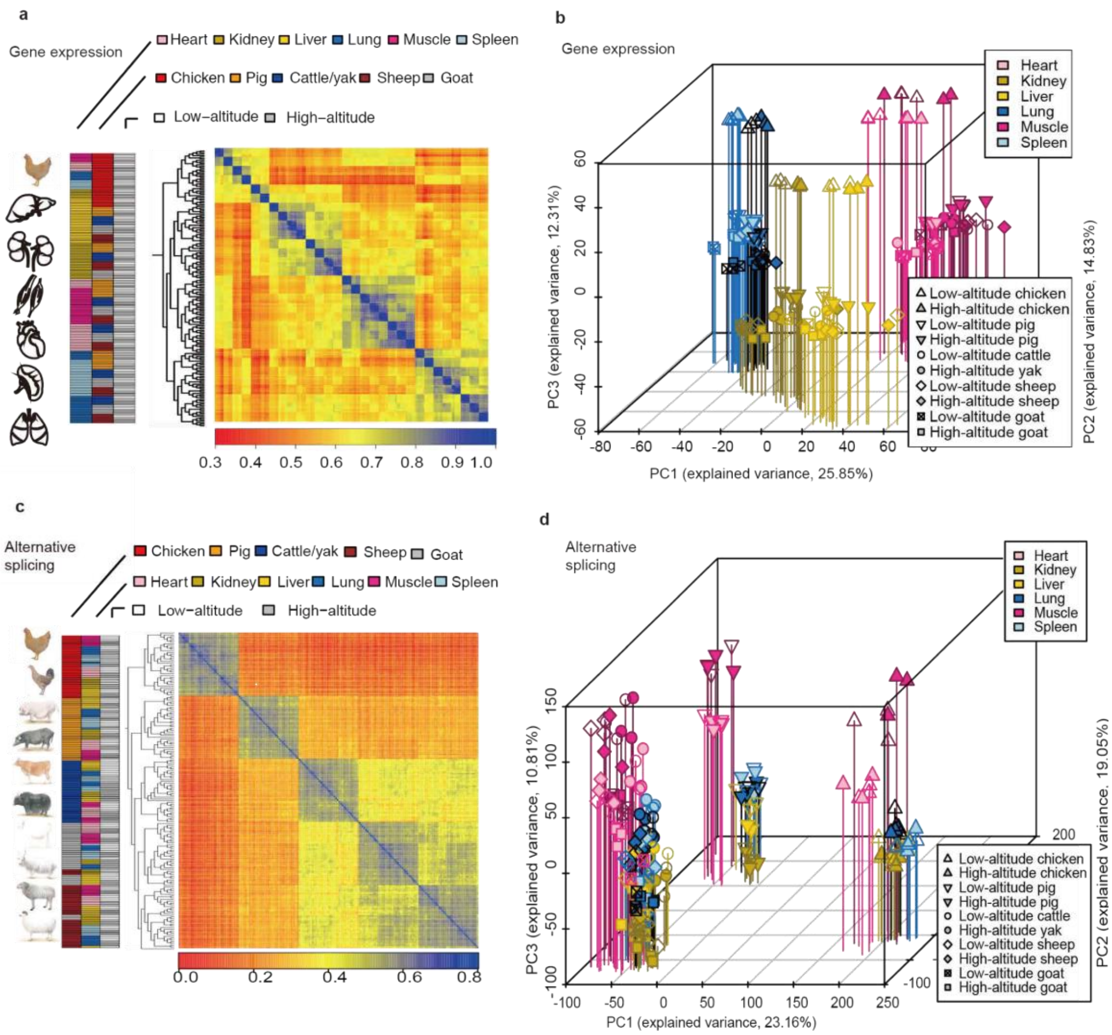

Figure 4. Global pattern of gene expression and alternative splicing pattern.

Hierarchical clustering of samples using **(a)** the gene expression and **(c)** the alternative splicing (reflected by PSI values). Average linkage hierarchical clustering was used with distance between samples measured by the Pearson's correlation between the vectors of expression values. Factorial map of the principal-component analysis (PCA) of **(b)** gene

expression levels and **(d)** the alternative splicing. The proportion of the variance explained by the principal components is indicated in parentheses.

Table 1. Number of DEGs between five high-altitude vertebrates and their low-altitude relatives for each tissue

Species	Heart	Kidney	Liver	Lung	Muscle	Spleen	Mean
**Chicken**	1283 (8.28%)	748 (4.83%)	613 (3.96%)	1072 (6.92%)	177 (1.14%)	984 (6.35%)	812 (5.25%)
**Pig**	206 (0.95%)	532 (2.46%)	1199 (5.55%)	426 (1.97%)	385 (1.78%)	994 (4.60%)	623 (2.89%)
**Cattle/yak**	1602 (8.02%)	1797 (8.99%)	869 (4.35%)	3092 (15.47%)	2403 (12.03%)	2268 (11.35%)	2005 (10.04%)
**Sheep**	1332 (6.37%)	3853 (18.43%)	259 (1.24%)	1829 (8.75%)	1079 (5.16%)	2356 (11.27%)	1784 (8.54%)
**Goat**	2215 (10.01%)	3557 (16.07%)	655 (2.96%)	1330 (6.01%)	2305 (10.42%)	1269 (5.73%)	1888 (8.53%)
**Mean**	1327 (6.73%)	2097 (10.16%)	719 (3.61%)	1549 (7.82%)	1269 (6.11%)	1574 (7.86%)	

Percentage of the DGEs compared with the total number of annotated protein coding genes in their respective reference genomes are listed in parenthesis. There are 15495, 21594, 19981, 22131, 20908 annotated protein coding genes in reference genomes of Chicken (Galgal4) [4], pig (Suscrofa 10.2) [5], cattle (UMD3.1) [6], goat (CHIR_1.0) [7] and sheep (Oar_v3.1) [8], respectively.

Table 1. Number of DEGs between five high-altitude vertebrates and their low-altitude relatives for each tissue

Species	Heart	Kidney	Liver	Lung	Muscle	Spleen	Mean
**Chicken**	1283 (8.28%)	748 (4.83%)	613 (3.96%)	1072 (6.92%)	177 (1.14%)	984 (6.35%)	812 (5.25%)
**Pig**	206 (0.95%)	532 (2.46%)	1199 (5.55%)	426 (1.97%)	385 (1.78%)	994 (4.60%)	623 (2.89%)
**Cattle/yak**	1602 (8.02%)	1797 (8.99%)	869 (4.35%)	3092 (15.47%)	2403 (12.03%)	2268 (11.35%)	2005 (10.04%)
**Sheep**	1332 (6.37%)	3853 (18.43%)	259 (1.24%)	1829 (8.75%)	1079 (5.16%)	2356 (11.27%)	1784 (8.54%)
**Goat**	2215 (10.01%)	3557 (16.07%)	655 (2.96%)	1330 (6.01%)	2305 (10.42%)	1269 (5.73%)	1888 (8.53%)
**Mean**	1327 (6.73%)	2097 (10.16%)	719 (3.61%)	1549 (7.82%)	1269 (6.11%)	1574 (7.86%)	

Percentage of the DGEs compared with the total number of annotated protein coding genes in their respective reference genomes are listed in parenthesis. There are 15495, 21594, 19981, 22131, 20908 annotated protein coding genes in reference genomes of Chicken (Galgal4) [4], pig (Suscrofa 10.2) [5], cattle (UMD3.1) [6], goat (CHIR_1.0) [7] and sheep (Oar_v3.1) [8], respectively.

Figure1

Click here to download Figure Figure1.pdf

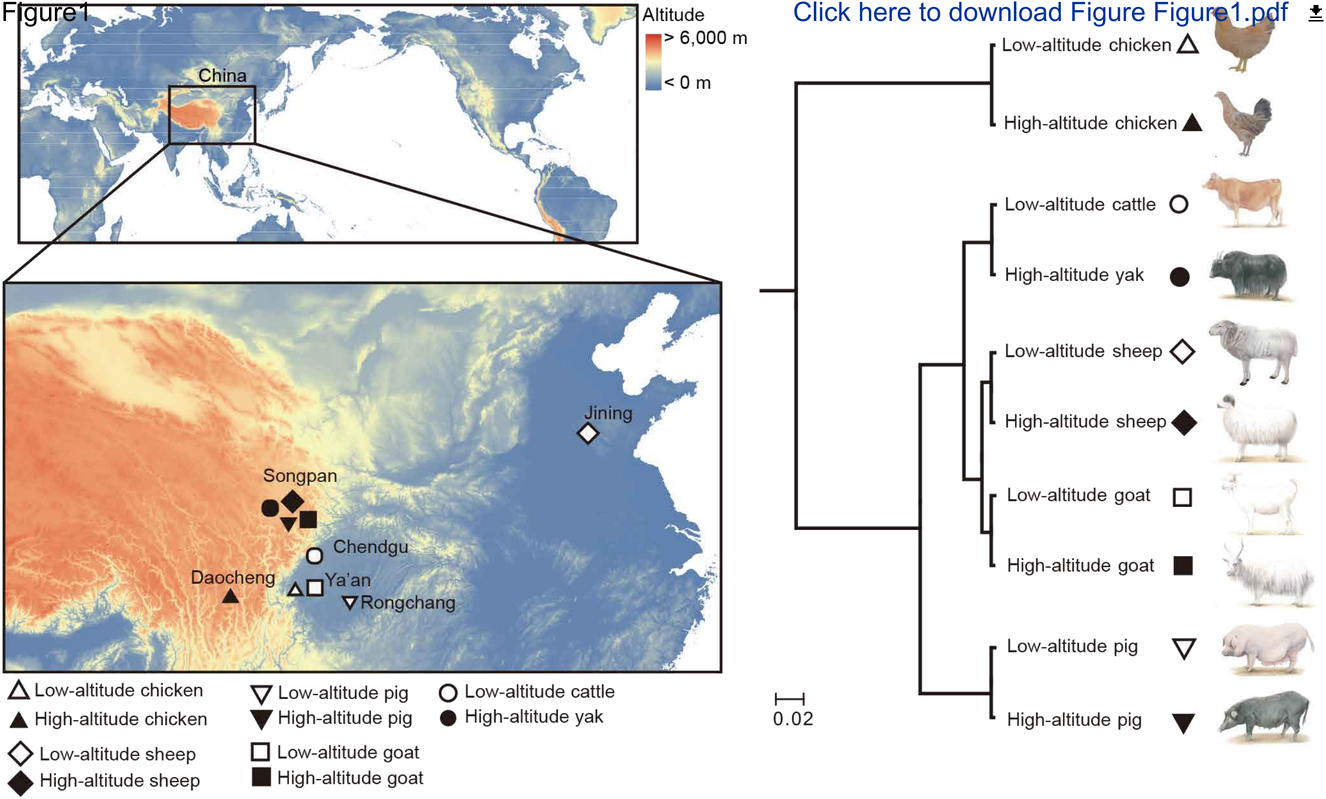

Figure2

Click here to download Figure Figure2.pdf

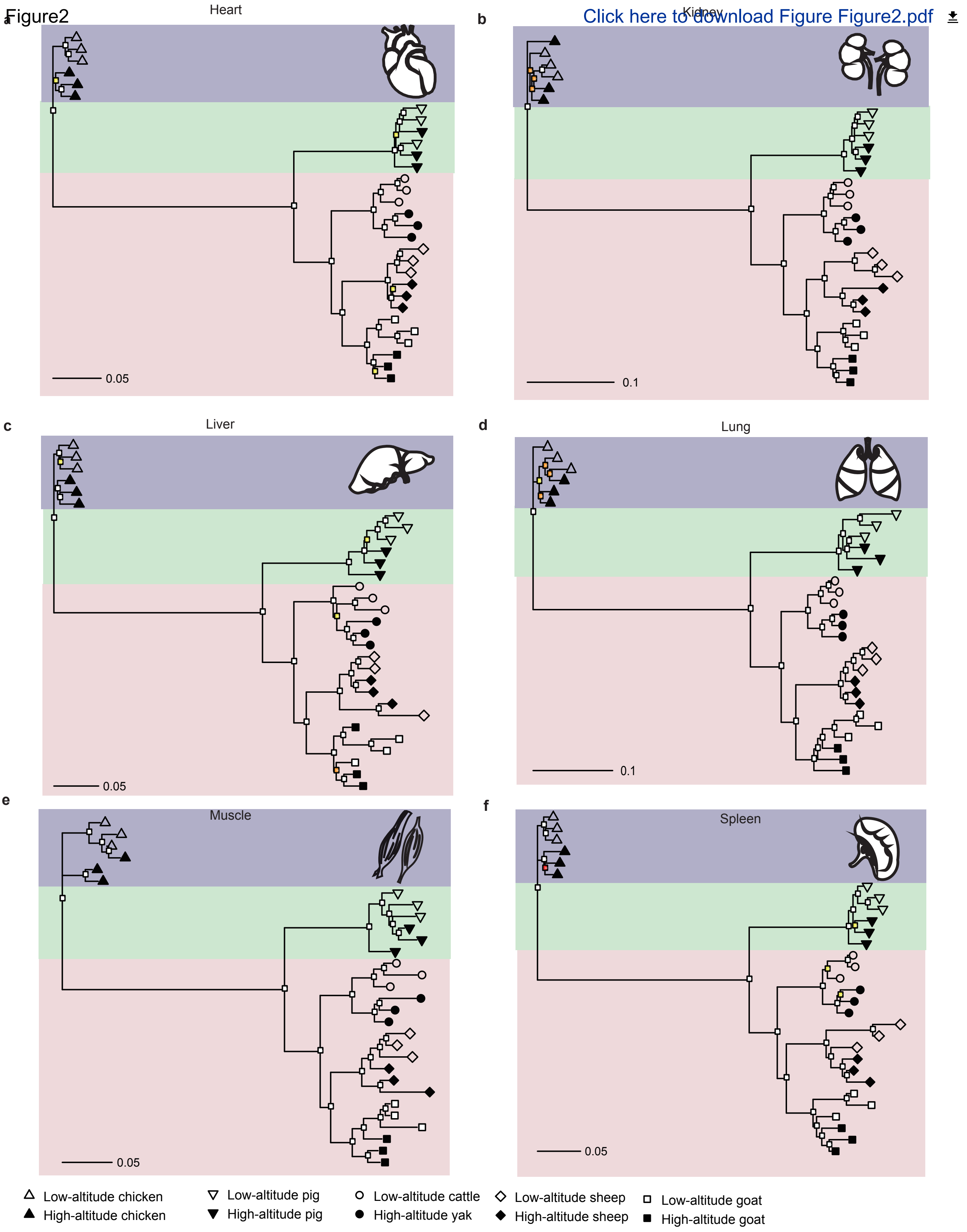

Figure3

Click here to download Figure Figure3.pdf

a

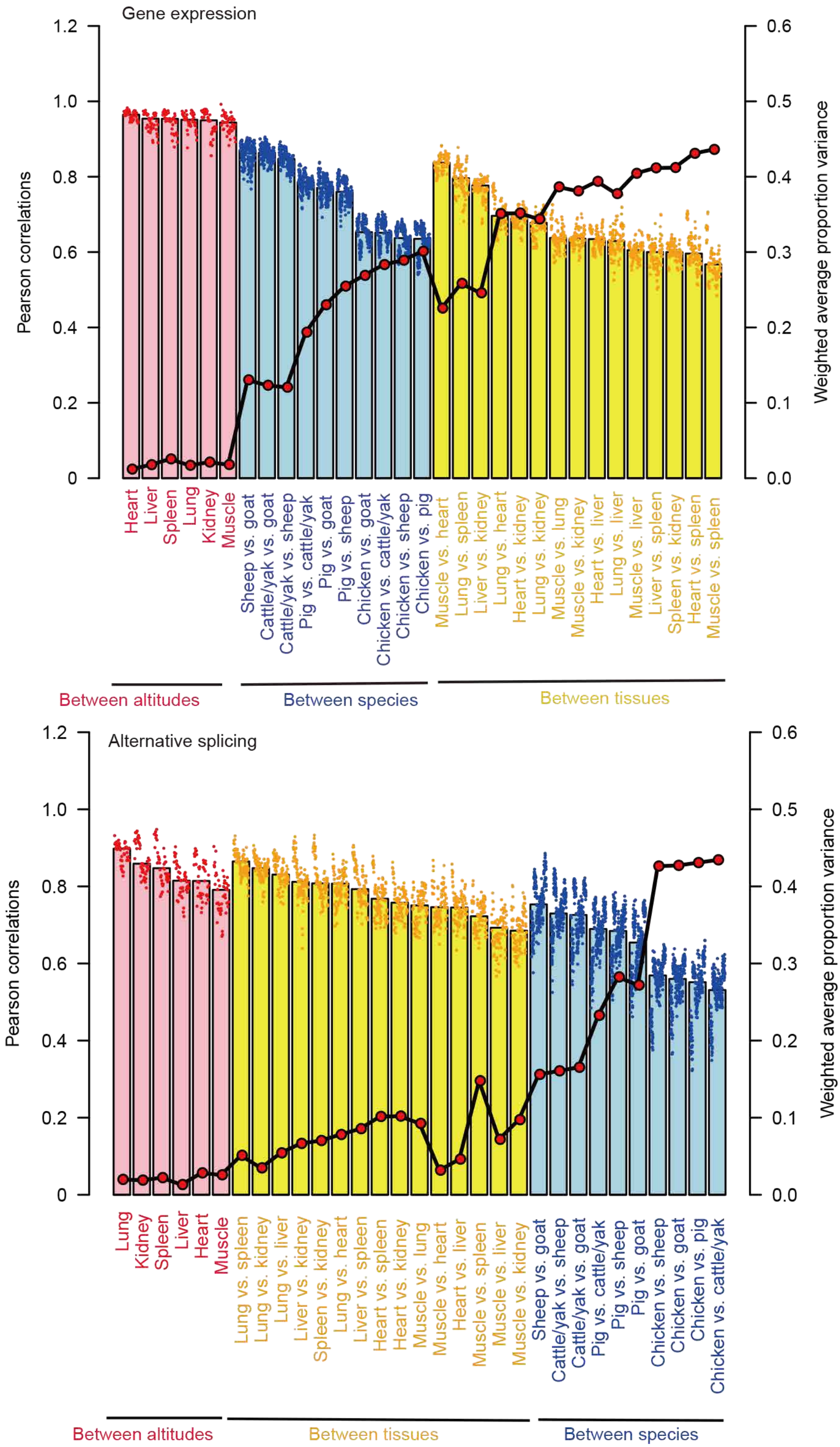

b

Figure4

Click here to download Figure Figure4.pdf

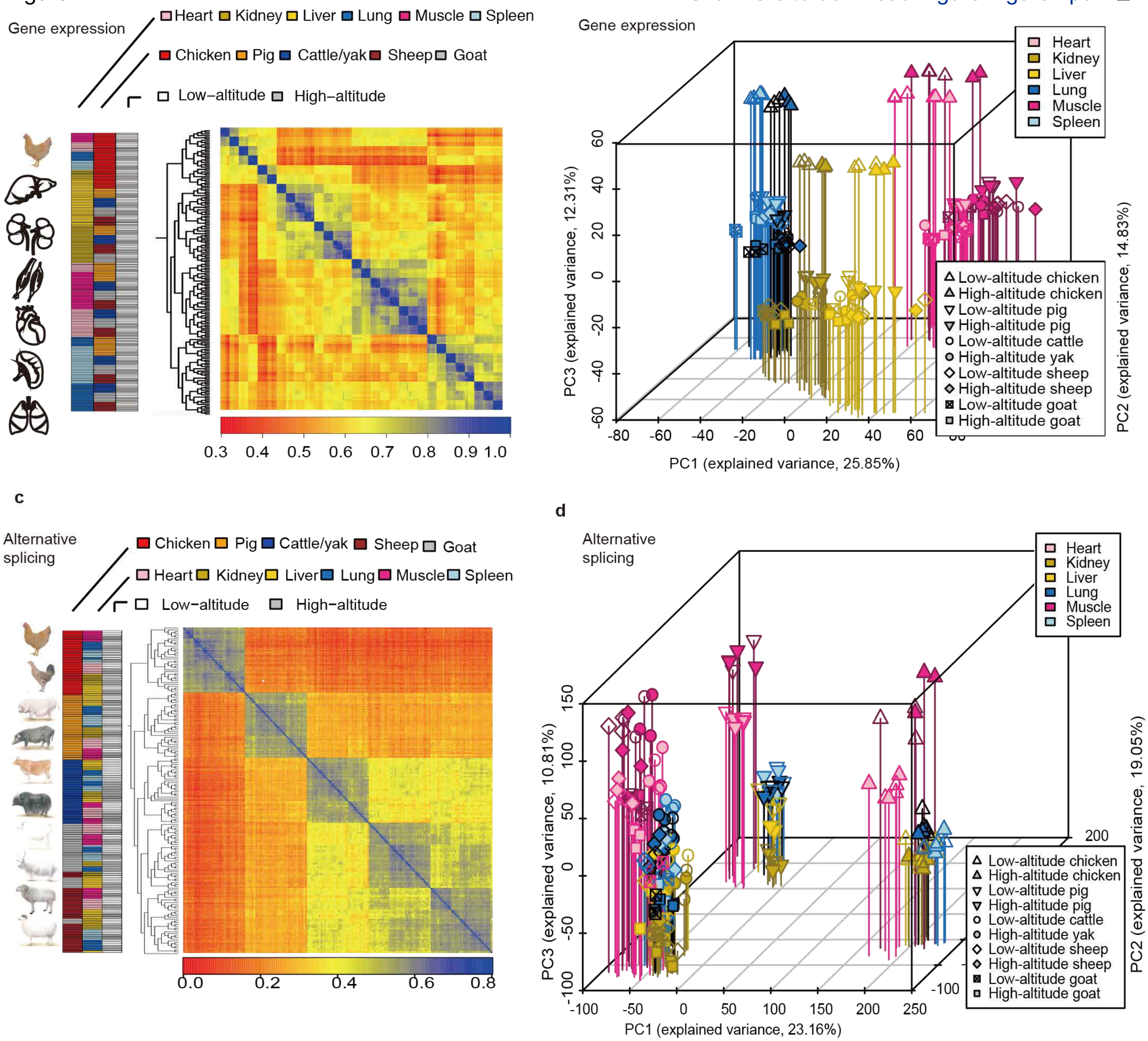

Click here to access/download
Supplementary Material
Additional_File_2.xlsx

Additional_File_3

Click here to access/download
Supplementary Material
Additional_File_3.xlsx

Click here to access/download
Supplementary Material
Additional_File_4.xlsx

RL_FiguresandTables

Click here to access/download
Supplementary Material
RL_FiguresandTables.pdf

Additional_File_1

Click here to access/download
Supplementary Material
Additional_File_1.pdf

GigaScience

em@editorialmanager.com

Dear Dr. Hans Zauner,

We are delighted to be informed of the positive responses from you. Thank you for your consideration of our manuscript for publication in *GigaScience.* We sincerely appreciate the thoughtful and constructive comments from the two reviewers Dr. Christopher Tuggle and Dr. Andreia Amaral, and your assistance in improving the manuscript. We have gone through your as well as the reviewers' comments in detail and believe that we have fully addressed these questions and concerns. We substantially improved our manuscript, and added 5 supplementary figures comprising 30 panels and 2 additional files. Below we provide our point-to-point responses, and hope that they are satisfactory.

We look forward to hearing a positive response from you.

Best regards,

Dr. Mingzhou Li

Sichuan Agricultural University, Chengdu, Sichuan, China

Email: mingzhou.li@sicau.edu.cn

Detailed responses to editor

All comments provided by editor are in gray italics, and our responses are in black. Important revisions in the manuscript are marked in red.

Editor: Dr. Hans Zauner

Comment 1-1:

Reviewer 1 points out that you should share all of your gene lists - I should clarify this point, as it may be confusing without explanation: at the time of writing their report, the reviewer did not have access to your additional files, and in fact you already provide a lot of this material, as the reviewer also has confirmed in further correspondence, after inspecting the files. However, please make sure the gene lists and other supporting data are in fact complete.

Response 1-1:

Thank you for your reminder. According to the submission guidelines of *GigaScience*, we uploaded the complete gene lists with normalized expression values to the *GigaScience* temporary FTP server.

Comment 1-2:

Please also add the additional information on your methods and analyses the reviewers are asking for. Reviewer 2 recommends some additional analyses, that I feel will be a useful addition.

Response 1-2:

Thank you for your comments. Based on reviewer 2's valuable recommendations, we carried out two additionally explorative analyses.

First, to investigate the similarities of the gene expression pairwise differences between the high- and low-altitude populations, we identified

shared differentially expressed (DE) genes and common functional categories enriched by DE genes in the pairwise comparisons of each tissue for each vertebrate. We added **Supplementary Figs. 9–13** and **Additional Files 3–4** in the revised manuscript (see **Responses 2–3** and **3–9**, respectively, for details).

Second, to explore the potential impact of positive selection on gene transcription, we identified the genes embedded in the selected regions based on publicly available whole-genome sequence data of three vertebrates (i.e., chickens, pigs, and cattle that live at low altitudes) and their high-altitude relatives, and compared these genes and the changes at the transcriptomic level (see **Response 3–7**, **Figs. R1–4**, and **Table R2** for details, **Figs. R1–4**, and **Table R2** can be accessed from RL_FiguresandTables.pdf at: https://www.dropbox.com/s/shgpb4784s409zw/RL_FiguresandTables.pdf?dl=0).

Comment 1-3:

Please also make sure you follow all of the MNSEQE standards of reporting: http://www.fged.org/site_media/pdf/MINSEQE_1.0.pdf

Response 1-3:

Thank you for your kind reminder. According to your notification, we checked the data styles to completely conform to the MNSEQE standards of reporting.
