# Supplementary material for: Comparative transcriptomics of 5 high-altitude vertebrates and their low-altitude relatives
Source: Gigascience. 2017 Nov 15;6(12):1–9. doi: 10.1093/gigascience/gix105 (PMC5729692; doi:10.1093/gigascience/gix105)
Supplement: GIGA-D-17-00037_Revision-2.pdf [file gix105_giga-d-17-00037_revision-2.pdf]

GigaScience

Comparative transcriptomics of five high-altitude vertebrates and their low-altitude relatives

--Manuscript Draft--

Manuscript Number:	GIGA-D-17-00037R2
Full Title:	Comparative transcriptomics of five high-altitude vertebrates and their low-altitude relatives
Article Type:	Data Note
Funding Information:	National High Technology Research and Development Program of China (863 Program) (2013AA102502) — Prof. Mingzhou Li
	National Natural Science Foundation of China (31402046) — Dr Qianzi Tang
	National Natural Science Foundation of China (31522055) — Prof. Mingzhou Li
	National Natural Science Foundation of China (31601918) — Dr Jideng Ma
	National Natural Science Foundation of China (31530073) — Prof Xuewei Li
	National Natural Science Foundation of China (31472081) — Prof. Mingzhou Li
	Science & Technology Support Program of Sichuan (2016NYZ0042) — Dr Yiren Gu
	Youth Science Fund of Sichuan (2017JQ0011) — Dr Yiren Gu
	China Postdoctoral Science Foundation (2015M572486) — Dr Qianzi Tang
	China Agriculture Research System (CARS-36) — Dr Yiren Gu
	Program for Innovative Research Team of Sichuan Province (2015TD0012) — Prof. Mingzhou Li
	Program for Pig Industry Technology System Innovation Team of Sichuan Province (SCCXTD-005) — Dr Yiren Gu
	Project of Sichuan Education Department (15ZA0008) — Dr Xun Wang
	Project of Sichuan Education Department (15ZA0003) — Dr Miaomiao Mai
	Project of Sichuan Education Department (16ZA0025) — Dr Jideng Ma
	Project of Sichuan Education Department (16ZB0037) — Dr An'an Jiang
	National Program for Support of Top-notch Young Professionals — Prof. Mingzhou Li
	Young Scholars of the Yangtze River — Prof. Mingzhou Li
	National Natural Science Foundation of China (31772576) — Prof. Mingzhou Li
Abstract:	Background: Species living at high altitude are subject to strong selective pressures due to inhospitable environments (e.g., hypoxia, low temperature, high solar radiation, and lack of biological production), making these species valuable models for comparative analyses of local adaptation. Studies that examined high-altitude

Powered by Editorial Manager® and ProduXion Manager® from Aries Systems Corporation

adaptation identified a vast array of rapidly evolving genes that characterize the dramatic phenotypic changes in high-altitude animals. However, how high-altitude environment shapes gene expression programs remains largely unknown.
Findings: We generated a total of 910 Gb high-quality RNA-seq data for 180 samples derived from six tissues of five agriculturally important high-altitude vertebrates (Tibetan chicken, Tibetan pig, Tibetan sheep, Tibetan goat and yak), and their cross-fertile relatives living in geographically neighboring low-altitude regions. Of these, ~75% reads could be aligned to their respective reference genomes, and on average ~70% of annotated protein coding genes in each organism showed FPKM expression values greater than 0.1. We observed a general concordance in topological relationships between the nucleotide alignments and gene expression-based trees. Tissue and species accounted for markedly more variance than altitude based on either the expression or the alternative splicing patterns. Cross-species clustering analyses showed a tissue-dominated pattern of gene expression, and a species-dominated pattern for alternative splicing. We also identified numerous differentially expressed genes were potentially involved in phenotypic divergence shaped by high-altitude adaptation.
Conclusions: This data serves as a valuable resource for examining the convergence and divergence of gene expression changes between species as they adapt or acclimatize to high-altitude environments.
Keywords: high-altitude vertebrates, comparative transcriptomics, gene expression, alternative splicing

**Corresponding Author:**	Mingzhou Li, Ph.D.<br>Sichuan Agricultural University<br>Chengdu, Sichuan CHINA
**Corresponding Author Secondary Information:**	
**Corresponding Author's Institution:**	Sichuan Agricultural University
**Corresponding Author's Secondary Institution:**	
**First Author:**	Qianzi Tang
**First Author Secondary Information:**	
**Order of Authors:**	Qianzi Tang
	Yiren Gu
	Xuming Zhou
	Long Jin
	Jiuqiang Guan
	Rui Liu
	Jing Li
	Keren Long
	Shilin Tian
	Tiandong Che
	Silu Hu
	Yan Liang
	Xuemei Yang
	Xuan Tao
	Zhijun Zhong
	Guosong Wang
	Xiaohui Chen

Powered by Editorial Manager® and ProduXion Manager® from Aries Systems Corporation

	Diyan Li
	Jideng Ma
	Xun Wang
	Miaomiao Mai
	An'an Jiang
	Xiaolin Luo
	Xuebin Lv
	Vadim N. Gladyshev
	Xuewei Li
	Mingzhou Li, Ph.D.
**Order of Authors Secondary Information:**	
**Response to Reviewers:**	Reviewer 1:<br><br>Comment 2-1<br>Your Series GSE93855 submission is labeled as unavailable until Jan 13 2020. Under the FAIR principles of publication in GigaScience, this must change if paper is accepted. Other datasets are also embargoed, far into 2018 or 2019.<br>Response 2-1<br>Thank you for your reminder. We have released all of datasets related to our manuscript, inducing a total of 180 RNA-seq data deposited in the NCBI Gene Expression Omnibus (GEO) under accession numbers GSE93855, GSE77020 and GSE66242, as well as the 7 whole-genome sequencing data deposited in the NCBI-sequence read archive (SRA) under accession number SRP096151.<br><br>GSE93855: https://www.ncbi.nlm.nih.gov/geo/query/acc.cgi?acc=GSE93855<br>GSE77020: https://www.ncbi.nlm.nih.gov/geo/query/acc.cgi?acc=GSE77020<br>GSE66242: https://www.ncbi.nlm.nih.gov/geo/query/acc.cgi?acc=GSE66242<br>SRP096151: https://trace.ncbi.nlm.nih.gov/Traces/sra/?study=SRP096151<br><br>Comment 2-2<br>The additional work performed to answer my reviewer comments is very good and shows interesting results. I don't have further major concerns, other than fixing the data availability issue above.<br>Response 2-2<br>Thank you for your positive comments.<br><br>Comment 2-3<br>The authors could consider revising Figure 4b and 4d, which are quite complex and difficult to see the relationships of all datasets to each other. The use of different color lines was not explained, and I tried to see why these were used, but was unable to see the logic.<br>Response 2-3<br>Thank you for your valuable suggestion. In Fig. 4b and 4d, the vertical leading lines with different colors from the plotted points dropping to the xy-plane serve to improve the demonstration of group separation by tissue based on the first and second principal components. We added the explanation of the dropping vertical lines to the legends of Fig. 4b and 4d, “The vertical leading lines with different colors from the plotted points dropping to the xy-plane show the separation of points based on the first and second principal components”.<br>To further promote the clarity of depiction for the principal component analysis (PCA) results, we added the two-dimensional PCA figures as the Supplementary Figs. S14-15 (accessible from Response_sup.pdf at: https://www.dropbox.com/s/sivc1djpvqzty2s/Response_sup.pdf?dl=0)<br><br>Reviewer 2:<br><br>Comment 3-1

Powered by Editorial Manager® and ProduXion Manager® from Aries Systems Corporation

	Thank you for addressing the previous points raised during the first reviewing stage. I believe that most points have been addressed in an appropriate way, and once again I thank you for your work and the data generated.<br>Response 3-1<br>Thank you for your positive comments.
**Additional Information:**	
**Question**	**Response**
Are you submitting this manuscript to a special series or article collection?	No
**Experimental design and statistics**<br><br>Full details of the experimental design and statistical methods used should be given in the Methods section, as detailed in our Minimum Standards Reporting Checklist. Information essential to interpreting the data presented should be made available in the figure legends.<br><br>Have you included all the information requested in your manuscript?	Yes
**Resources**<br><br>A description of all resources used, including antibodies, cell lines, animals and software tools, with enough information to allow them to be uniquely identified, should be included in the Methods section. Authors are strongly encouraged to cite Research Resource Identifiers (RRIDs) for antibodies, model organisms and tools, where possible.<br><br>Have you included the information requested as detailed in our Minimum Standards Reporting Checklist?	Yes
**Availability of data and materials**<br><br>All datasets and code on which the conclusions of the paper rely must be either included in your submission or deposited in publicly available repositories (where available and ethically appropriate), referencing such data using a unique identifier in the references and in the “Availability of Data and Materials” section of your manuscript.<br><br>Have you have met the above requirement as detailed in our Minimum	Yes

Standards Reporting Checklist?	

Powered by Editorial Manager® and ProduXion Manager® from Aries Systems Corporation

Manuscript

Click here to download Manuscript Manuscriptnew_HighLowAlt_ED.docx

Comparative transcriptomics of five high-altitude vertebrates and their low-altitude relatives

Qianzi Tang[1†], Yiren Gu[2†*], Xuming Zhou[3†], Long Jin[1], Jiuqiang Guan[4], Rui Liu[1], Jing Li[1], Kereng Long[1], Shilin Tian[1], Tiandong Che[1], Silu Hu[1], Yan Liang[2], Xuemei Yang[2], Xuan Tao[2], Zhijun Zhong[2], Guosong Wang[1,5], Xiaohui Chen[2], Diyan Li[1], Jideng Ma[1], Xun Wang[1], Miaomiao Mai[1], An'an Jiang[1], Xiaolin Luo[4], Xuebin Lv[2], Vadim N. Gladyshev[3], Xuewei Li[1] and Mingzhou Li[1*]

[1] Institute of Animal Genetics and Breeding, College of Animal Science and Technology, Sichuan Agricultural University, Chengdu 611130, China;

[2] Animal Breeding and Genetics Key Laboratory of Sichuan Province, Pig Science Institute, Sichuan Animal Science Academy, Chengdu 610066, China

[3] Division of Genetics, Department of Medicine, Brigham and Women's Hospital, Harvard Medical School, Boston, Massachusetts, 02115 USA;

[4] Yak Research Institute, Sichuan Academy of Grassland Science, Chengdu 610097, China;

[5] Department of Animal Science, Texas A & M University, College Station, Texas, 77843 USA.

[†] These authors contributed equally to this work.

Corresponding authors. E-mail: Mingzhou Li: mingzhou.li@sicau.edu.cn, Yiren Gu: guyiren1128@163.com.

Abstract

Background: Species living at high altitude are subject to strong selective pressures due to inhospitable environments (e.g., hypoxia, low temperature, high solar radiation, and lack of biological production), making these species valuable models for comparative analyses of local adaptation. Studies that examined high-altitude adaptation identified a vast array of rapidly evolving genes that characterize the dramatic phenotypic changes in high-altitude animals. However, how high-altitude environment shapes gene expression programs remains largely unknown.

Findings: We generated a total of 910 Gb high-quality RNA-seq data for 180 samples derived from six tissues of five agriculturally important high-altitude vertebrates (Tibetan chicken, Tibetan pig, Tibetan sheep, Tibetan goat and yak), and their cross-fertile relatives living in geographically neighboring low-altitude regions. Of these, ~75% reads could be aligned to their respective reference genomes, and on average ~60% of annotated protein coding genes in each organism showed FPKM expression values greater than 0.5. We observed a general concordance in topological relationships between the nucleotide alignments and gene expression-based trees. Tissue and species accounted for markedly more variance than altitude based on either the expression or the alternative splicing patterns. Cross-species clustering analyses showed a tissue-dominated pattern of gene expression, and a species-dominated pattern for alternative splicing. We also identified numerous differentially expressed genes that could potentially be involved in phenotypic divergence shaped by

high-altitude adaptation.

Conclusions: This data serves as a valuable resource for examining the convergence and divergence of gene expression changes between species as they adapt or acclimatize to high-altitude environments.

Keywords: high-altitude vertebrates, comparative transcriptomics, gene expression, alternative splicing

Data description

Transcriptome sequencing

Six tissues (heart, kidney, liver, lung, skeletal muscle and spleen) of three unrelated adult females for each of five high-altitude vertebrates and their low-altitude relatives were sampled **(Fig. 1a** and **Supplementary Fig. S1)**. Animals were sacrificed humanely to ameliorate suffering. All animals and samples used in this study were collected according to the guidelines for the care and use of experimental animals established by the Ministry of Agriculture of China. We extracted total RNA, prepared libraries and sequenced the libraries on Illumina HiSeq 2000 or 2500 platforms. We generated a total of ~909.6 Gb high-quality RNA-seq data for 180 samples (~5.05 Gb per sample) of 30 individuals across 6 tissues **(Supplementary Table S1)**.

Whole-genome re-sequencing

To compare the phylogeny derived from gene expression with the phylogenetic relationships of the five high-altitude vertebrates and their low-altitude relatives, we constructed the phylogenetic tree based on nucleotide alignments. We extracted the unassembled reads from short-insert (500 bp) libraries of a single yak [1] (NCBI-SRA: SRX103159 to SRX103161, and

SRX103175 and SRX103176), a Tibetan pig [2] (NCBI-SRA: SRX219342) and a low-altitude Rongchang pig (NCBI-SRA: SRX1544519) [3] that were used for *de novo* assemblies to roughly 10 × depth coverage. We also randomly selected an individual of the cattle, low- and high-altitude chicken, goat and sheep, and sequenced their whole genomes at ~10 × depth coverage (NCBI-SRA: SRP096151). Genomic DNA was extracted from blood tissue of each individual. Sequencing was performed on the Illumina X Ten platform, and a total of 198.64 Gb of paired-end DNA sequence was generated **(Supplementary Table S2)**.

Data analysis

Data filtering

To avoid reads with artificial bias, we removed the following type of reads: (a) Reads with ≥ 10% unidentified nucleotides (N); (b) Reads with > 10 nt aligned to the adapter, allowing ≤ 10% mismatches; (c) Reads with > 50% bases having phred quality < 5.

Identification of single-copy orthologous genes

Single-copy orthologous genes across five reference genomes, i.e. chicken (Galgal4) [4], pig (Suscrofa 10.2) [5], cattle (UMD3.1) [6], goat (CHIR_1.0) [7] and sheep (Oar_v3.1) [8] were determined using a EnsemblCompara GeneTrees method [9] **(Supplementary Fig. S2, Supplementary Methods) [9].**

Construction of phylogenetic tree based on nucleotide alignments

High-quality re-sequencing data were mapped to their respective reference

genomes using BWA software, version 0.7.7 (BWA, RRID:SCR_010910) [10], reads with mapping quality > 0 were retained and potential PCR duplication cases were removed. For each individual, ~97.01% of reads were mapped to ~97.40% (at least 1 × depth coverage) or ~91.86% (at least 4 × depth coverage) of the reference genome assemblies **(Supplementary Table S2)**. Single nucleotide variations (SNVs) and insertion and deletions (InDels) were further detected by following GATK's best practice, version 3.3-0 (GATK, RRID:SCR_001876) [11]. We substituted SNVs and InDels identified in our study in the coding DNA sequences (CDS) of the respective reference genomes. Single copy orthologues with substituted CDS of the five vertebrates were applied to Treebest [12] and generating the neighbor-joining tree (Fig. 1b).

Analyses of gene expression

High-quality RNA-seq reads were mapped to their respective reference genomes using Tophat version 2.0.11 (TopHat, RRID:SCR_013035) [13]. Cufflinks version 2.2.1 (Cufflinks, RRID:SCR_014597) [14] was applied to quantify gene expression and obtain FPKM expression values. We generated abundance files by applying Cuffquant (part of Cufflinks) to read mapping results. Log_2-transformed values of (FPKM + 1) for genes with >0.5 FPKM in over 80% of the samples were used for subsequent analyses.

Pearson's correlations were calculated across six samples from low- and high-altitudes populations within each group of specific tissue and animals; among pairwise comparisons of five animals within each of the six tissues; and among pairwise comparisons of six tissues within each of the five animals. Principal Variance Component Analysis (PVCA) was carried out using R package pvca [15]. Neighbor-joining expression-based trees were generated

according to distance matrices composed of pairwise (1-Spearman's correlations) implemented in the R package ape [16]. Reproducibility of branching patterns was estimated by bootstrapping genes, that is, the single copy orthologues were randomly sampled with replacement 100 times. The fractions of replicate trees that share the branching patterns of the original tree constructed were marked by distinct node colors in the figure.

We generated abundance files by applying Cuffquant (part of Cufflinks) to read mapping results, and further applied abundance files to Cuffdiff (part of Cufflinks) to detect DEGs between population pairs from distinct altitudes within each group of specific tissue and species. Genes with FDR-adjusted p-values ≤ 0.05 were detected as DEGs.

Genes were converted to human orthologs, and assessed by DAVID (DAVID, RRID:SCR_001881) [17] webserver for functional enrichment in GO (Gene Ontology) terms consisting of molecular function (MF) and biological process (BP) as well as the KEGG (KEGG, RRID:SCR_012773) pathways and InterPro (InterPro, RRID:SCR_006695) databases (Benjamini adjusted p-values ≤ 0.05).

Analyses of alternative splicing

Single-copy orthologous exons were identified by finding annotated exons that overlapped with the query exonic region in a multiple alignment of 99 vertebrate genomes including human genome (hg38) from the UCSC genome browser [18]. Exon groups with multiple overlapping exons in any species were excluded. Each internal exon in every annotated transcript was taken as an

“cassette” exon. Each “cassett” alternative splicing (AS) is composed of three exons: C1, A and C2, where A is alternative exon, C1 the 5’ alternative exon, C2 the 3’ alternative exon. For each species and read length k, we generated all non-redundant constitutive and alternative junction sequences for the following RNA-seq alignments. The junction sequences were constructed by retrieving k-8 bp from each of the two exons making up the junction, and when the exon length is smaller than k-8, the whole sequence of the exon is retrieved. This ensures that there is at least 8 bp overlap between the mapped reads and each of the two junction exons.

We then estimated the effective number of uniquely mappable positions of the junctions. We extracted L-k+1 (L being the junction length) k-mers from each junction and mapped such k-mers back to the reference genome allowing up to two mismatches. Those k-mers that failed to align were further mapped to the non-redundant junctions. The number of k-mers that could uniquely align to a junction was counted and deemed as the effective number of uniquely mappable positions for the junction.

For each sample, RNA-seq reads were first aligned to the reference genome allowing up to two mismatches, and the unaligned reads were further mapped to the non-redundant junctions. Uniquely mapped reads for each junction were counted, and multiplied by the ratio between the maximum number of mappable positions (i.e. k-15) to the effective number of uniquely mappable positions for the junction.

The “percent-spliced in” (PSI) values for each internal exon was defined as PSI = 100 × average (#C1A, #AC2) / (#C1C2 + average(#C1A, #AC2)), here #C1A, #AC2 and #C1C2 are the normalized read counts for the associated

junctions. Exons were taken as alternative in a sample if 5≤PSI≤95. We also defined "high-confidence" PSI levels as those that meet the following criteria:

*max(min(#C1A, #AC2), #C1C2) ≥ 5 AND min(#C1A, #AC2) + #C1C2 ≥ 10

*|log2(#C1A / #AC2)| ≤ 1 OR max(#C1A, #AC2) < #C1C2

For cross-species analyses, we included exons with single-copy orthologues in all species, PSI values in all samples, and confidently alternative spliced in at least one of the samples.

Findings

Data summary

We generated a total of ~909.6 Gb high-quality RNA-seq data, of which ~676.6 Gb (~74.6%) reads could reliably aligned to their respective reference genomes **(Supplementary Fig. S3** and **Table S1)**. We found that on average 61.2% annotated protein coding genes in each genome had FPKM expression values greater than 0.5 **(Supplementary Fig. S4** and **Table S3)**.

Concordance in the tree topology based on nucleotide sequence alignments and gene expression data

Nucleotide alignments-based phylogenetic relationships of these high-altitude vertebrates and their low-altitude relatives matched the established morphological species groupings and the known history of population formation **(Fig. 1b)**. The gene expression-based tree based 4,746 transcribed single-copy orthologous genes (66.61% of 7125) for each tissue showed a highly consistent

topology to the nucleotide sequence alignment-based phylogeny **(Fig. 2, Supplementary Methods) [9]**: mammals were mainly divided into omnivore (pig) and ruminant (goat, sheep and yak/cattle); within the ruminant cluster, the two caprinae (goat and sheep) were closer to each other than the bovinae (yak/cattle). This observation lends supports the idea that gene expression changes evolve together with genetic variation over evolutionary time, resulting in lower expression divergence between more closely species [19].

Distinctly transcriptomic characteristics between gene expression and alternative splicing

Through comparison of expression levels of 4,746 transcribed single-copy orthologous genes **(Supplementary Fig. S2)** and alternative splicing patterns (reflected by PSI values) of 2,783 orthologous exons shared by the five vertebrates genomes, we observed a tissue-dominated clustering pattern of gene expression, but a species-dominated clustering pattern of alternative splicing [20, 21].

For gene expression, there were critical biological differences among tissues (Pearson's r = 0.67 and weighted average proportion variance = 0.36), followed by species (Pearson's r = 0.75, weighted average proportion variance = 0.22) and local adaptation (Pearson's r = 0.95 and weighted average proportion variance = 0.019) **(Fig. 3a** and **Supplementary Fig. S5)**. By contrast, for alternative splicing, the differences among species (Pearson's r = 0.64 and weighted average proportion variance = 0.30) were higher than among tissues (Pearson's r = 0.78 and weighted average proportion variance = 0.075), followed by between high- and low-altitude animals (Pearson's r = 0.84 and weighted average proportion variance = 0.021) **(Fig. 3b** and **Supplementary**

Figure S6).

Unsupervised clustering **(Figs. 4a and 4c)** and principal components analysis (PCA) **(Figs. 4b and 4d** and **Supplementary Figs. S7 and S8)** both recapitulated the distinctly transcriptomic characteristics between gene expression and alternative splicing. Tissue-dominated clustering of gene expression indicated that in general tissues possess conserved gene expression signatures and suggested that conserved gene expression differences underlie tissue identity in mammals. On the other hand, greater prominence of species-dominated clustering of alternative splicing suggested that exon splicing is more often affected by species-specific changes in *cis*-regulatory elements and/or *trans*-acting factors than gene expression [20, 21].

Notably, tissue-dominated clustering patterns of gene expression further revealed that the cluster of striated muscle (heart and skeletal muscle) and the cluster of vessel-rich tissues (lung and spleen) were closer to each other than the cluster of metabolic tissues (kidney and liver), followed by the distinct clusters of bird (chicken) and mammals according to the evolutionary distance **(Figs. 4a and 4b)**. Notably, tissues of birds (chickens) formed a distinct cluster, rather than with their mammalian counterparts, which indicates that divergence in gene expression among these species started to surpass that between different tissues around when birds diverged from mammals (approximately 300 million years ago) **(Figs. 4a and 4b)**.

Gene expression plasticity to a high-altitude environment

To exclude the impact of prominence of tissues-dominated clustering of gene expression, so as to comprehensively present transcriptomic differences

involved in high-altitude response based on whole annotated genes of their respective genome assembly instead of the single-copy orthologous, we measured the pairwise difference of gene expression between the high-altitude populations and their low-altitude relatives within each tissue for each vertebrate.

We identified ~1,423 DEGs between 30 low- versus high-altitude pairs (177 DEGs in muscle of chickens to 3,853 DEGs in kidney of sheep) (**Table 1**). Notably, among five pairs of vertebrate, the highly-diverged yak and cattle [1] exhibited the highest number of DEG (~2,005) across six tissues. Among six tissues, the highly aerobic kidney [22] exhibited the highest number of DEGs (~2,097) across five pairs of vertebrates.

Expectedly, the more closely related vertebrates (**Fig. 1**) shared more DE genes (**Supplementary Figs. S9–10** and **Additional File 3**). Compared with shared DE genes among mammals, especially between the two closely related members of Caprinae (goat and sheep), the birds (chickens) exhibited significantly fewer shared DE genes with mammals (Wilcoxon rank sum test, P<0.0021) (**Supplementary Fig. S11**). We also identified significantly enriched functional gene categories of DE genes (Chi-square test or Fisher's exact test, $P<1.03 \times 10^{-4}$), which were shared among multiple pairwise comparisons (**Supplementary Figs. S12–13** and **Additional File 4**), that were potentially related to the dramatic phenotypic changes shaped by high-altitude adaptation, such as response to hypoxia (typically, 'oxidation reduction', 'heme binding', 'oxygen binding' , 'oxygen transport' and 'oxygen transporter activity'), cardiovascular system ('angiogenesis' and 'positive regulation of angiogenesis'), the efficiency of biomass production in the resource-poor

highland (‘metabolic pathways’, ‘cholesterol biosynthetic process’ and ‘steroid metabolic process’) as well as immune response (‘responses of immune and defense’) (**Additional file 2**).

Conclusions

High-altitude adaptive evolution of transcription, and the convergence and divergence of transcriptional alteration across species in response to high-altitude environments, is an important topic of broad interest to the general biology community. Here we provide a comprehensive comparative transcriptome landscape of expression and alternative splicing variation between low- and high-altitude populations across multiple species for distinct tissues. Our data serves a valuable resource for further study on gene regulatory changes to adaptive evolution of complex phenotypes.

Availability of supporting data

The RNA-seq data for 180 samples was deposited in the NCBI Gene Expression Omnibus (GEO) under accession numbers GSE93855, GSE77020 (Note: GSM1617847-GSM1617849 and GSM2042608-GSM2042610 are duplicates and represent the same samples) and GSE66242 (Note: 9 goat samples derived from individuals sampled at 2000m altitude were not included in this study). The re-sequencing data for 7 individuals was deposited in the NCBI-sequence read archive (SRA) under accession number SRP096151. Supporting data is also available via the *Gigascience* database, GigaDB (GigaDB, RRID:SCR_004002) [23]. Supplementary figures and tables are provided as Additional Files 1-4.

Ethics statement

All studies involving animals were conducted according to Regulations for the Administration of Affairs Concerning Experimental Animals (Ministry of Science and Technology, China, revised in June 2004). All experimental procedures and sample collection methods in this study were approved by the Institutional Animal Care and Use Committee of the College of Animal Science and Technology of Sichuan Agricultural University, Sichuan, China, under permit No. DKY-B20121406. Animals were allowed free access to food and water under normal conditions, and were humanely sacrificed as necessary, to ameliorate suffering.

Consent for publication

Not applicable.

Competing interests

The authors declare that they have no competing interests.

Funding

This work was supported by grants from the National High Technology Research and Development Program of China (863 Program) (2013AA102502), the National Natural Science Foundation of China (31402046, 31522055, 31601918, 31530073 , 31472081 and 31772576), the Science & Technology Support Program of Sichuan (2016NYZ0042), the Youth Science Fund of Sichuan (2017JQ0011), the China Postdoctoral Science Foundation (2015M572486), China Agriculture Research System (CARS-36), the Program

for Innovative Research Team of Sichuan Province (2015TD0012), the Program for Pig Industry Technology System Innovation Team of Sichuan Province (SCCXTD-005), the Project of Sichuan Education Department (15ZA0008, 15ZA0003, 16ZA0025 and 16ZB0037), the National Program for Support of Top-notch Young Professionals and the Young Scholars of the Yangtze River.

Authors' contributions

MZ.L., QZ.T., YR.G. and XW.L. designed and supervised the project. JQ.G., TD.C., SL.H., Y.L., XM.Y., X.T., ZJ.Z., XH.C., DY.L., XL.L. and XB.L. collected the data, L.J., R.L., J.L., KR.L., SL.T., GS.W., JD.M., X.W., MM.M. and AA.J. generated the data. QZ.T. and MZ.L. performed the bioinformatics analyses. QZ.T. and MZ.L. wrote the manuscript. XM.Z. and VN.G. revised the manuscript.

References

1. Qiu Q, Zhang G, Ma T, Qian W, Wang J, Ye Z, et al. The yak genome and adaptation to life at high altitude. Nat Genet. 2012;44(8):946-9. doi:10.1038/ng.2343.
2. Li M, Tian S, Jin L, Zhou G, Li Y, Zhang Y, et al. Genomic analyses identify distinct patterns of selection in domesticated pigs and Tibetan wild boars. Nat Genet. 2013;45(12):1431-8. doi:10.1038/ng.2811.
3. Li M, Chen L, Tian S, Lin Y, Tang Q, Zhou X, et al. Comprehensive variation discovery and recovery of missing sequence in the pig genome using multiple de

novo assemblies. Genome Res. 2017 May;27(5):865-874. doi:10.1101/gr.207456.116.

4. International Chicken Genome Sequencing Consortium. Sequence and comparative analysis of the chicken genome provide unique perspectives on vertebrate evolution. Nature. 2004;432(7018):695-716. doi:10.1038/nature03154.

5. Groenen MA, Archibald AL, Uenishi H, Tuggle CK, Takeuchi Y, Rothschild MF, et al. Analyses of pig genomes provide insight into porcine demography and evolution. Nature. 2012;491(7424):393-8. doi:10.1038/nature11622.

6. Bovine Genome Sequencing and Analysis Consortium, Elsik CG, Tellam RL, Worley KC, Gibbs RA, et al. The genome sequence of taurine cattle: a window to ruminant biology and evolution. Science. 2009;324(5926):522-8. doi:10.1126/science.1169588.

7. Dong Y, Xie M, Jiang Y, Xiao N, Du X, Zhang W, et al. Sequencing and automated whole-genome optical mapping of the genome of a domestic goat (Capra hircus). Nat Biotechnol. 2013;31(2):135-41. doi:10.1038/nbt.2478.

8. Jiang Y, Xie M, Chen W, Talbot R, Maddox JF, Faraut T, et al. The sheep genome illuminates biology of the rumen and lipid metabolism. Science. 2014;344 (6188):1168-73. doi:10.1126/science.1252806.

9. Vilella AJ, Severin J, Ureta-Vidal A, Heng L, Durbin R and Birney E. EnsemblCompara GeneTrees: Complete, duplication-aware phylogenetic trees in vertebrates. Genome Res. 2009;19(2):327-35. doi:10.1101/gr.073585.107.

10. Li H and Durbin R. Fast and accurate long-read alignment with Burrows-Wheeler transform. Bioinformatics. 2010;26(5):589-95. doi:10.1093/bioinformatics/btp698.

11. McKenna A, Hanna M, Banks E, Sivachenko A, Cibulskis K, Kernytsky A, et al. The Genome Analysis Toolkit: a MapReduce framework for analyzing next-generation DNA sequencing data. Genome Res. 2010;20(9):1297-303. doi:10.1101/gr.107524.110.

12. Li H, Coghlan A, Ruan J, Coin LJ, Heriche JK, Osmotherly L, et al. TreeFam: a

curated database of phylogenetic trees of animal gene families. Nucleic Acids Res. 2006;34(Database issue):D572-80. doi:10.1093/nar/gkj118.

13. Trapnell C, Pachter L and Salzberg SL. TopHat: discovering splice junctions with RNA-Seq. Bioinformatics. 2009;25(9):1105-11. doi:10.1093/bioinformatics/btp120.

14. Trapnell C, Williams BA, Pertea G, Mortazavi A, Kwan G, van Baren MJ, et al. Transcript assembly and quantification by RNA-Seq reveals unannotated transcripts and isoform switching during cell differentiation. Nat Biotechnol. 2010;28(5):511-5. doi:10.1038/nbt.1621.

15. The pvca R package. https://bioconductor.org/packages/release/bioc/html/pvca.html. Accessed Feb 16 2017.

16. Paradis E, Claude J and Strimmer K. APE: Analyses of phylogenetics and evolution in R language. Bioinformatics. 2004;20(2):289-90.

17. Dennis G, Jr., Sherman BT, Hosack DA, Yang J, Gao W, Lane HC, et al. DAVID: Database for annotation, visualization, and integrated discovery. Genome Biol. 2003;4(5):P3.

18. Kent WJ, Sugnet CW, Furey TS, Roskin KM, Pringle TH, Zahler AM, et al. The human genome browser at UCSC. Genome Res. 2002;12(6):996-1006. doi:10.1101/gr.229102.

19. Brawand D, Soumillon M, Necsulea A, Julien P, Csardi G, Harrigan P, et al. The evolution of gene expression levels in mammalian organs. Nature. 2011;478 (7369):343-8. doi:10.1038/nature10532.

20. Merkin J, Russell C, Chen P and Burge CB. Evolutionary dynamics of gene and isoform regulation in Mammalian tissues. Science. 2012;338(6114):1593-9. doi:10.1126/science.1228186.

21. Barbosa-Morais NL, Irimia M, Pan Q, Xiong HY, Gueroussov S, Lee LJ, et al. The evolutionary landscape of alternative splicing in vertebrate species. Science. 2012;338(6114):1587-93. doi:10.1126/science.1230612.

22. Mele M, Ferreira PG, Reverter F, DeLuca DS, Monlong J, Sammeth M, et al. Human genomics. The human transcriptome across tissues and individuals. Science. 2015;348(6235):660-5. doi:10.1126/science.aaa0355.

23. Tang Q, Gu Y, Zhou X, Jin L, Guan J, Liu R, et al. Supporting data for "Comparative transcriptomics of five high-altitude vertebrates and their low-altitude relatives". *GigaScience* Database. 2017. http://dx.doi.org/10.5524/100355

Figures 1-4

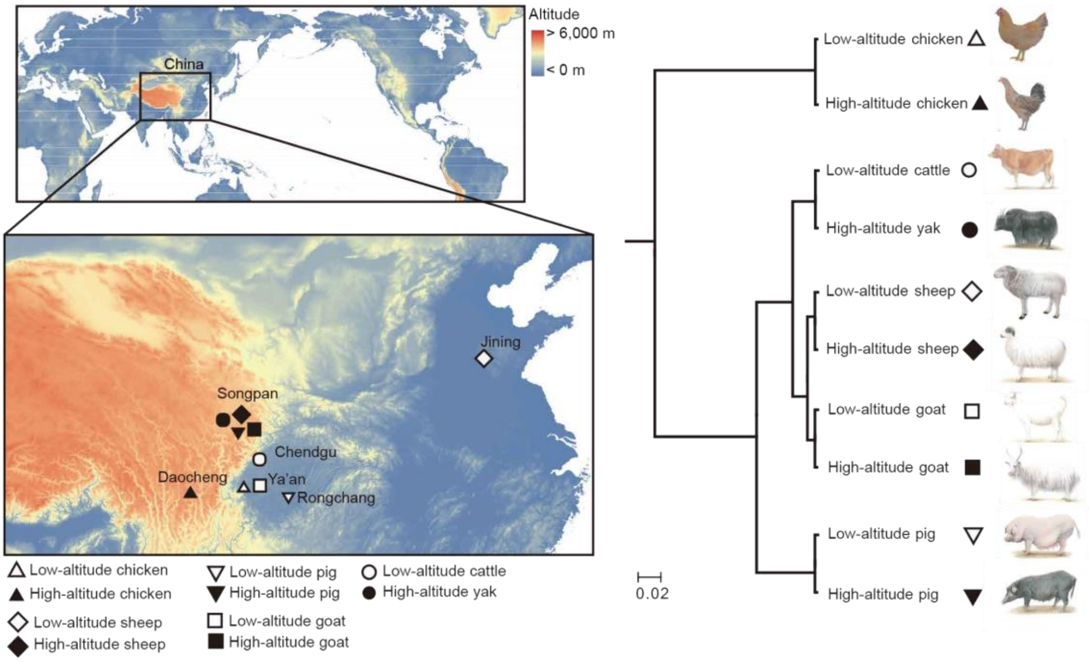

Figure 1. Sampling locations and nucleotide alignment-based tree.

(a) Geographic locations of the studied animals.

(b) A neighbour-joining tree constructed based on concatenated coding sequences of single-copy orthologues substituted by SNVs and InDels detected in each animal.

We downloaded and extracted the unassembled reads from short-insert (500 bp) libraries of a single yak [1], a Tibetan pig [2] and a Rongchang pig [3] that were used for *de novo* assemblies to roughly 10 × depth coverage. We also randomly selected an individual of the cattle, low- and high-altitude chicken, goat and sheep and sequenced the whole genomes at ~10 × depth coverage.

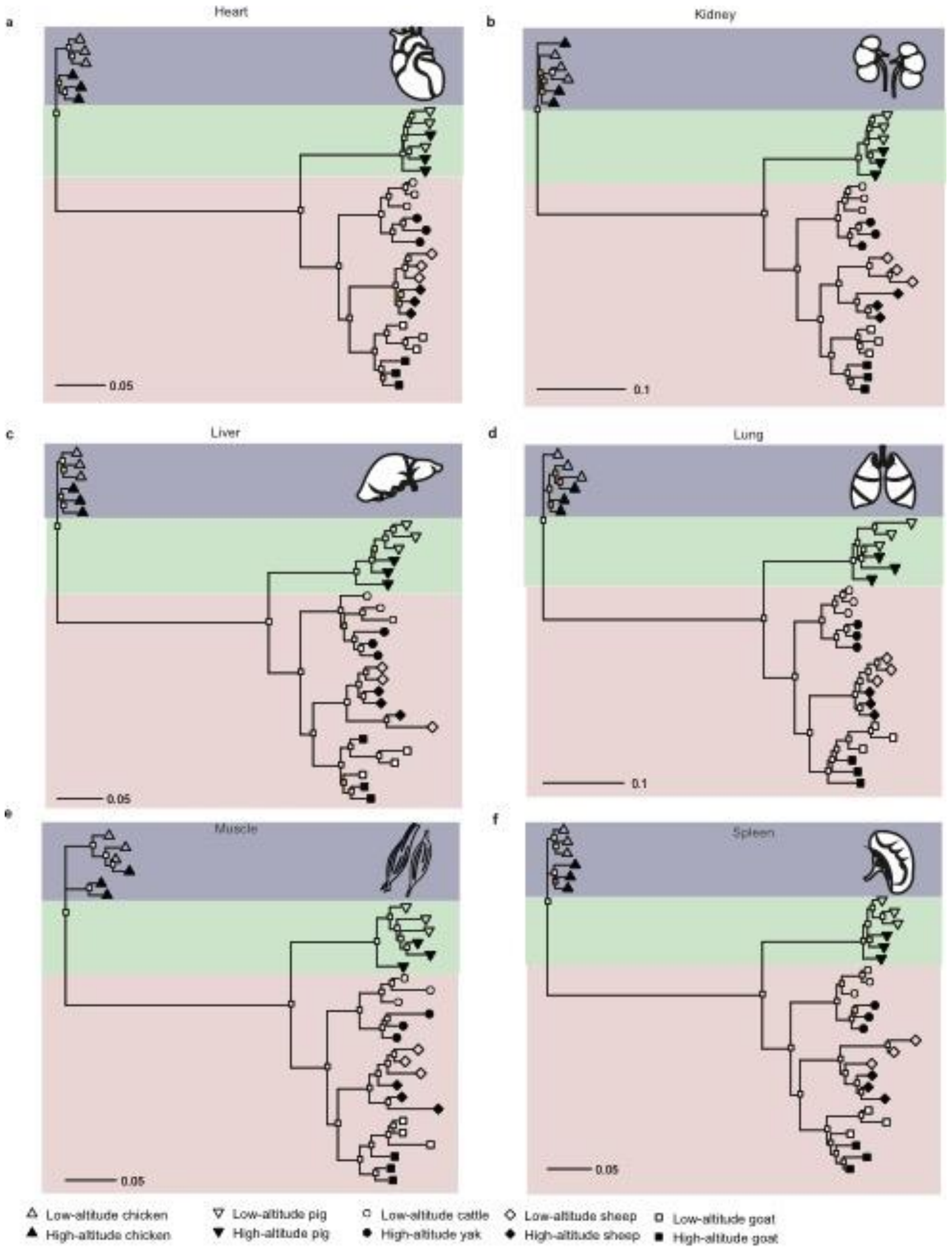

Figure 2. Gene expression phylogenies for six tissues across five vertebrates. Neighbour-joining expression tree constructed based on (1-Spearman correlation) distances in six tissues. We performed 100 bootstraps by randomly sampling the single copy orthologues with replacement. Bootstrap values (fractions of replicate trees that have the branching pattern as in the shown tree constructed using all the transcribed single copy orthologues) are indicated by different colors: red color of the node indicates support from

less than 50% bootstraps, while orange, yellow and white colors indicate support between 50% and 70%, between 70% and 90% and more than 90%, respectively.

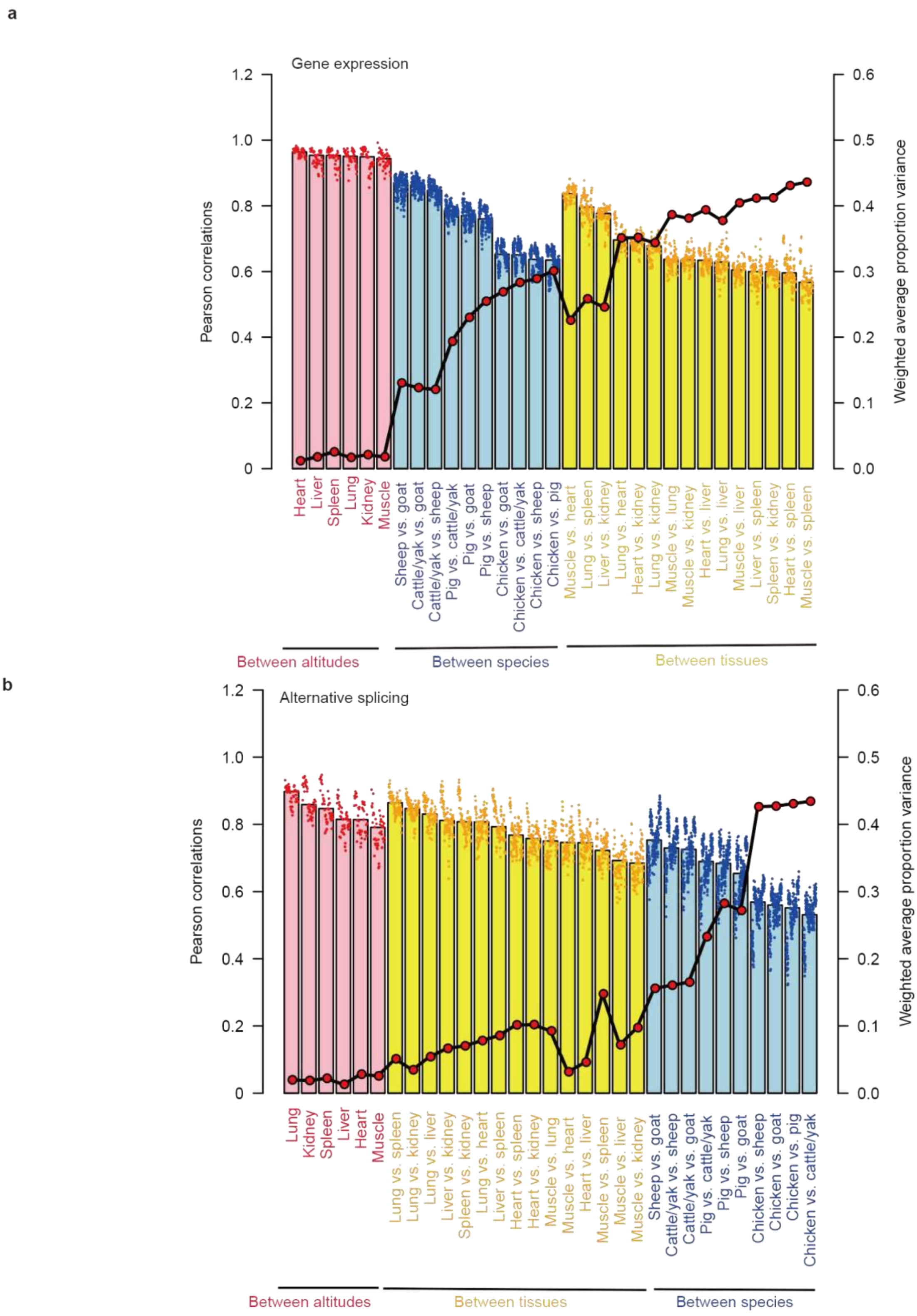

Figure 3. Comparison of variations between altitudes, species and tissues revealed by (a) gene expression and (b) alternative splicing pattern.

Scatter-point and bar plots represent the pairwise Pearson's correlation between samples. Weighted average proportion variance of the alternative splicing (reflected by PSI values) were determined using the Principal Variance Component Analysis (PVCA) approach and depicted as red dots connected by black lines.

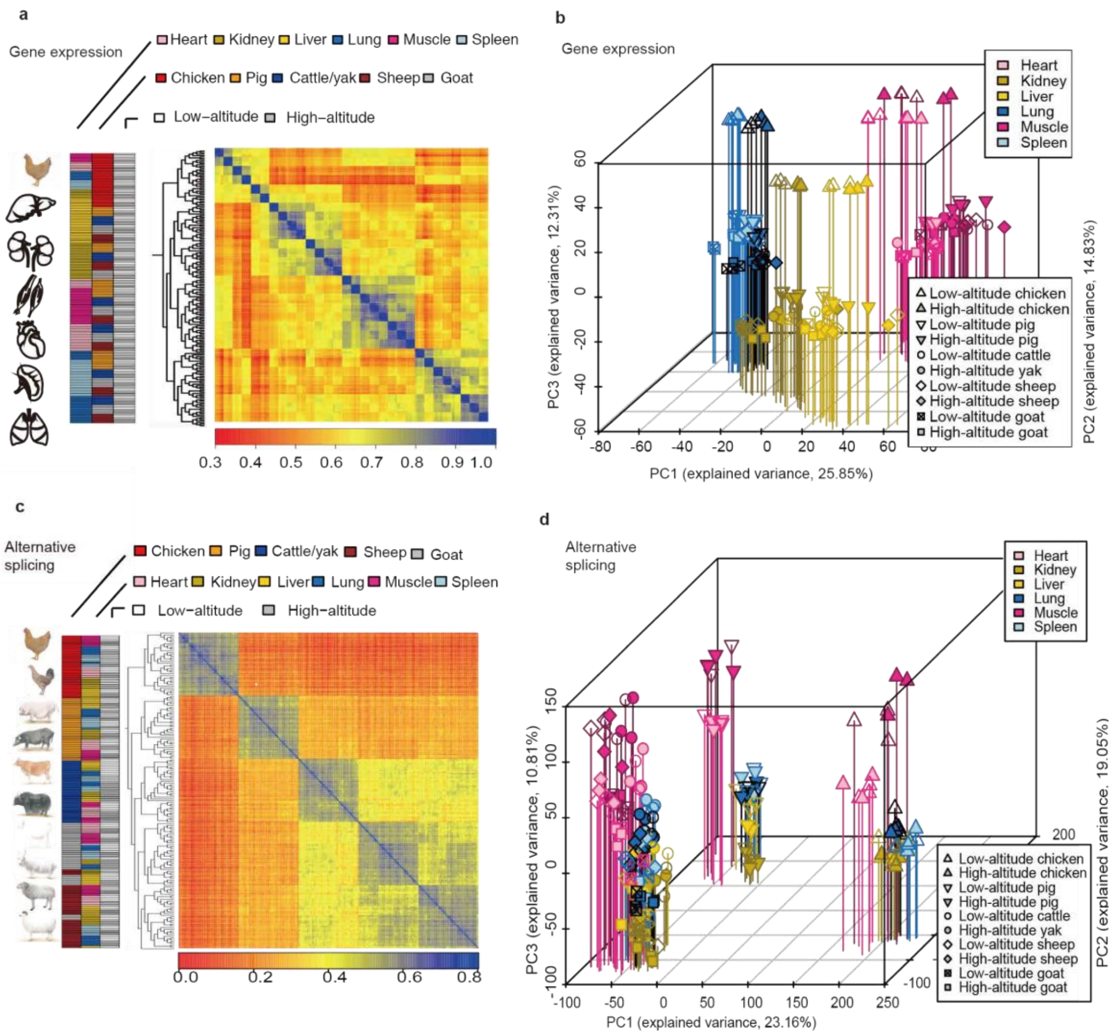

Figure 4. Global pattern of gene expression and alternative splicing pattern.

Hierarchical clustering of samples using **(a)** the gene expression and **(c)** the alternative splicing (reflected by PSI values). Average linkage hierarchical clustering was used with distance between samples measured by the Pearson's correlation between the vectors of expression values. Factorial map of the principal-component analysis (PCA) of **(b)** gene

expression levels and **(d)** the alternative splicing. The proportion of the variance explained by the principal components is indicated in parentheses. The vertical leading lines with different colors from the plotted points dropping to the xy-plane show the separation of points based on the first and second principal components.

Table 1. Number of DEGs between five high-altitude vertebrates and their low-altitude relatives for each tissue

Species	Heart	Kidney	Liver	Lung	Muscle	Spleen	Mean
**Chicken**	1283 (8.28%)	748 (4.83%)	613 (3.96%)	1072 (6.92%)	177 (1.14%)	984 (6.35%)	812 (5.25%)
**Pig**	206 (0.95%)	532 (2.46%)	1199 (5.55%)	426 (1.97%)	385 (1.78%)	994 (4.60%)	623 (2.89%)
**Cattle/yak**	1602 (8.02%)	1797 (8.99%)	869 (4.35%)	3092 (15.47%)	2403 (12.03%)	2268 (11.35%)	2005 (10.04%)
**Sheep**	1332 (6.37%)	3853 (18.43%)	259 (1.24%)	1829 (8.75%)	1079 (5.16%)	2356 (11.27%)	1784 (8.54%)
**Goat**	2215 (10.01%)	3557 (16.07%)	655 (2.96%)	1330 (6.01%)	2305 (10.42%)	1269 (5.73%)	1888 (8.53%)
**Mean**	1327 (6.73%)	2097 (10.16%)	719 (3.61%)	1549 (7.82%)	1269 (6.11%)	1574 (7.86%)	

Percentage of the DGEs compared with the total number of annotated protein coding genes in their respective reference genomes are listed in parenthesis. There are 15495, 21594, 19981, 22131, 20908 annotated protein coding genes in reference genomes of Chicken (Galgal4) [4], pig (Suscrofa 10.2) [5], cattle (UMD3.1) [6], goat (CHIR_1.0) [7] and sheep (Oar_v3.1) [8], respectively.

 Click here to download Table Table1.docx

Table 1. Number of DEGs between five high-altitude vertebrates and their low-altitude relatives for each tissue

Species	Heart	Kidney	Liver	Lung	Muscle	Spleen	Mean
**Chicken**	1283 (8.28%)	748 (4.83%)	613 (3.96%)	1072 (6.92%)	177 (1.14%)	984 (6.35%)	812 (5.25%)
**Pig**	206 (0.95%)	532 (2.46%)	1199 (5.55%)	426 (1.97%)	385 (1.78%)	994 (4.60%)	623 (2.89%)
**Cattle/yak**	1602 (8.02%)	1797 (8.99%)	869 (4.35%)	3092 (15.47%)	2403 (12.03%)	2268 (11.35%)	2005 (10.04%)
**Sheep**	1332 (6.37%)	3853 (18.43%)	259 (1.24%)	1829 (8.75%)	1079 (5.16%)	2356 (11.27%)	1784 (8.54%)
**Goat**	2215 (10.01%)	3557 (16.07%)	655 (2.96%)	1330 (6.01%)	2305 (10.42%)	1269 (5.73%)	1888 (8.53%)
**Mean**	1327 (6.73%)	2097 (10.16%)	719 (3.61%)	1549 (7.82%)	1269 (6.11%)	1574 (7.86%)	

Percentage of the DGEs compared with the total number of annotated protein coding genes in their respective reference genomes are listed in parenthesis. There are 15495, 21594, 19981, 22131, 20908 annotated protein coding genes in reference genomes of Chicken (Galgal4) [4], pig (Suscrofa 10.2) [5], cattle (UMD3.1) [6], goat (CHIR_1.0) [7] and sheep (Oar_v3.1) [8], respectively.

Figure 1

Click here to download Figure Figure1.pdf

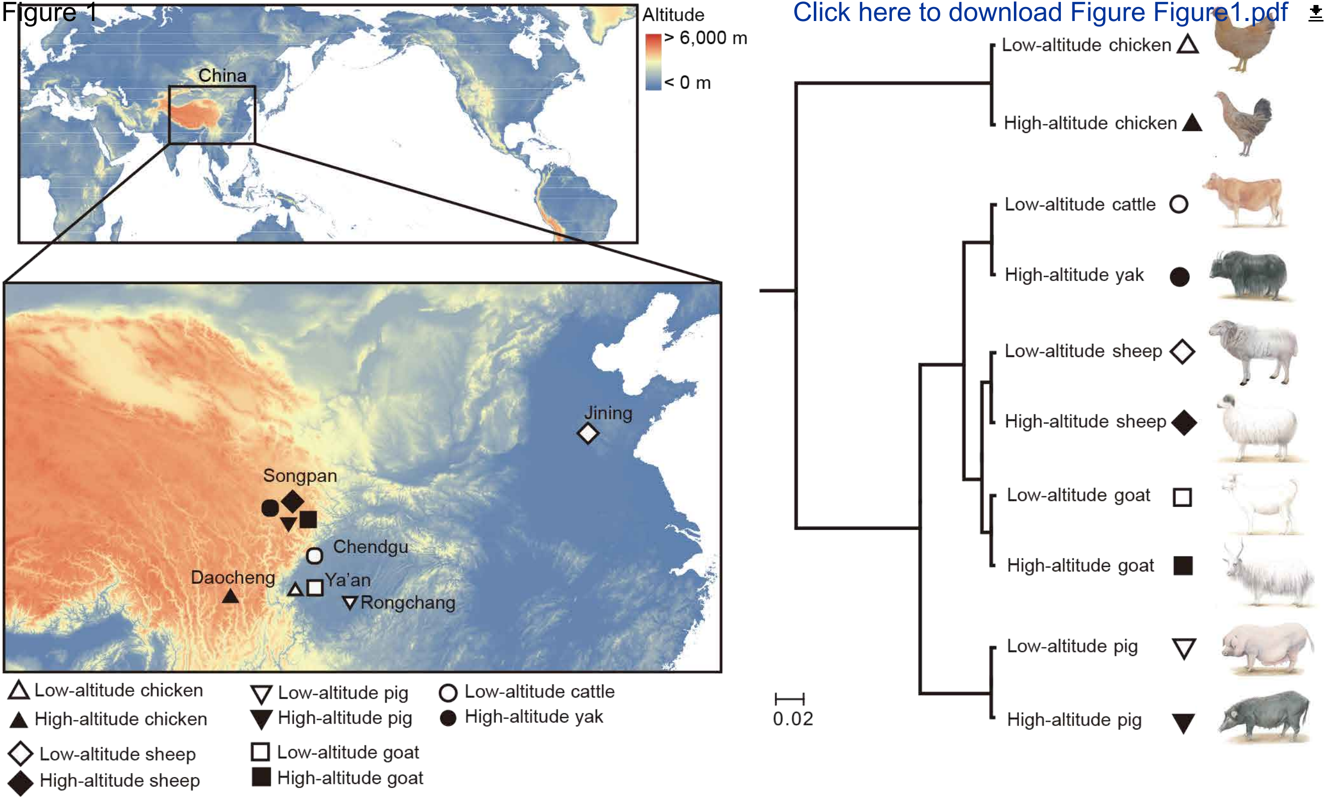

Figure 2

Click here to download Figure Figure2.pdf

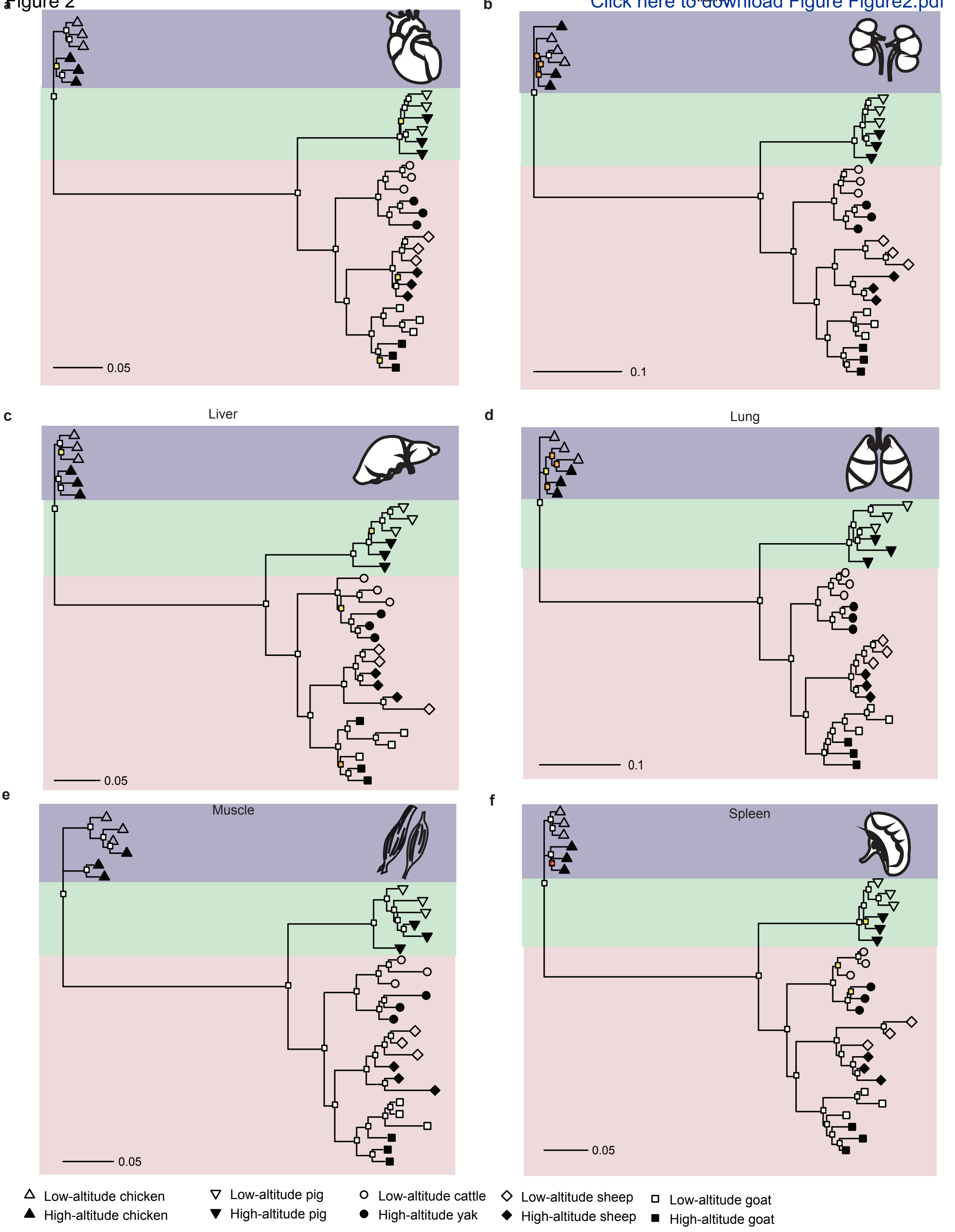

Figure 3 Click here to download Figure Figure3.pdf

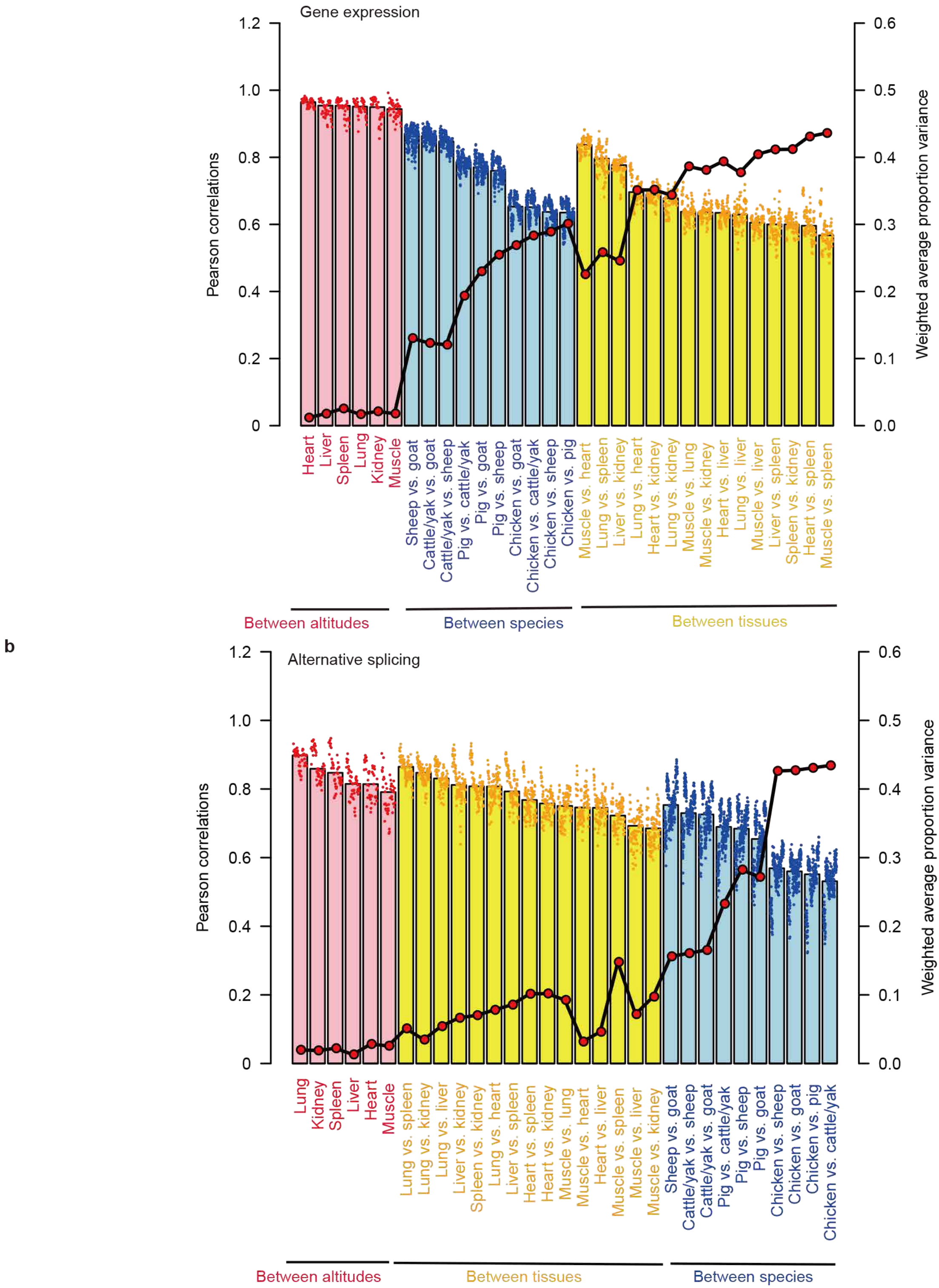

Figure 4

Click here to download Figure Figure4.pdf

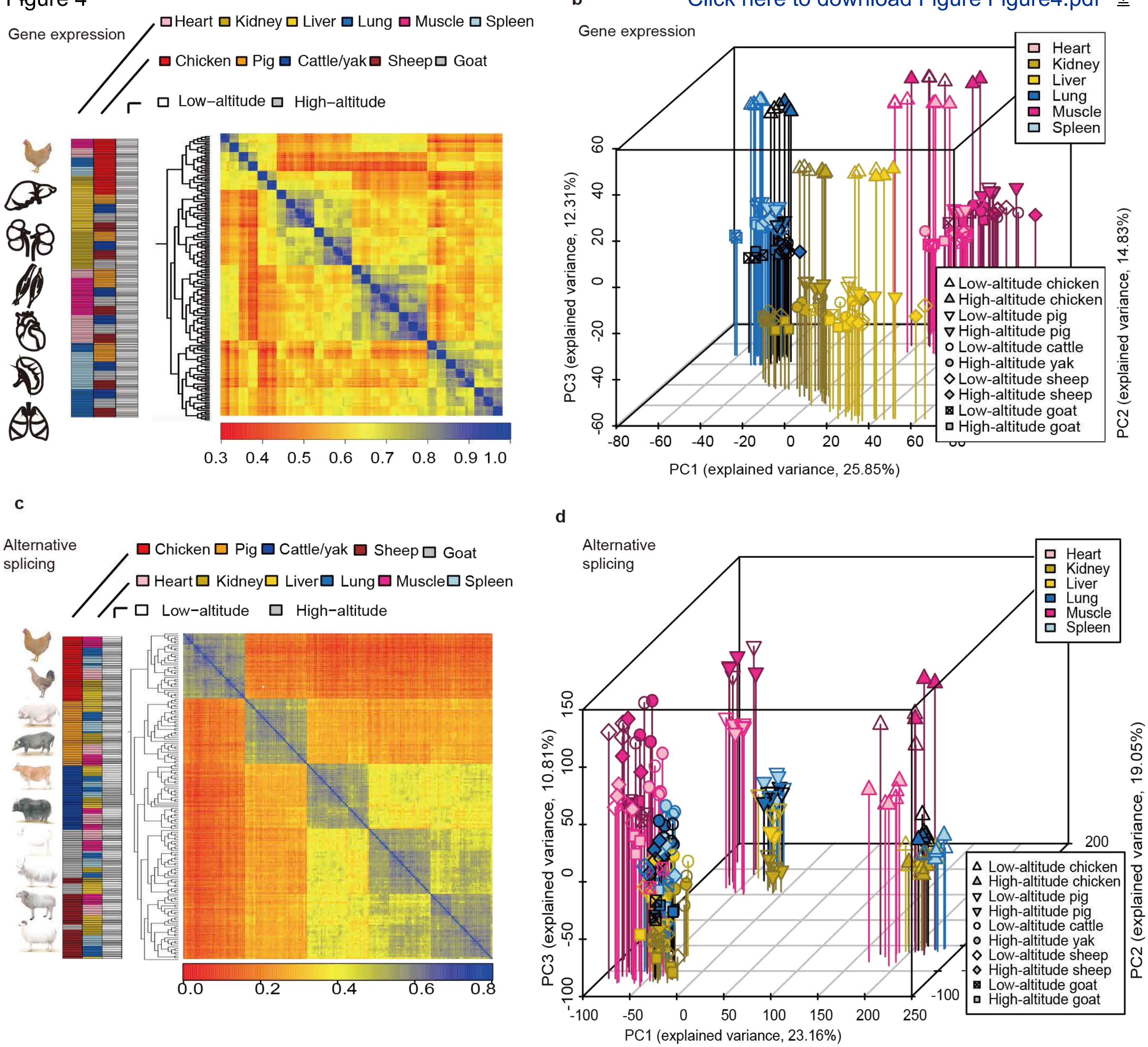

Additional File 1

Click here to access/download

Supplementary Material

Additional_file_1.pdf

Additional File 2

Click here to access/download
Supplementary Material
Additional_File_2.xlsx

Click here to access/download
Supplementary Material
Additional_File_3.xlsx

Additional File 4

Click here to access/download
Supplementary Material
Additional_File_4.xlsx

Response_sup

Click here to access/download
Supplementary Material
Response_sup.pdf

GigaScience

em@editorialmanager.com

Dear Dr. Hans Zauner,

We are delighted to be informed of the positive responses from you. We have carefully revised the manuscript following your and review 2's suggestions, and uploaded it online for formal acceptance and publication.

We have released all of datasets related to our manuscript, including a total of 180 RNA-seq data deposited in the NCBI Gene Expression Omnibus (GEO) under accession numbers GSE93855, GSE77020 and GSE66242, as well as the 7 whole-genome sequencing data deposited in the NCBI-sequence read archive (SRA) under accession number SRP096151.

Moreover, to promote the clarity of depiction for the principal component analysis (PCA) results, we newly added the two-dimensional PCA figures as the **Supplementary Figs. S14-S15**.

We sincerely appreciate your assistance in improving the manuscript. We are glad to be able to contribute to *GigaScience*.

Best regards,

Dr. Mingzhou Li

Sichuan Agricultural University, Chengdu, Sichuan, China

Email: mingzhou.li@sicau.edu.cn

Detailed responses to editor

All comments provided by editor are in gray italics, and our responses are in black. Important revisions in the manuscript are marked in red.

Editor: Dr. Hans Zauner

Comment 1-1:

Before we proceed to acceptance, please address the minor additional comment of reviewer 2 (regarding Fig. 4 - see below).

Formatted: Justified, Line spacing: 1.5 lines

Response 1-1:

Thank you for your reminder. We have carefully revised **Fig. 4** according to reviewer 2's suggestion. (Please see **Response 2-3** for details)

Comment 1-2:

In preparation for publication, please also remove all highlighting/tracking that was added for the purpose of review. Please also be aware that prior to publication, all data has to be openly available (e.g. via NCBI - please lift any embargoes etc.).

Response 1-2:

Thank you for your reminder. We have removed all highlighting/tracking that was added for the purpose of review and prepared the manuscripts for publication.

We have released all of dataset related to our manuscript, inducing a total of 180 RNA-seq data deposited in the NCBI Gene Expression Omnibus (GEO) under accession numbers GSE93855, GSE77020 and GSE66242, as well as

7 whole-genome sequencing data deposited in the NCBI-sequence read archive (SRA) under accession number SRP096151.

GSE93855: https://www.ncbi.nlm.nih.gov/geo/query/acc.cgi?acc=GSE93855
GSE77020: https://www.ncbi.nlm.nih.gov/geo/query/acc.cgi?acc=GSE77020
GSE66242: https://www.ncbi.nlm.nih.gov/geo/query/acc.cgi?acc=GSE66242
SRP096151: https://trace.ncbi.nlm.nih.gov/Traces/sra/?study=SRP096151

Comment 1-3:

I note you indicate several corresponding authors - please note that we can consider only one author with this role. Please discuss this with your coauthors and indicate only one corresponding author in your revised manuscript.

Response 1-3:

Thank you for your reminder. We have thoroughly discussed the corresponding authorship issue with all of our co-authors, and only indicated two corresponding authors and three equally contributing first authors in the revised manuscript.
