# Supplementary material for: Comparative transcriptomics of 5 high-altitude vertebrates and their low-altitude relatives
Source: Gigascience. 2017 Nov 15;6(12):1–9. doi: 10.1093/gigascience/gix105 (PMC5729692; doi:10.1093/gigascience/gix105)
Supplement: Additional file 1 [file gix105_additional_file_1.pdf]

Supplementary Figure S1. Summary and phenotypic characteristics of sampled animals.

Species	Animals	Breed / population description	Location	Average altitude (m)	Age	Body weight (kg)	Forms of livestock rearing
**Cattle (*Bos taurus*)**[a]	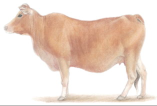	Indigenous cattle (low-altitude)	Chengdu city, Sichuan province, China	~500	Adult (>4 years old)	~300	Home rearing
**Yak (*Bos grunniens*)**[a]	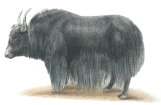	Yak (high-altitude)	Songpan prefecture, Tibetan autonomous region, China	~3000	Adult (>4 years old)	~200	Stocking / nomadism
**Goat (*Capra aegagrus hircus*)**[b]	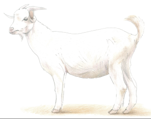	Indigenous goat (low-altitude)	Ya'an city, Sichuan province, China	~600	Adult (>3 years old)	30-50	Home rearing
	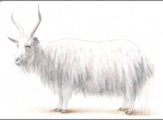	Tibetan goat (high-altitude)	Songpan prefecture, Tibetan autonomous region, China	~3000	Adult (>3 years old)	~27	Stocking / nomadism
**Sheep (*Ovis aries*)**[b]	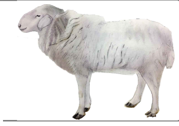	Small tailed Han sheep (low-altitude)	Jining city, Shandong province, China	~50	Adult (>3 years old)	60-70	Home rearing
	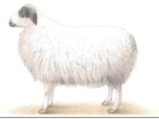	Tibetan sheep (high-altitude)	Songpan prefecture, Tibetan autonomous region, China	~3000	Adult (>3 years old)	30-40	Stocking / nomadism
**Pig (*Sus scrofa*)**[c]	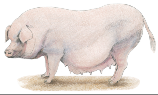	Rongchang pig (low-altitude)	Rongchang prefecture, Chongqing city, China	~400	Adult (>2 year old)	~160	Home rearing
	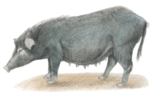	Tibetan pig (high-altitude)	Songpan prefecture, Tibetan autonomous region, China	~3000	Adult (>2 year old)	~88	Stocking / nomadism
**Chicken (*Gallus gallus*)**[d]	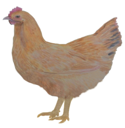	Indigenous chicken (low-altitude)	Ya'an city, Sichuan province, China	~600	Adult (>300 days old)	2.4-2.5	Home rearing
	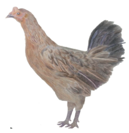	Tibetan chicken (high-altitude)	Daocheng prefecture,, Tibetan autonomous region, China	~3000	Adult (>300 days old)	1.4-1.6	Stocking / nomadism

a. Zhang Y, Xu SZ, Wang ZH, Wang GL, Wang YC, Li JY, et al. 2011. Animal genetic resources in China: bovines. (ed. China National Commission of Animal Genetic Resources), pp. 24-422. China Agricultural Press, Beijing

b. Du LX, Li JQ, Ma N, Ma YH, Wang JM, Ying CA, et al. 2011. Animal genetic resources in China: sheep and goats. (ed. China National Commission of Animal Genetic Resources), pp. 20-445. China Agricultural Press, Beijing

c. Wang LY, Wang AG, Wang LX, Li K, Yang GS, He RG, et al. 2011. Animal genetic resources

in China: pigs. (ed. China National Commission of Animal Genetic Resources), pp. 18-470. China Agricultural Press, Beijing

d. Chen WK, Yang N, Wang GY, Wang ZY, Wang KH, Wang JY, et al. 2011. Animal genetic resources in China: poultry. (ed. China National Commission of Animal Genetic Resources), pp. 16-376. China Agricultural Press, Beijing

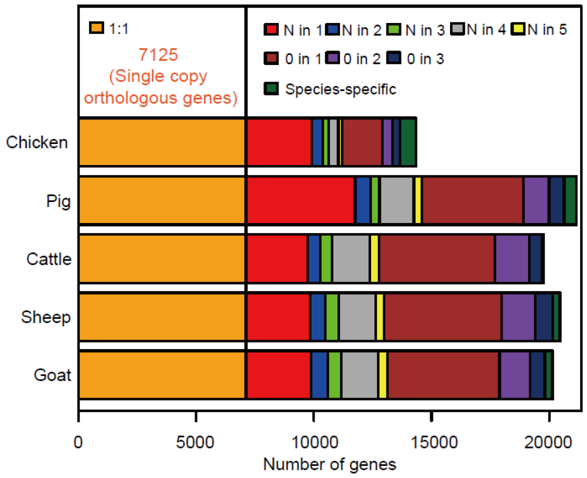

Supplementary Figure S2. Orthology assignment of the five reference veterbrates genomes. Subdivided bars represent different types of orthology relationships. '1:1' indicates single-copy orthologs in each genome. 'N in 1', 'N in 2', 'N in 3', 'N in 4', 'N in 5' indicate multi-copy orthologs in one, two, three, four or all five genomes, respectively. '0 in 1', '0 in 2', and '0 in 3' indicate genes with single- or multi-copy orthologs in only one, two or three genomes, separately. The 'Species-specific' genes exhibit no orthology with genes in the other four genomes.

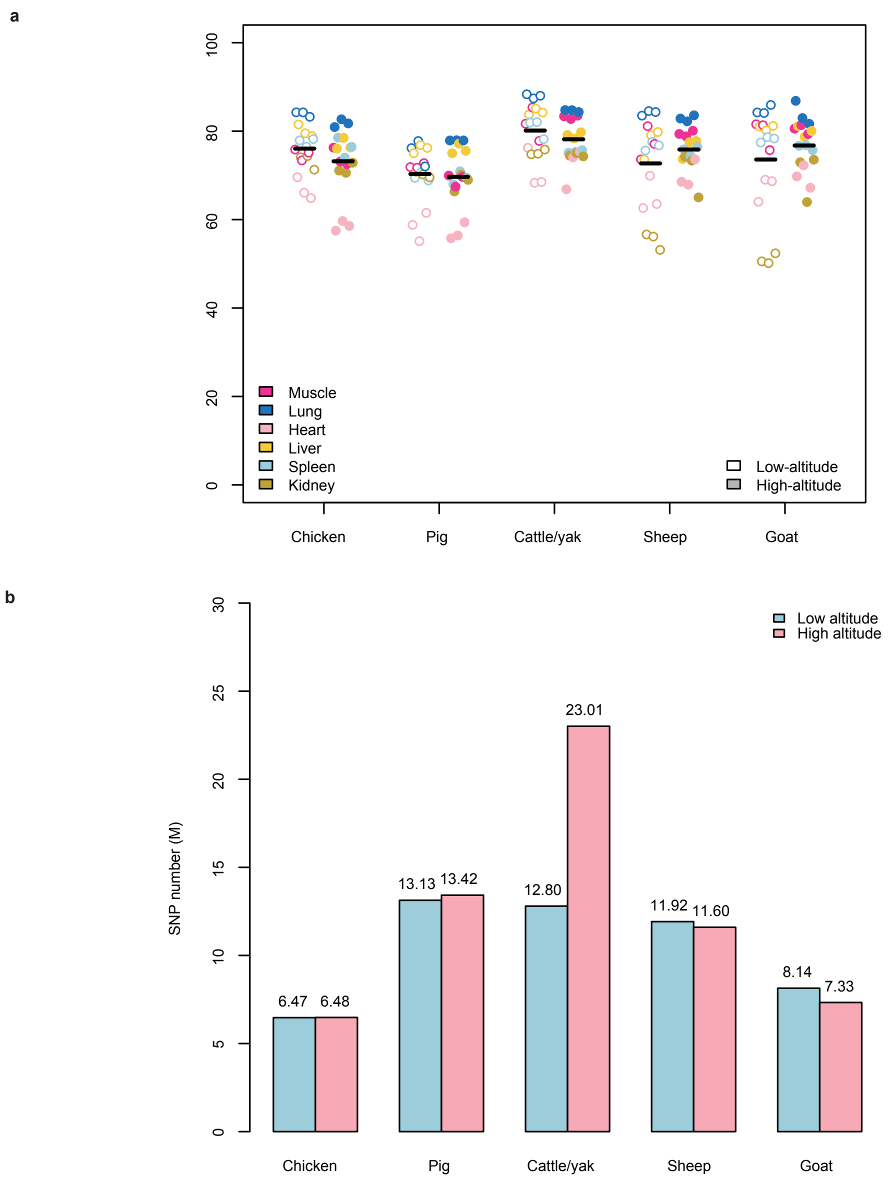

Supplementary Figure S3. Mapping ratios of RNA-seq data (a) and SNP number based on whole-genome sequence data (b).

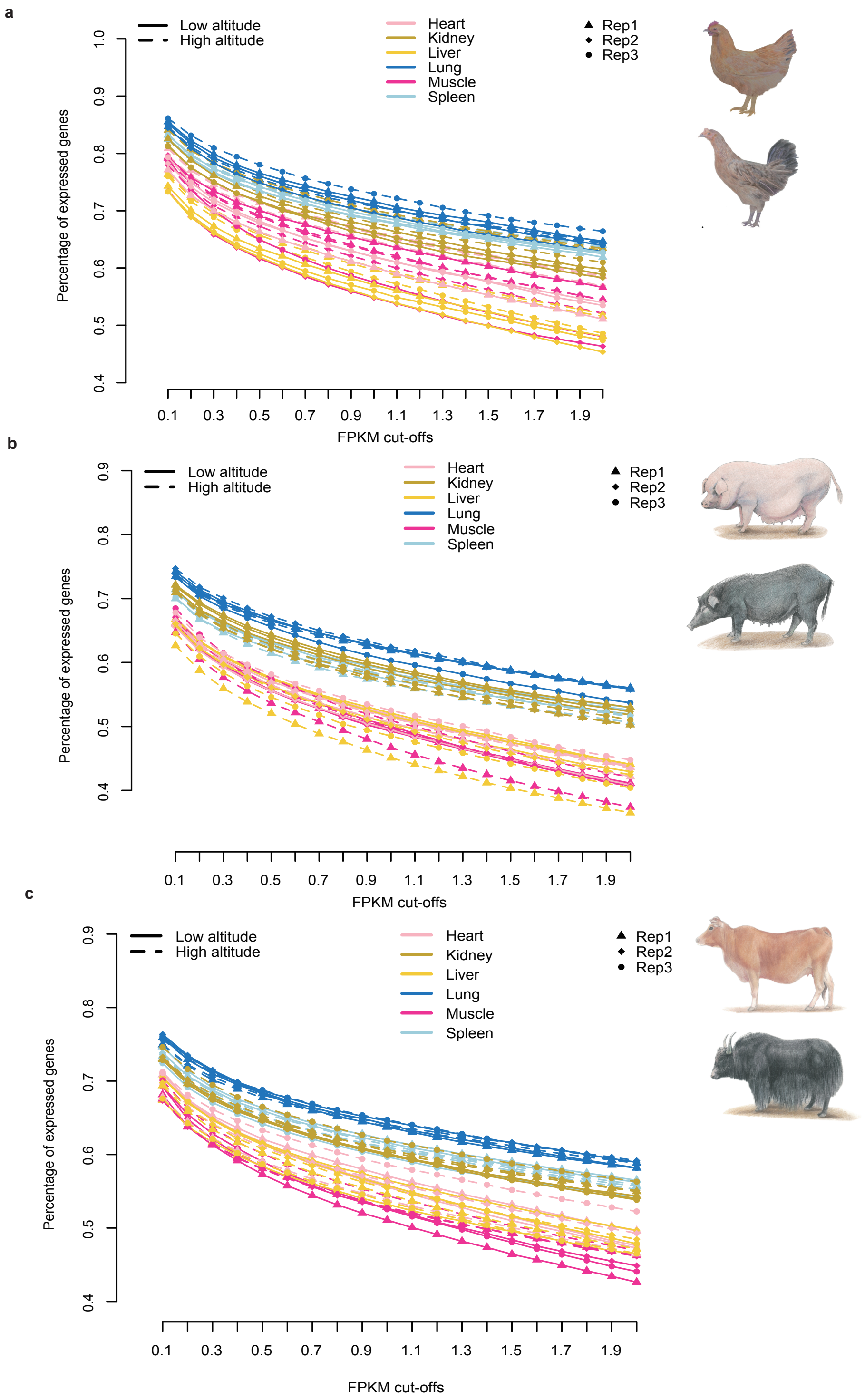
a
Low altitude
High altitude
Heart
Kidney
Liver
Lung
Muscle
Spleen
Rep1
Rep2
Rep3
Percentage of expressed genes
0.4
0.5
0.6
0.7
0.8
0.9
1.0
0.1
0.3
0.5
0.7
0.9
1.1
1.3
1.5
1.7
1.9
FPKM cut-offs
b
Low altitude
High altitude
Heart
Kidney
Liver
Lung
Muscle
Spleen
Rep1
Rep2
Rep3
Percentage of expressed genes
0.4
0.5
0.6
0.7
0.8
0.9
0.1
0.3
0.5
0.7
0.9
1.1
1.3
1.5
1.7
1.9
FPKM cut-offs
c
Low altitude
High altitude
Heart
Kidney
Liver
Lung
Muscle
Spleen
Rep1
Rep2
Rep3
Percentage of expressed genes
0.4
0.5
0.6
0.7
0.8
0.9
0.1
0.3
0.5
0.7
0.9
1.1
1.3
1.5
1.7
1.9
FPKM cut-offs

d

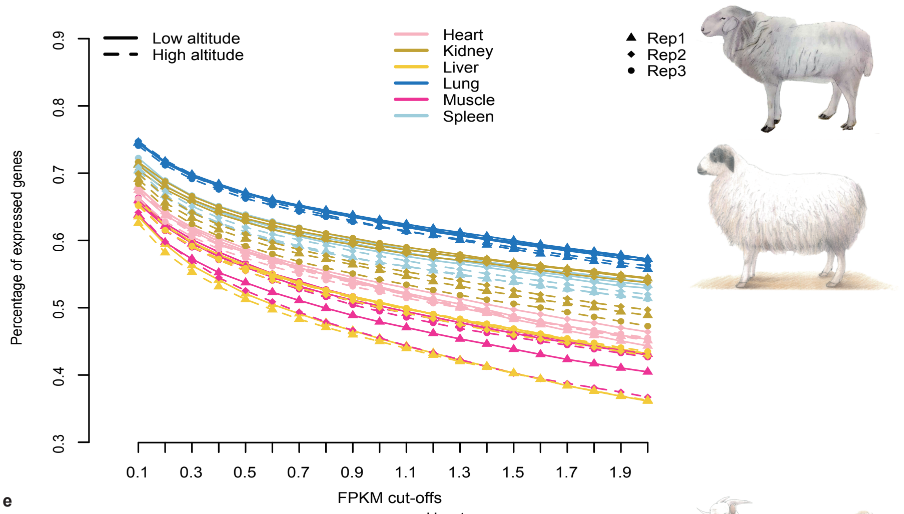

e

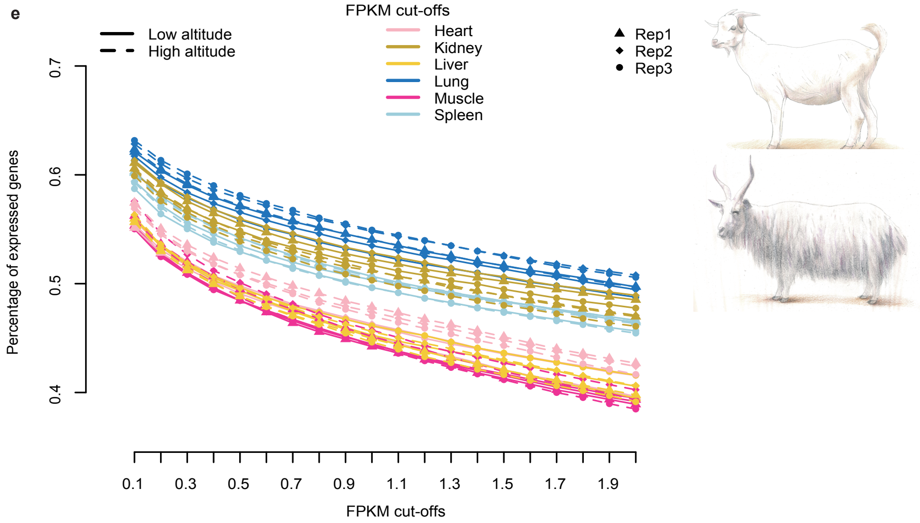

Supplementary Figure S4. Percentage of expressed genes based on distinct cut-offs in each sample. X-axis indicates distinct FPKM cut-offs for defining expressed genes, and y-axis indicates the precentage of expressed genes. Different colors represent the six tissues; different line types represent low- and high-altitude populations; different shapes represent the three individuals (replicates) sampled in each population. **(a) (b) (c) (d)** and **(e)** are results from chicken, pig, cattle/yak, sheep and goat.

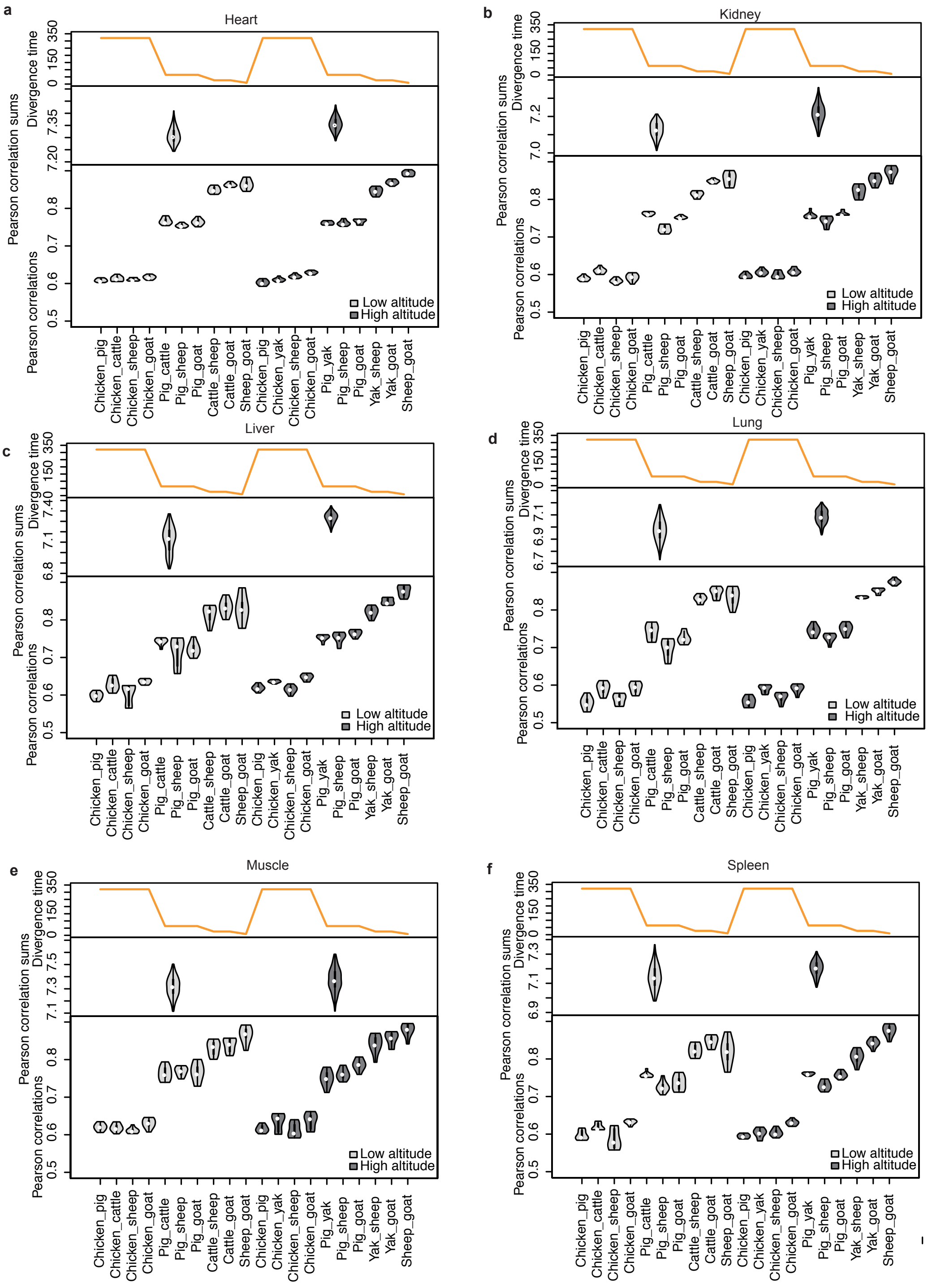

Supplementary Figure S5. Correlations based expression levels and divergence time. The top panel shows the divergence time between pairs of species. The middle panel includes sums of Spearman correlations between all possible species pair combinations across the five studied species of each tissue and altitude group. We performed 100 bootstraps to generate each violin plot by randomly sampling one individual from each population each time. The bottom panel shows the Spearman correlations between each specific species pair combination indicated by x-axis labels. Interestingly, the correlations in bottom panel increase as the divergence time in the top panel decreases. The divergence times were derived from

the TimeTree database (http://www.timetree.org/).

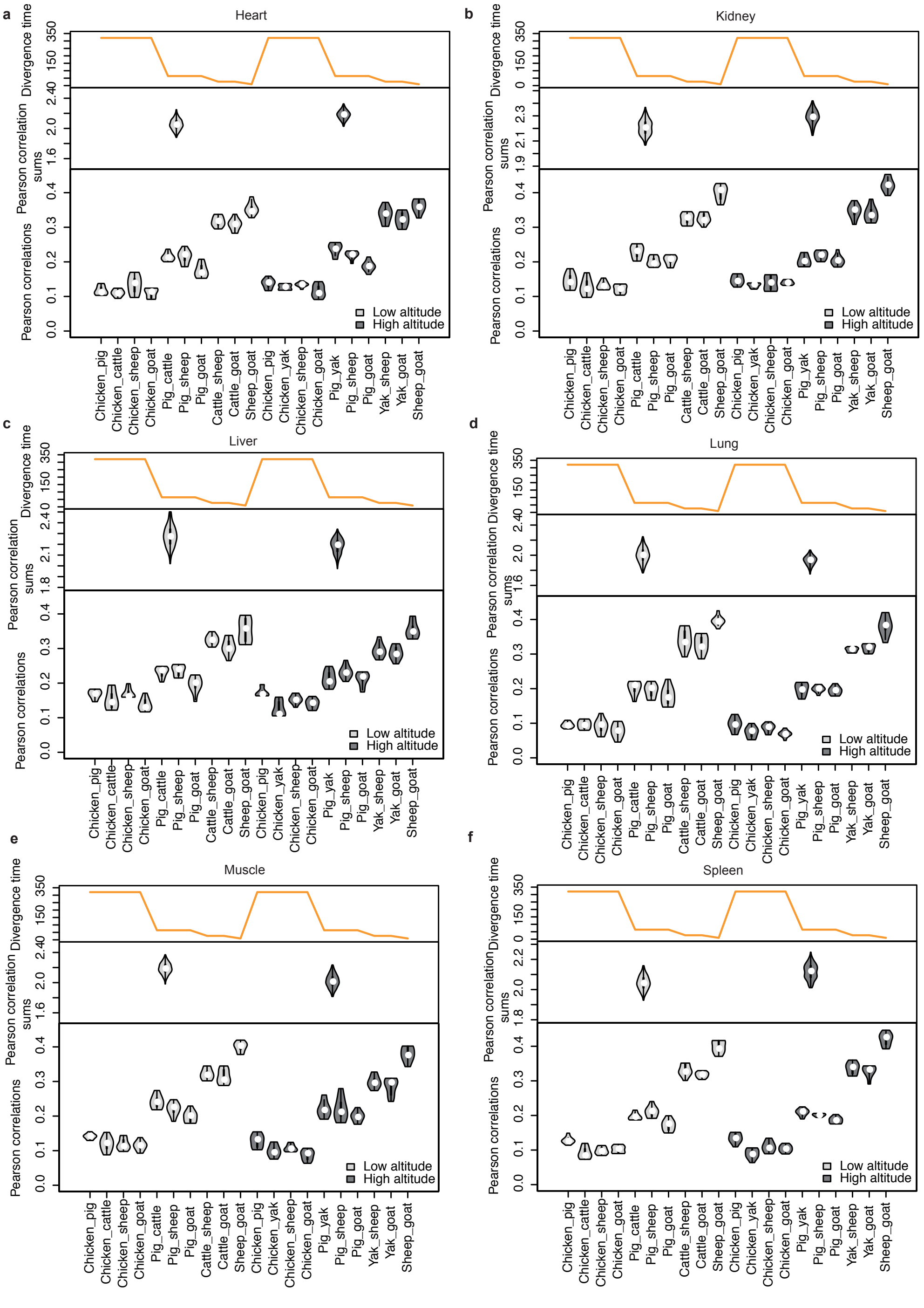

Supplementary Figure S6. Correlations based on PSI values and divergence time. The top panel shows the divergence time between pairs of species. The middle panel includes sums of Spearman correlations between all possible species pair combinations across the five studied species of each tissue and altitude group. We performed 100 bootstraps to generate each violin plot by randomly sampling one individual from each population each time. The bottom panel shows the Spearman correlations between each specific species pair combination indicated by x-axis labels. Interestingly, the correlations in bottom panel increase as the divergence time in the top panel decreases.

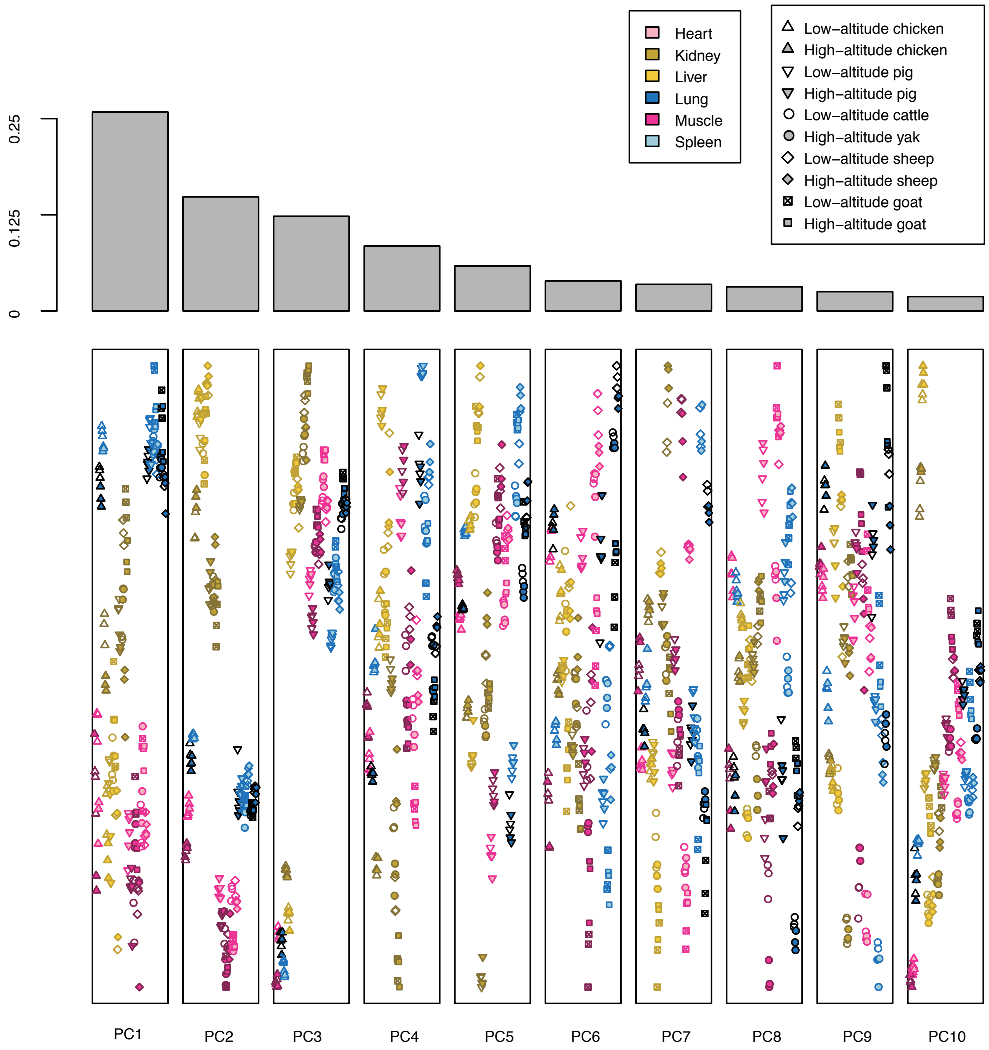

Supplementary Figure S7. The first ten components of PCA based on expression levels. The top panel indicates the percentages of variances explained by each component. The bottom panel depicts the samples placed vertically based on rotated data along each component. Interestingly, the third component sorts chicken out, the fifth component separates pig from the other species, the seventh component splits sheep from the others, and ninth component clusters cattle/yak together, indicating species-specific expression divergence accounts for a non-trivial part of the total divergence, though could not transcend that of tissues.

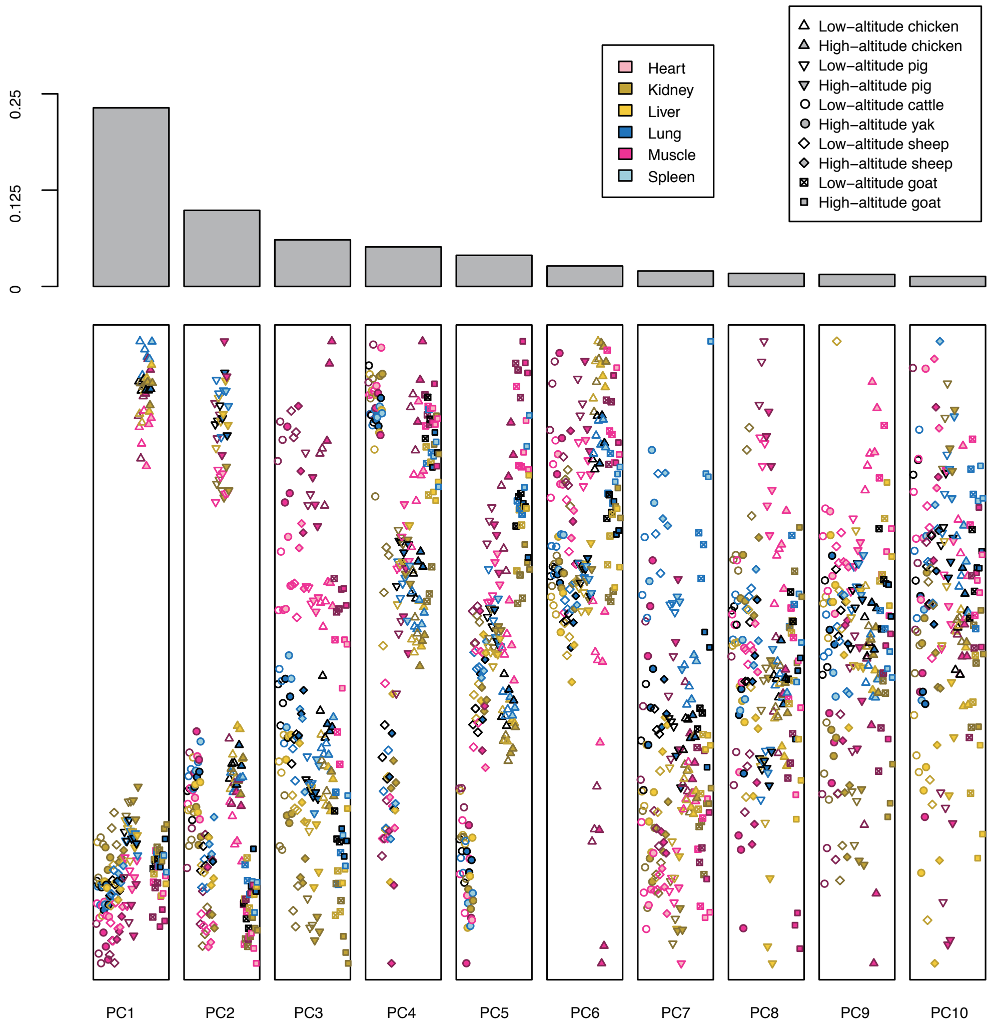

Supplementary Figure S8. The first ten components of PCA based on PSI values. The top panel indicates the percentages of variances explained by each component. The bottom panel depicts the samples placed vertically based on rotated data along each component.

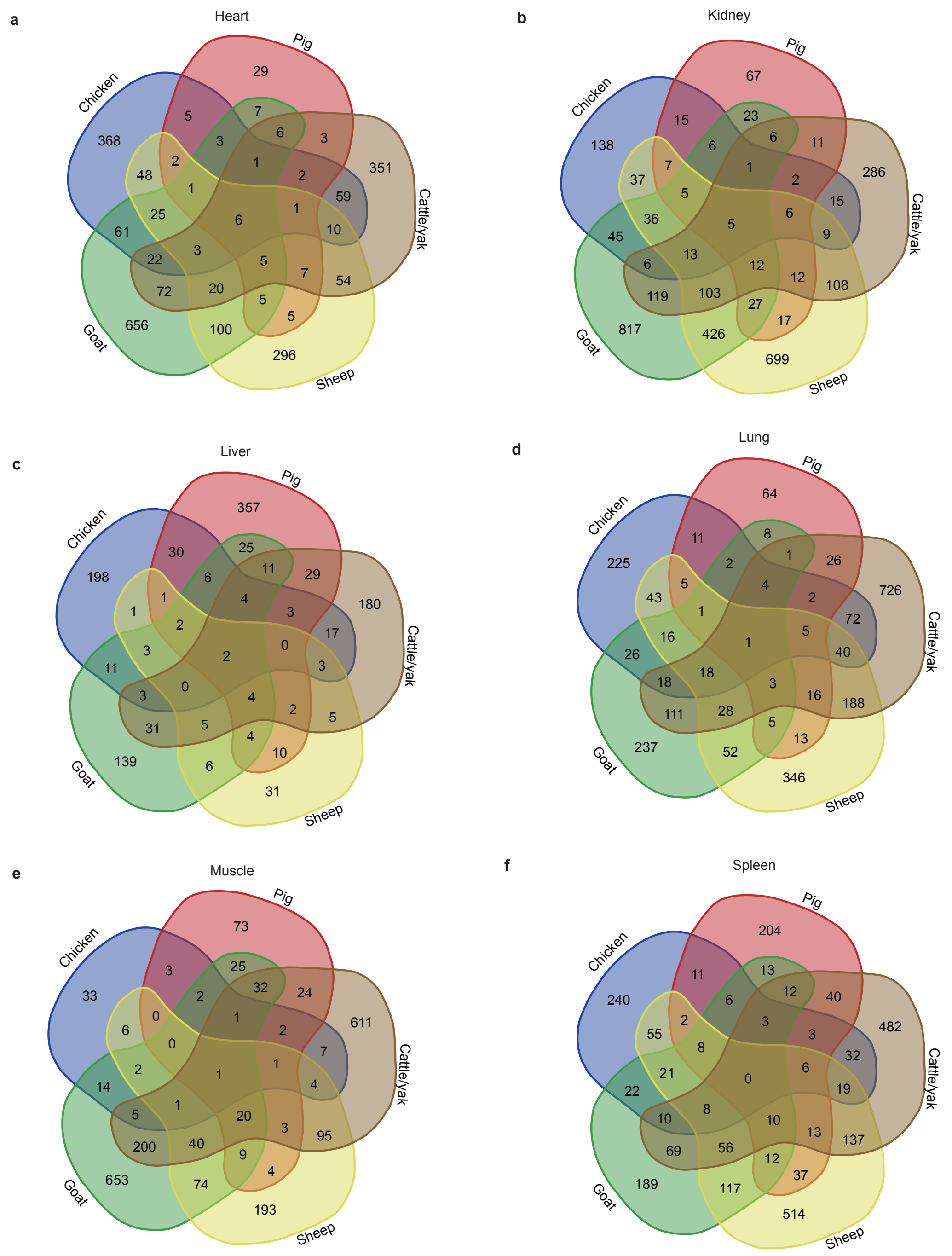

Supplemental Figure S9. Distribution of shared and specific DE genes between the high- and low-altitude populations in each tissue among five vertebrates.

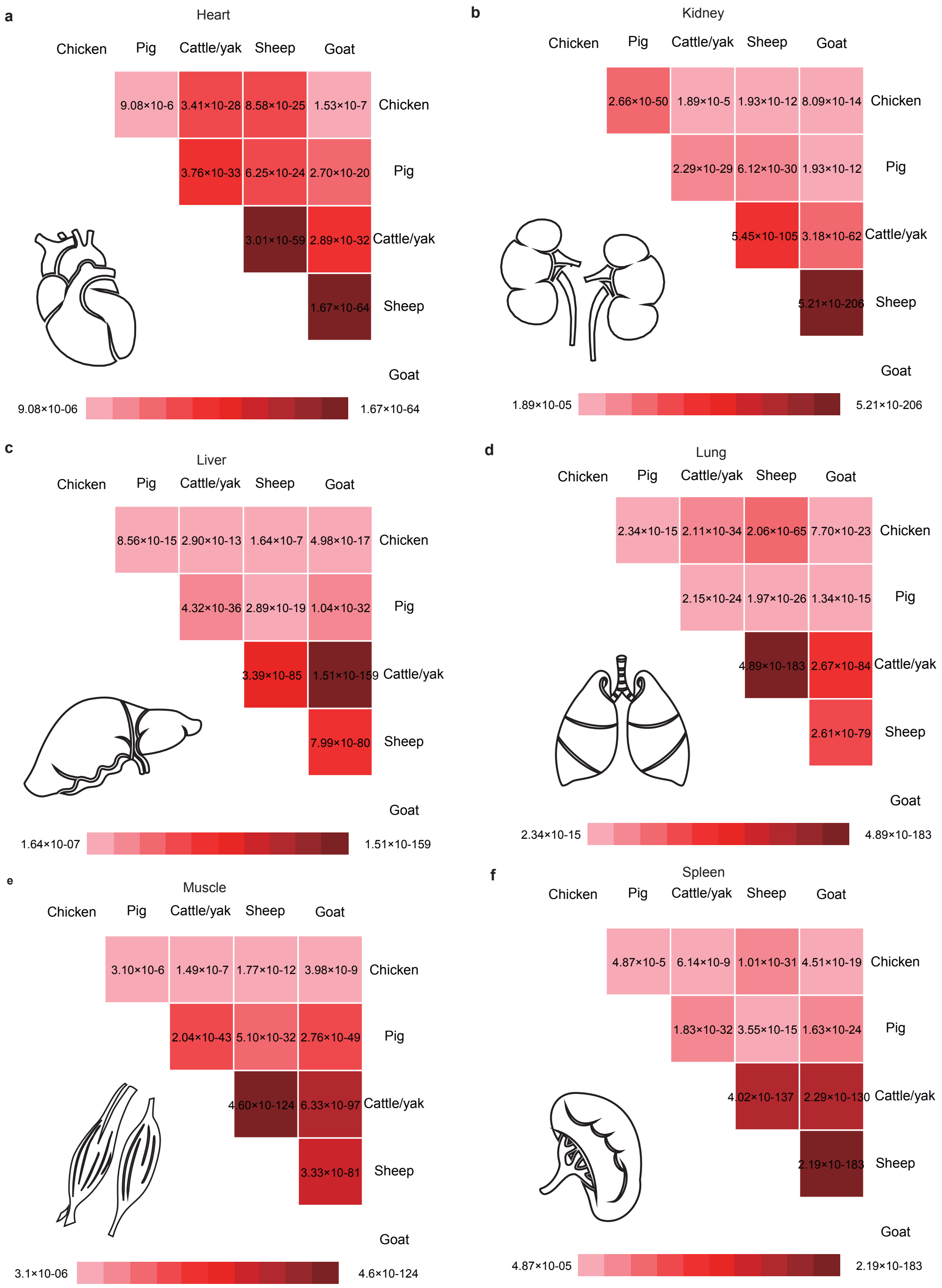

Supplemental Figure S10. DE genes from pair-wise comparison between altitudes were significantly shared among five vertebrates for each tissue. Significance of the overlapping DE genes was determined by Chi-square test or Fisher's exact test (P<4.87× 10^{-5}).

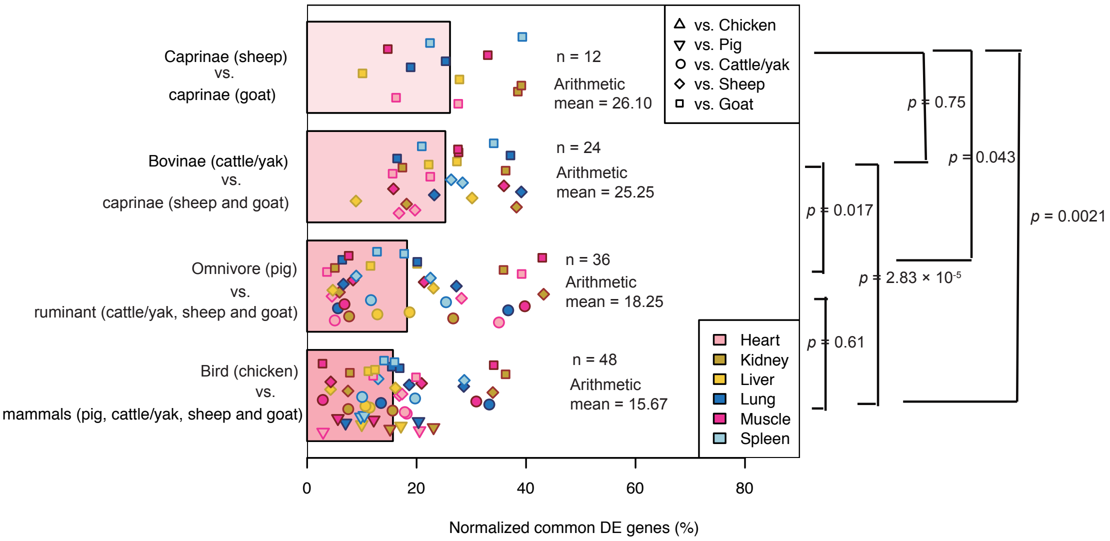

Supplementary Figure 11. More closely related vertebrates shared more common DE genes. To compare the similarity of DE genes in each tissue for each vertebrate, the number of shared DE genes in the pairwise comparison between the high- and low-altitude populations was normalized by the number of their respective DE genes in each tissue for each vertebrate. Statistical significance was determined by Wilcoxon rank sum test.

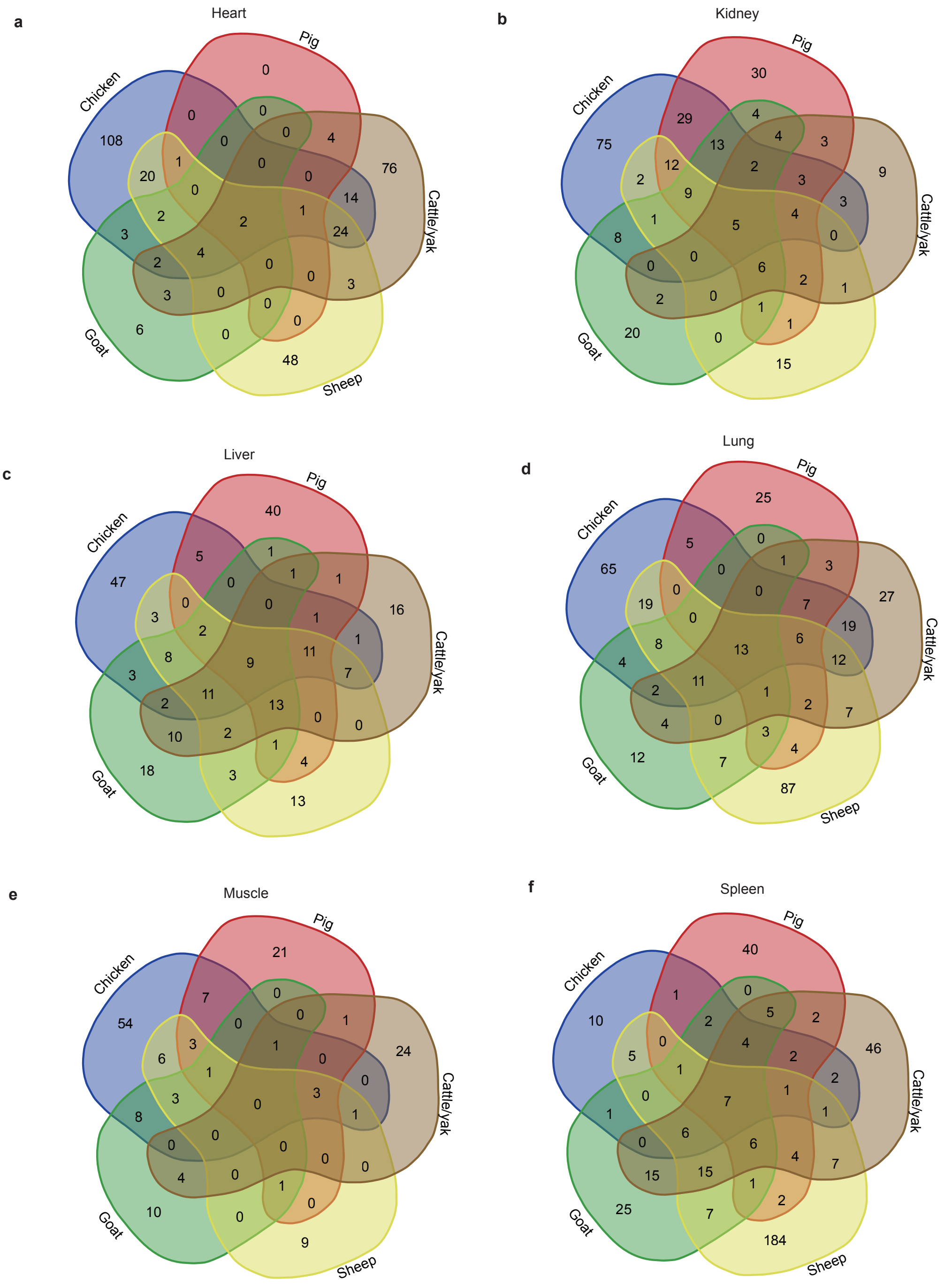

Supplemental Figure S12. Distribution of shared and specific enriched functional categories by DE genes between the high- and low-altitude populations in each tissue among five vertebrates.

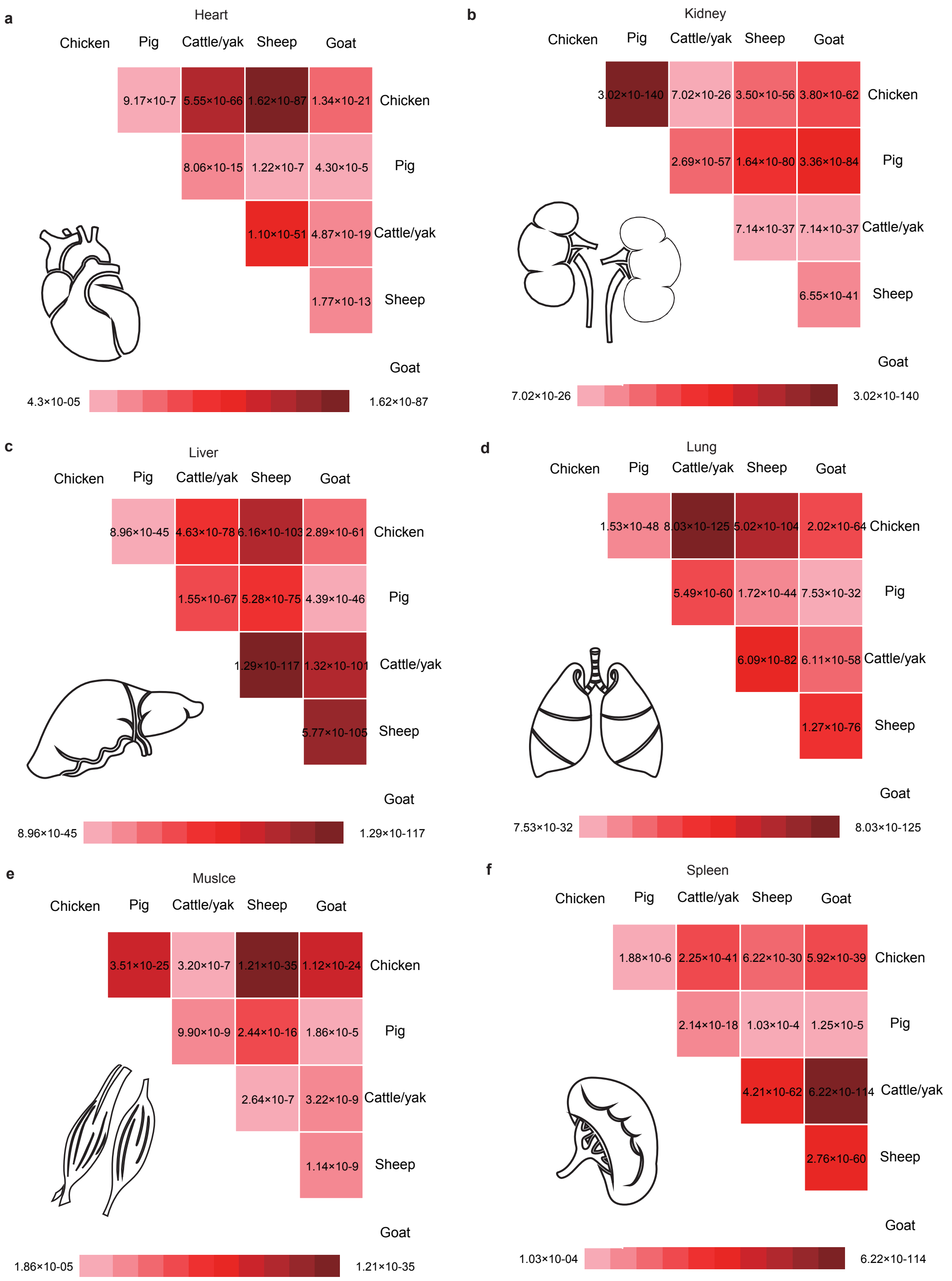

Supplemental Figure S13. Enriched functional categories of DE genes based on pair-wise comparison between altitudes were significantly shared among five vertebrates for each tissue. Significance of the overlapping enriched functional categories was determined by Chi-square test or Fisher's exact test (P<1.03 × 10^{-4}).

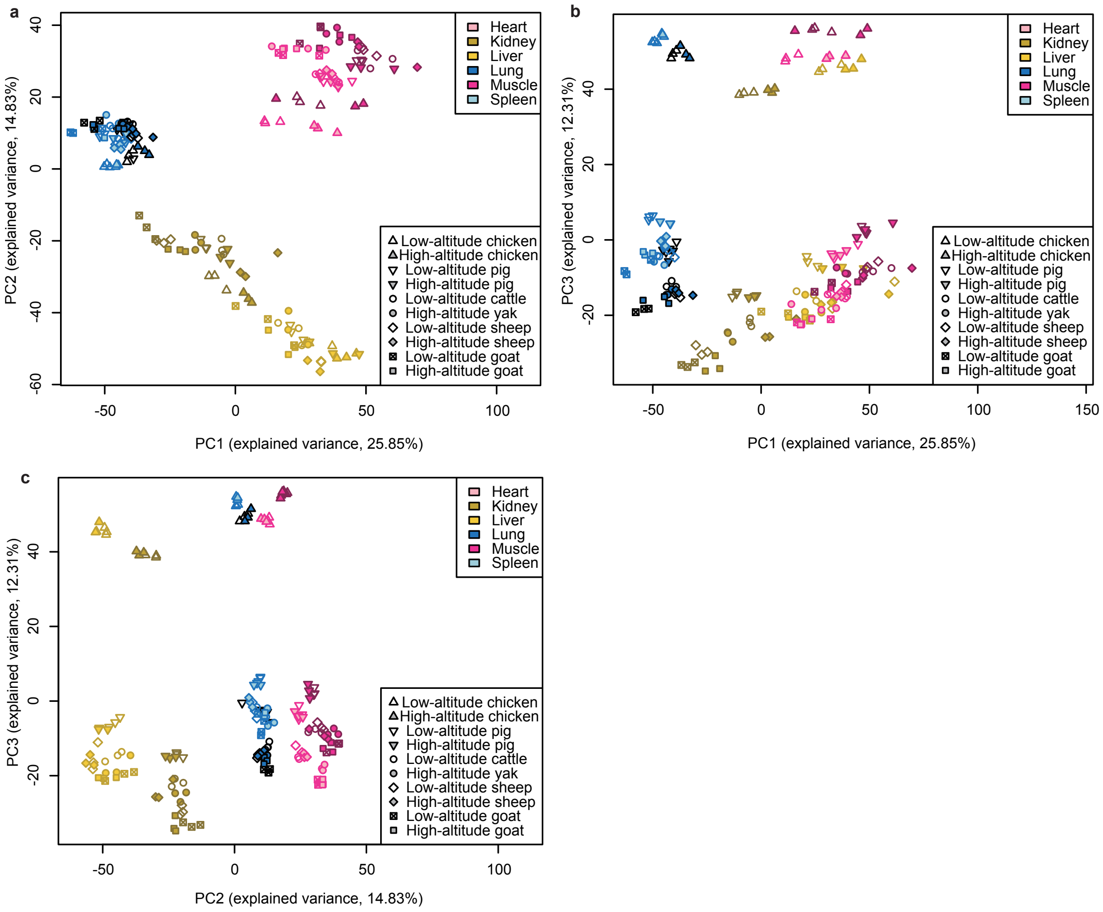

Supplementary Figure S14. Factorial map of the principal-component analysis (PCA) of gene expression levels for (a) first and second components (b) first and third components (c) second and third components.

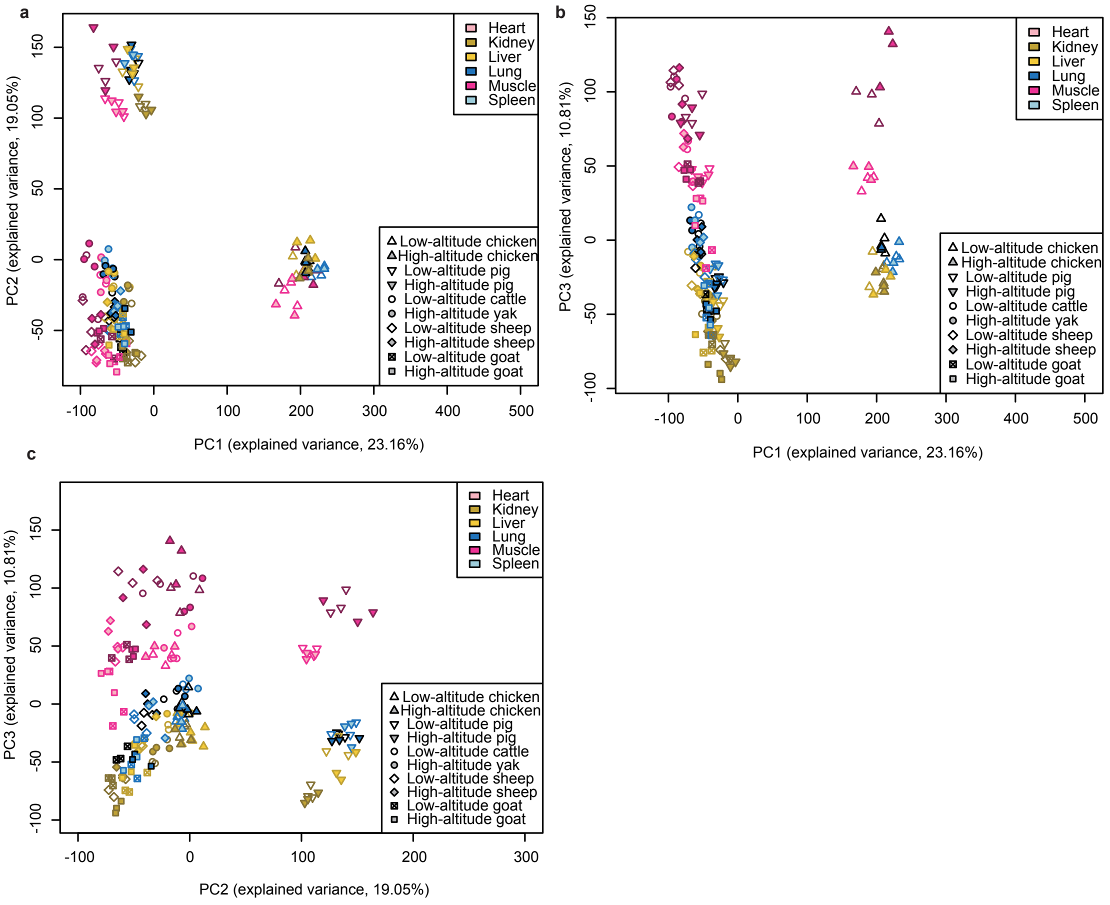

Supplementary Figure S15. Factorial map of the principal-component analysis (PCA) of the alternative splicing for (a) first and second components (b) first and third components (c) second and third components.

Supplementary Table S1. Summary of RNA-seq data.

Name	Individual	Tissue	Q20 (%)	Q30 (%)	GC(%)	Raw data (Gb)	High quality data (Gb)	Mapping ratio (%)
Low-altitude cattle	1	Skeletal muscle	96.64	90.00	83.35	5.23	4.98	81.68
		Lung	96.80	90.45	84.10	4.63	4.42	88.33
		Heart	97.27	91.53	85.79	4.49	4.26	76.23
		Liver	94.97	90.40	85.82	5.79	5.52	83.78
		Spleen	94.71	89.96	85.20	5.49	5.28	81.92
		Kidney	94.81	90.01	85.22	5.83	5.26	74.76
	2	Skeletal muscle	96.67	90.10	83.52	4.78	4.56	85.35
		Lung	96.68	90.13	83.59	5.17	4.96	87.40
		Heart	96.82	90.37	83.93	3.84	3.68	68.32
		Liver	94.76	90.05	85.34	6.19	5.94	85.09
		Spleen	94.65	89.85	85.05	5.18	4.94	82.04

		Kidney	94.39	89.47	84.55	5.08	4.92	74.89
	3	Skeletal muscle	96.75	90.28	83.80	4.46	4.26	77.79
		Lung	96.77	90.39	84.01	5.27	5.04	88.00
		Heart	96.90	90.63	84.36	5.46	5.22	68.50
		Liver	94.77	90.08	85.39	5.61	5.34	84.20
		Spleen	94.72	89.99	85.26	5.50	5.24	78.19
		Kidney	94.30	88.95	83.60	5.07	4.60	75.84
**High-altitude yak**	1	Skeletal muscle	96.93	90.69	84.44	4.89	4.64	83.37
		Lung	97.15	91.30	85.45	4.33	4.14	84.77
		Heart	97.88	93.08	88.28	5.30	5.12	66.86
		Liver	94.25	89.20	84.14	6.29	6.08	79.12
		Spleen	94.51	89.62	84.73	5.47	5.24	75.10
		Kidney	95.31	90.90	86.50	6.25	6.04	74.47

	2	Skeletal muscle	96.99	90.88	84.76	5.00	4.76	82.75
		Lung	97.17	91.36	85.54	4.27	4.10	84.74
		Heart	97.21	91.38	85.56	4.58	4.38	74.05
		Liver	94.81	90.14	85.47	5.37	5.14	78.35
		Spleen	94.63	89.83	85.03	5.90	5.66	75.29
		Kidney	95.32	90.93	86.54	6.36	6.16	75.05
	3	Skeletal muscle	97.13	91.20	85.28	4.64	4.44	83.51
		Lung	97.15	91.32	85.49	5.93	5.68	84.32
		Heart	97.23	91.45	85.66	4.21	4.02	75.35
		Liver	94.90	90.30	85.70	5.42	5.22	79.74
		Spleen	94.62	89.82	85.01	5.61	5.34	75.71
		Kidney	95.09	90.50	85.91	5.35	5.16	74.29
**Low-altitude goat**	1	Skeletal muscle	97.71	92.68	87.65	5.31	5.12	81.55

	Lung	97.59	92.54	87.48	4.48	4.28	84.22
	Heart	97.85	93.05	88.25	4.88	4.70	64.04
	Liver	94.35	89.26	84.16	5.94	5.74	81.05
	Spleen	93.89	88.49	83.08	5.62	5.38	77.42
	Kidney	95.28	90.91	86.53	7.45	7.22	50.54
2	Skeletal muscle	97.70	92.62	87.53	4.92	4.74	81.34
	Lung	97.53	92.32	87.12	5.36	5.16	84.10
	Heart	97.81	92.96	88.12	5.06	4.88	69.01
	Liver	94.23	89.05	83.86	5.89	5.62	80.19
	Spleen	93.92	88.52	83.12	6.12	5.84	78.63
	Kidney	95.12	90.61	86.10	7.13	6.94	50.16
3	Skeletal muscle	97.81	92.95	88.10	5.05	4.88	75.71
	Lung	97.58	92.47	87.35	5.35	5.14	85.93
	Heart	97.83	93.03	88.22	5.09	4.90	68.68

		Liver	94.18	88.94	83.71	5.37	5.16	81.18
		Spleen	94.02	88.72	83.41	5.37	5.14	78.34
		Kidney	94.83	90.09	85.34	5.58	5.40	52.37
**High-altitude goat**	1	Skeletal muscle	96.89	90.55	84.21	4.43	4.22	80.57
		Lung	97.47	92.08	86.70	4.71	4.52	86.85
		Heart	97.13	91.15	85.17	4.39	4.20	69.77
		Liver	93.97	88.60	83.23	5.62	5.40	81.15
		Spleen	94.09	88.81	83.53	5.84	5.58	76.67
		Kidney	93.65	87.85	82.04	6.12	5.92	72.96
	2	Skeletal muscle	96.13	88.64	81.16	5.82	5.42	81.38
		Lung	96.19	88.88	81.56	4.94	4.56	82.96
		Heart	97.14	91.14	85.15	5.06	4.76	72.26
		Liver	94.11	88.82	83.52	5.56	5.36	78.69
		Spleen	93.91	88.50	83.09	5.48	5.26	76.81

		Kidney	94.94	90.35	85.76	8.07	7.62	63.97
	3	Skeletal muscle	96.09	88.57	81.05	5.14	4.78	79.36
		Lung	96.20	88.90	81.60	4.85	4.52	81.64
		Heart	96.54	89.61	82.68	5.20	4.88	67.21
		Liver	94.20	88.98	83.75	5.58	5.34	80.03
		Spleen	94.09	88.81	83.54	5.69	5.36	75.69
		Kidney	93.76	88.02	82.28	6.06	5.88	73.54
**Low-altitude sheep**	1	Skeletal muscle	97.98	93.44	88.91	4.63	4.48	73.59
		Lung	97.06	91.25	85.45	4.35	4.16	83.51
		Heart	97.32	91.85	86.37	4.26	4.10	62.61
		Liver	94.02	88.58	83.14	5.25	4.76	73.56
		Spleen	94.31	89.29	84.28	6.22	5.54	75.63
		Kidney	95.28	90.86	86.44	6.67	6.44	56.64

	2	Skeletal muscle	96.79	90.34	83.88	5.14	4.90	81.13
		Lung	96.98	90.85	84.73	5.60	5.32	84.54
		Heart	97.06	91.00	84.93	5.54	5.36	69.92
		Liver	95.13	90.71	86.28	5.67	5.12	79.07
		Spleen	94.53	89.65	84.76	5.60	5.02	77.57
		Kidney	95.24	90.79	86.34	5.53	5.32	56.16
	3	Skeletal muscle	96.92	90.98	85.03	4.50	4.20	77.11
		Lung	97.04	90.96	84.89	4.73	4.54	84.32
		Heart	97.25	91.48	85.70	5.40	5.18	63.54
		Liver	95.01	90.46	85.91	5.60	5.06	79.81
		Spleen	94.50	89.57	84.65	5.57	5.06	76.78
		Kidney	95.13	90.63	86.13	5.15	4.98	53.14
**High-altitude sheep**	1	Skeletal muscle	97.02	91.13	85.23	4.03	3.84	79.36

	Lung	96.76	90.36	83.95	4.44	4.24	82.85
	Heart	96.95	90.76	84.58	4.47	4.30	68.55
	Liver	94.67	89.91	85.16	6.16	5.50	73.71
	Spleen	94.39	89.36	84.32	5.67	5.08	75.88
	Kidney	95.12	90.59	86.05	5.92	5.74	74.24
2	Skeletal muscle	96.89	90.78	84.67	5.92	5.64	78.84
	Lung	96.98	91.02	85.05	4.46	4.28	82.21
	Heart	97.27	91.72	86.17	6.42	6.18	67.90
	Liver	94.87	90.20	85.53	5.78	5.16	77.50
	Spleen	94.45	89.47	84.49	6.00	5.34	74.37
	Kidney	95.37	91.03	86.68	6.48	6.24	73.33
3	Skeletal muscle	97.03	91.17	85.31	5.19	4.96	80.08
	Lung	97.01	91.09	85.17	5.54	5.30	83.57
	Heart	97.21	91.57	85.93	4.88	4.68	73.63

		Liver	94.86	90.17	85.49	5.13	4.56	77.76
		Spleen	94.45	89.47	84.49	5.28	4.70	76.42
		Kidney	95.14	90.73	86.31	6.91	6.62	65.02
**Low-altitude pig**	1	Skeletal muscle	97.12	91.40	85.69	4.79	4.48	71.88
		Lung	97.36	91.95	86.53	4.79	4.58	76.16
		Heart	97.64	92.44	87.25	5.71	5.46	58.80
		Liver	94.41	89.38	84.34	5.18	4.58	75.01
		Spleen	94.15	88.92	83.70	5.63	4.90	69.44
		Kidney	93.67	87.93	82.18	5.21	4.98	70.85
	2	Skeletal muscle	95.56	87.28	79.00	4.29	3.96	71.72
		Lung	97.42	92.12	86.81	4.80	4.58	77.76
		Heart	97.67	92.52	87.38	5.02	4.80	55.12
		Liver	94.28	89.13	83.98	5.94	5.20	76.83

		Spleen	94.58	89.73	84.88	5.66	4.90	70.79
		Kidney	93.90	88.34	82.77	6.54	6.14	70.21
	3	Skeletal muscle	97.28	91.77	86.26	4.26	4.04	72.76
		Lung	97.27	91.71	86.15	4.59	4.40	72.02
		Heart	97.50	92.09	86.67	5.12	4.88	61.51
		Liver	94.56	89.62	84.68	6.42	5.60	76.21
		Spleen	94.12	88.87	83.61	5.63	4.94	68.85
		Kidney	93.93	88.59	83.24	5.18	4.74	69.54
**High-altitude pig**	1	Skeletal muscle	95.89	88.07	80.24	6.13	5.86	69.95
		Lung	97.36	91.91	86.45	4.80	4.58	77.89
		Heart	97.60	92.29	86.97	4.73	4.56	55.78
		Liver	94.61	89.74	84.88	5.00	4.38	75.01
		Spleen	94.13	88.85	83.56	5.40	4.82	67.98
		Kidney	94.76	90.24	85.71	7.19	6.98	66.36

	2	Skeletal muscle	95.83	87.92	80.00	4.45	4.22	67.43
		Lung	97.33	91.86	86.39	5.18	4.96	77.94
		Heart	97.66	92.51	87.36	5.65	5.40	56.42
		Liver	94.40	89.35	84.30	6.08	5.44	77.15
		Spleen	94.10	88.84	83.59	5.54	4.84	70.91
		Kidney	94.89	90.23	85.57	6.04	5.82	70.14
	3	Skeletal muscle	95.96	88.22	80.47	6.34	6.02	69.65
		Lung	97.46	92.17	86.88	4.31	4.12	77.90
		Heart	97.47	91.98	86.49	6.16	5.86	59.39
		Liver	94.44	89.43	84.41	5.07	4.40	75.52
		Spleen	94.61	89.78	84.94	5.66	5.02	69.44
		Kidney	93.84	88.19	82.53	4.77	4.60	68.98
**Low-altitude chicken**	1	Skeletal muscle	97.60	92.49	87.38	4.59	4.40	75.84
		Lung	97.55	92.57	87.59	5.04	4.80	84.25

	Heart	97.58	92.31	87.04	5.00	4.76	69.58
	Liver	95.11	90.71	86.32	6.72	6.06	81.51
	Spleen	94.80	90.26	85.71	5.55	5.00	77.91
	Kidney	93.61	87.93	82.26	5.28	4.94	74.08
2	Skeletal muscle	97.71	92.69	87.68	4.81	4.62	73.38
	Lung	97.51	92.47	87.43	4.77	4.54	84.24
	Heart	97.69	92.61	87.53	5.27	5.04	66.08
	Liver	94.91	90.37	85.84	5.31	4.82	79.51
	Spleen	94.68	90.06	85.43	5.18	4.66	76.47
	Kidney	94.37	89.44	84.52	5.51	5.12	74.38
3	Skeletal muscle	97.62	92.55	87.48	4.87	4.66	75.13
	Lung	97.55	92.58	87.60	4.79	4.56	83.24
	Heart	97.76	92.79	87.82	5.76	5.50	64.86
	Liver	95.13	90.73	86.32	5.62	5.04	78.86

		Spleen	94.67	90.03	85.39	6.23	5.58	78.21
		Kidney	93.46	87.64	81.83	5.88	5.64	71.29
**High-altitude chicken**	1	Skeletal muscle	97.61	92.48	87.35	4.84	4.64	76.27
		Lung	97.36	92.16	86.97	5.11	4.84	80.92
		Heart	97.58	92.21	86.83	4.75	4.52	57.48
		Liver	95.13	90.73	86.33	5.48	4.98	76.05
		Spleen	94.65	90.07	85.50	5.42	4.94	78.52
		Kidney	93.66	88.02	82.39	5.59	5.04	71.08
	2	Skeletal muscle	97.56	92.36	87.15	5.45	5.22	73.13
		Lung	97.40	92.24	87.07	5.07	4.82	82.68
		Heart	97.56	92.19	86.81	5.03	4.78	59.67
		Liver	95.06	90.60	86.15	6.10	5.56	78.48
		Spleen	94.58	89.92	85.27	5.30	4.80	74.00
		Kidney	93.72	88.39	83.07	6.18	5.54	70.57

	Skeletal muscle	97.60	92.45	87.30	4.84	4.64	72.53
	Lung	97.44	92.39	87.33	4.89	4.64	81.74
	Heart	97.54	92.12	86.70	4.67	4.42	58.56
3	Liver	94.93	90.37	85.81	5.65	5.10	76.30
	Spleen	94.29	89.41	84.52	5.17	4.68	76.45
	Kidney	94.86	90.30	85.74	6.69	6.20	72.86

The Illumina HiSeq 2000 with pair-end read of 100 nt in length used for heart, lung and muscle samples, and Illumina HiSeq 2500 with pair-end read of 125 nt in length used for kidney, liver and spleen samples.

Supplementary Table S2. Summary of whole-genome re-sequencing data.

	High-quality data (Gb)	Mapping ratio (%)	Depth coverage (×)	Coverage at least 1 × (%)	Coverage at least 4 × (%)
**Low-altitude pig**	37.57	92.01	13.29	98.4	93.9
**High-altitude pig**	55.15	92.25	19.56	98.57	95.02
**Low-altitude cattle**	34.53	99.23	10.77	98.08	93
**High-altitude yak**	36.73	99.21	11.49	96.77	91.4
**Low-altitude chicken**	12.5	98.12	11.23	96.72	92.85
**High-altitude chicken**	14.37	97.39	12.71	96.52	92.53
**Low-altitude sheep**	36.28	98.53	11.42	95.95	87.45
**High-altitude sheep**	30.97	98.24	10	96.32	88.52
**Low-altitude goat**	37.2	97.66	12.65	98.47	93.49
**High-altitude goat**	32.79	97.5	10.85	98.21	90.42

Supplementary Table S3. Percentage of genes with FPKM ≥ 0.5.

Animal	Tissue	Individual	Chicken(%)	Pig(%)	Cattle/yak(%)	Sheep(%)	Goat(%)
Low-altitude	Heart	1	66.33	57.02	62.11	58.46	49.08
		2	67.85	55.57	61.47	58.79	49.79
		3	68.20	57.56	61.64	58.24	49.62
	Kidney	1	72.58	64.33	64.82	63.26	55.50
		2	71.58	63.93	64.61	62.71	55.96
		3	71.71	63.37	64.62	63.69	54.85
	Liver	1	63.43	57.39	58.36	52.08	49.35
		2	61.90	56.34	61.58	56.17	49.37
		3	62.49	57.62	61.33	56.37	49.64
	Lung	1	76.57	66.77	68.29	67.10	57.10
		2	74.90	66.48	68.65	67.04	56.56
		3	76.06	65.57	68.46	66.96	55.88
	Muscle	1	70.09	55.64	57.29	53.74	48.45
		2	61.65	55.53	58.35	56.03	48.47
		3	64.93	56.37	59.02	56.62	48.44
	Spleen	1	73.46	63.17	66.34	63.19	54.20
		2	73.52	63.08	65.95	62.87	53.48
		3	74.22	62.18	64.18	63.86	52.94
High-altitude	Heart	1	71.38	56.49	59.04	58.01	51.40
		2	71.68	57.01	61.02	58.37	50.83
		3	70.27	58.14	63.29	57.38	50.80
	Kidney	1	75.28	62.16	64.97	60.24	54.73
		2	74.70	62.08	65.30	60.95	54.35
		3	73.26	62.79	66.48	59.17	53.89
	Liver	1	65.76	52.03	60.18	51.24	48.85
		2	66.15	56.10	60.78	55.46	49.56
		3	64.97	54.58	58.68	55.87	48.86
	Lung	1	75.51	66.18	67.72	66.67	57.19
		2	76.03	67.17	68.10	67.11	57.85
		3	78.05	66.63	68.77	66.22	58.11
	Muscle	1	69.20	53.65	58.08	56.07	49.47
		2	67.31	56.21	58.01	52.55	50.10
		3	68.97	57.52	60.12	55.51	48.76
	Spleen	1	74.19	61.45	65.16	61.29	54.20
		2	74.20	62.51	65.22	61.95	53.93
		3	74.51	62.66	65.66	61.08	53.07

Supplementary Methods

1. Calculation of 1:1 orthologues using Ensemble pipeline

We applied the most recent Ensemble pipeline (www.ensemble.org/info/genome/compara/homolog_method.html) to calculate 1:1 orthologues of five species. We downloaded the corresponding protein and CDS sequences of five species from Ensemble website with the exception of goat, whose protein and CDS sequences were downloaded from Goat Genome website. The sequences of an additional outgroup species zebrafish were also downloaded from Ensemble website. The longest protein sequence for each protein coding gene was kept for further analysis. Such protein sequences were concatenated to a single fasta file and makeblastdb function of NCBI blast+ version 2.2.28[1] was applied to generate the reference file. The merged protein sequence fasta file was blasted against the reference file using blastp function of NCBI blast+: in effect, each gene of six species were blasted against each other (both within and between species), using parameters -seg no -max_hsps_per_subject 1 -use_sw_tback -evalue 1e-10 -num_threads 1. Blast e-values were converted to weights based on MIN(100,ROUND(-LOG10(evalue)/2)), and Hcluster_sg (http://sourceforge.net/p/treesoft/code/HEAD/tree/) was utilized to cluster genes into families according to weights with parameters -m 750 -w 0 -s 0.34. Zebrafish was used as an outgroup species in this analysis by setting zebrafish genes to value 2 and non-zebrafish genes to value 1 in the category file, which was integrated into the analysis via –C option. Large clusters with more than 400 genes were recursively split into sub-clusters by QuickTree version 1.1 [2] until the largest sub-cluster contained less than 400 genes. In detail, multiple sequences of each large cluster were first aligned via Mafft version 7.149b [3] with parameter –auto and then converted to stockholm format by esl-reformat function in hmmer version 3.1b1 [4]. QuickTree were used to build unrooted tree and custom python scripts were utilized to find the branch that roughly split

the tree into two parts of comparable nodes, by making sure one of the two parts contained the smallest possible number of nodes over half of the total number. This splitting process was repeated until the largest of the final sub-clusters had less than 400 genes. The split clusters were combined with the original clusters with less than 400 genes. Multiple alignment of protein sequences for each cluster was then generated by Mafft if there were over 200 genes, or by a mixture of four aligners of mafftgins_msa, muscle_msa, kalign_msa and t_coffee_msa consensified by M-coffee version 10.00.r1613 [5] if otherwise. For each aligned cluster, we back-translated the protein sequences to CDS and applied TreeBeST (http://treesoft.sourceforge.net/treebest.shtml) to build phylogenetic trees reconciled with an inputted species tree. Custom python scripts were utilized to retrieve one-to-one orthologues.

2. Data filtering

I used prinseq-0.20.4 [6], cutadapt-1.12 [7] and in house developed script to perform the filtering. The parameters used are 'prinseq-lite.pl -fastq R1.fastq -fastq2 R2.fastq -out_format 3 -ns_max_p 10 -out_good output -out_bad null', and 'cutadapt -a AGATCGGAAGAGCACACGTCTGAACTCCAGTCAC --overlap=10 --error-rate=0.1 --discard-trimmed --paired-output tmp.2.fastq -o tmp.1.fastq R1_1.fastq R2_2.fastq', 'cutadapt -a AGATCGGAAGAGCGTCGTGTAGGGAAAGAGTGTAGATCTCGGTGGTCG CCGTATCATT --overlap=10 --error-rate=0.1 --discard-trimmed --paired-output result_1_filteradapt.fastq -o result_2_filteradapt.fastq tmp.2.fastq tmp.1.fastq'.

3. Mapping and variation detection parameters

The parameters for BWA mapping are 'bwa mem -t 10 -k 32 -M'. AddOrReplaceReadGroups and BuildBamIndex function in Picard version 1.14 (http://sourceforge.net/projects/picard/) was applied to add read group information and index, separately. Indel realignment was performed using RealignerTargetCreator and IndelRealigner tools in GATK. We called variants by HaplotypeCaller, separated SNVs and Indels using SelectVariants, filtered

SNVs with Fisher Strand values>60 or Qual By Depth values<2 or Mapping Quality values<40 or Mapping Quality Rank Sum Test values<-12.5 or Read Position Rank Sum Test values<-8, and filtered Indels with Fisher Strand values>200 or Qual By Depth values<2 or Read Position Rank Sum Test values<-20.

4. Tophat alignment parameters

Thank you for your valuable suggestions. The parameters we used are '--library-type fr-firststrand -p 4 --output-dir myoutputdir –G myspecies.gtf myspecies_genomeindex read1.fq.gz read2.fq.gz'.

1. Camacho C, Coulouris G, Avagyan V, Ma N, Papadopoulos J, Bealer K, et al. BLAST+: architecture and applications. BMC Bioinformatics. 2009;10:421. doi:10.1186/1471-2105-10-421.
2. Howe K, Bateman A and Durbin R. QuickTree: building huge Neighbour-Joining trees of protein sequences. Bioinformatics. 2002;18(11):1546-7.
3. Katoh K and Standley DM. MAFFT multiple sequence alignment software version 7: improvements in performance and usability. Mol Biol Evol. 2013;30(4):772-80. doi:10.1093/molbev/mst010.
4. Finn RD, Clements J and Eddy SR. HMMER web server: interactive sequence similarity searching. Nucleic Acids Res. 2011;39(Web Server issue):W29-37. doi:10.1093/nar/gkr367.
5. Wallace IM, O'Sullivan O, Higgins DG and Notredame C. M-Coffee: combining multiple sequence alignment methods with T-Coffee. Nucleic Acids Res. 2006;34 (6):1692-9. doi:10.1093/nar/gkl091.
6. Schmieder R and Edwards R. Quality control and preprocessing of metagenomic datasets. Bioinformatics. 2011;27(6):863-4. doi:10.1093/bioinformatics/btr026.
7. Martin M. Cutadapt removes adapter sequences from high-throughput sequencing reads. EMBnetJournal. 2011;17(1):10-2.
