# Supplementary material for: Comparative transcriptomics of 5 high-altitude vertebrates and their low-altitude relatives
Source: Gigascience. 2017 Nov 15;6(12):1–9. doi: 10.1093/gigascience/gix105 (PMC5729692; doi:10.1093/gigascience/gix105)
Supplement: Response-to-Reviewer-Comments_Original-Submission.pdf [file gix105_response-to-reviewer-comments_original-submission.pdf]

Author's Response To Reviewer Comments

Reviewer 1:

Comment 2-1
The authors report a well-developed project to better understand the gene expression differences in multiple tissues from 5 species (with the cattle-yak comparison counted as one). The data collected is enormous and clearly appears to be sufficient for the analyses proposed, but there are a number of questions regarding both the methods and results presented.

Response 2-1
Thank you for your positive comments. We would fully address your concerns and provide our point-to-point responses as follows.

Comment 2-2
Of highest importance, the authors present a set of analyses in which the output is a list of genes and a calculated expression level; these lists are then used in a number of ways to calculate expression and enriched function per tissue in several comparison. These lists (and not even the numbers of genes in each list) are not provided, so it is impossible to see these lists or use the lists as a resource for other work. Since one aspect of this publication would be as a resource for others, the authors must provide these lists as well as the calculated expression value for each gene. I realize these lists are extensive, but are a crucial component of the resource, especially for those readers who will not start with the raw data, but also for those who can repeat the analyses and compare their resulting normalized expression data with those that the authors created.

Response 2-2
Thank you for your reminder. According to the submission guidelines of GigaScience, we uploaded the complete gene lists with normalized expression values to the GigaScience temporary FTP server.

Comment 2-3
Further, the authors describe some biological results on comparisons between high and low altitude, but fail to provide sufficient description of the results. The Supplementary file is incomplete (see below), but also the text on all tissue and species comparisons is only a few sentences. More is needed to justify this reporting. For example, a strength of the work is the multi-species comparison of the same question of adaptation to high altitude. A comparison of the high/low differentially expressed gene lists in the same tissue across species would seem minimal and potentially very interesting- i.e., are the genes and pathways identified similar (more similar than expectation?). This would provide more insight, as well as more evidence the analyses are providing biologically relevant information.

Response 2-3
Thank you for your valuable suggestions. Based on your suggestions, we evaluated the amount of shared DE genes between the high- and low-altitude populations in each tissue among five vertebrates (Supplemental Figs. S9–10 and Additional File 3), and found that more closely

related vertebrates shared more common DE genes (Supplemental Fig. S11). We also discovered that the enriched functional categories of DE genes substantially overlapped (Supplemental Figs. S12–13 and Additional File 4). We added Supplemental Figs. S9–13 and Additional Files 3–4 to the manuscript.
As shown in the newly added Supplemental Figs. S9-13 and Additional Files 3-4, expectedly, the more closely related vertebrates (Fig. 1) shared more DE genes (Supplementary Figs. S9–10 and Additional File 3). Compared with shared DE genes among mammals, especially between the two closely related members of Caprinae (goat and sheep), the birds (chickens) exhibited significantly fewer shared DE genes with mammals (Wilcoxon rank sum test, $P<0.0021$) (Supplementary Fig. S11). We also identified significantly enriched functional gene categories of DE genes (Chi-square test or Fisher's exact test, $P<1.03 \times 10-4$), which were shared among multiple pairwise comparisons (Supplementary Figs. S12–13 and Additional File 4), that were potentially related to the dramatic phenotypic changes shaped by high-altitude adaptation, such as response to hypoxia (typically, 'oxidation reduction', 'heme binding', 'oxygen binding' , 'oxygen transport' and 'oxygen transporter activity'), cardiovascular system ('angiogenesis' and 'positive regulation of angiogenesis'), the efficiency of biomass production in the resource-poor highland ('metabolic pathways', 'cholesterol biosynthetic process' and 'steroid metabolic process') as well as immune response ('responses of immune and defense') (Additional file 2) (the statement has been added to the main text, page 11, line 251-267).

Comment 2-4
1. Criterion for expression.
a) On line 40, the authors indicate they are using a FPKM of 0.1. I was unable to find specific details on the sequencing data so that I could determine the number of counts this represents. I could not find the read length nor whether this was SE or PE. Assuming 100 nt read length and PE for the average of 5 Gb for each tissue reported, a FPKM of 0.1 is 2.5 counts for a 1 kb transcript. This is very low. The authors should justify this low cutoff, which affects all subsequent analyses. I would like to see the median expression level for each tissue, as well.

Response 2-4
Thank you for your valuable suggestions. Our data are paired-end reads of 100 nt for three tissues (heart, lung, and muscle), and 125 nt for the other three tissues (kidney, liver, and spleen). Although some previous reports used FPKM >0.1 as the cutoff for transcribed genes [1-3], based on your suggestions, we used a stricter cut-off of FPKM>0.5 (>0.5 FPKM for over 80% of the samples) in the subsequent analyses and updated all of the figures and tables. Our findings did not conflict with those in the initial manuscript, and were further strengthened, typically the 3D PCA result: chickens formed a distinct cluster from the mammals, which indicates that divergence in gene expression among these species started to surpass that between different tissues around when birds diverged from mammals (approximately 300 million years). We revised the corresponding text from "The exceptions to tissue dominance were that chicken heart, lung and liver clustered with chicken skeletal muscle, spleen and kidney, respectively, rather than with their mammalian counterparts, which implied that divergence in gene expression among these species started to surpass those between different tissues at about the time when birds split from mammals (~300 million years)" to "Notably, tissues of birds (chickens) formed a distinct cluster, rather than with their mammalian counterparts, which indicates that divergence in gene expression among these species started to surpass that between different tissues around

when birds diverged from mammals (approximately 300 million years ago).” (Main text, page 10, lines 232-236). After adding the FPKM 0.5 cut-off filtering for genes and 5 as the gene number cut-off for enriched terms, some of the specific over-represented terms changed even though the enriched general categories remained unchanged. We have revised the corresponding text from “As expected, respectable significantly enriched functional gene categories by DGEs, which shared in multiple pair-wise comparisons, were potentially related to the dramatic phenotypic changes shaped by high-altitude adaptation, such as response to hypoxia (typically, ‘oxidation reduction’, ‘heme binding’, ‘oxygen binding’ , ‘response to oxygen levels’ and ‘response to hypoxia’), cardiovascular system (‘blood vessel development’, ‘blood vessel morphogenesis’, ‘blood circulation’ and ‘development of lung and heart’), the efficiency of biomass production in the resource-poor highland (processes of ‘steroid biosynthesis’ and ‘fatty acid metabolism’) as well as immune response (‘responses of immune and defense’)” to “Expectedly, the more closely related vertebrates (Fig. 1) shared more DE genes (Supplementary Figs. S9–10 and Additional File 3). Compared with shared DE genes among mammals, especially between the two closely related members of Caprinae (goat and sheep), the birds (chickens) exhibited significantly fewer shared DE genes with mammals (Wilcoxon rank sum test, P<0.0021) (Supplementary Fig. S11). We also identified significantly enriched functional gene categories of DE genes (Chi-square test or Fisher’s exact test, P<1.03 × 10-4), which were shared among multiple pairwise comparisons (Supplementary Figs. S12–13 and Additional File 4), that were potentially related to the dramatic phenotypic changes shaped by high-altitude adaptation, such as response to hypoxia (typically, ‘oxidation reduction’, ‘heme binding’, ‘oxygen binding’ , ‘oxygen transport’ and ‘oxygen transporter activity’), cardiovascular system (‘angiogenesis’ and ‘positive regulation of angiogenesis’), the efficiency of biomass production in the resource-poor highland (‘metabolic pathways’, ‘cholesterol biosynthetic process’ and ‘steroid metabolic process’) as well as immune response (‘responses of immune and defense’) (Additional file 2).” (Main text, page 11, lines 251-267). We also revised the corresponding text from “Of these, ~75% reads could be aligned to their respective reference genomes, and on average ~70% of annotated protein coding genes in each organism showed FPKM expression values greater than 0.1” to “Of these, ~75% reads could be aligned to their respective reference genomes, and on average ~60% of annotated protein coding genes in each organism showed FPKM expression values greater than 0.5” (Main text, page 2, lines 40-41); from “Log2-transformed values of (FPKM + 1) for genes were used in subsequent analyses” to “Log2-transformed values of (FPKM + 1) for genes with >0.5 FPKM in over 80% of the samples were used in subsequent analyses” (Main text, page 5, lines 113-114); from “We found that on average 69.7% annotated protein coding genes in each genome had FPKM expression values greater than 0.1” to “We found that on average 61.2% annotated protein coding genes in each genome had FPKM expression values greater than 0.5” (Main text, page 8, lines 181-183); from “The gene expression-based tree based 7,125 single-copy orthologous genes for each tissue showed a highly consistent topology to the nucleotide sequence alignment-based phylogeny” to “The gene expression-based tree based 4,746 transcribed single-copy orthologous genes (66.61% of 7125) for each tissue showed a highly consistent topology to the nucleotide sequence alignment-based phylogeny (Fig. 2, Supplementary Methods) [9]” (Main text, page 8, lines 189-192); from “Through comparison of expression levels of 7,125 single-copy orthologous genes” to “Through comparison of expression levels of 4,746 transcribed single-copy orthologous genes” (Main text, page 9, lines 200-201); from “For gene expression, there were critical biological differences among tissues (Pearson’s r = 0.71 and weighted average proportion

variance = 0.42), followed by species (Pearson's r = 0.84, weighted average proportion variance = 0.16) and local adaptation (Pearson's r = 0.97 and weighted average proportion variance = 0.019)" to "For gene expression, there were critical biological differences among tissues (Pearson's r = 0.67 and weighted average proportion variance = 0.36), followed by species (Pearson's r = 0.75, weighted average proportion variance = 0.22) and local adaptation (Pearson's r = 0.95 and weighted average proportion variance = 0.019)" (Main text, page 9, lines 206-210); from "We identified ~1,512 DEGs between 30 low- versus high-altitude pairs (225 DEGs in liver of pigs to 4,014 DEGs in kidney of sheep) (Table 1). Notably, among five pairs of vertebrate, the highly-diverged yak and cattle exhibited the highest number of DEG (~2,242) across six tissues. Among six tissues, the highly aerobic kidney exhibited the highest number of DEGs (~2,103) across five pairs of vertebrates." to "We identified ~1,423 DEGs between 30 low- versus high-altitude pairs (177 DEGs in muscle of chickens to 3,853 DEGs in kidney of sheep) (Table 1). Notably, among five pairs of vertebrate, the highly-diverged yak and cattle exhibited the highest number of DEG (~2,005) across six tissues. Among six tissues, the highly aerobic kidney exhibited the highest number of DEGs (~2,097) across five pairs of vertebrates" (Main text, page 11, lines 245-250).
The median of gene expression values (reflected by FPKM values) increased from 6.86 to 8.65, which corresponds to the increase of filtering cut-offs from 0.1 to 0.5 (Table R1 can be accessed from RL_FiguresandTables.pdf at:
https://www.dropbox.com/s/shgpb4784s409zw/RL_FiguresandTables.pdf?dl=0).

Comment 2-5
b) On line 188, the authors use the term "high confidence single-copy orthologs" this is not defined. And is this homology based or expression based?

Response 2-5
Thank you for your valuable suggestions. We are sorry for our descriptive statement of approaches. We adopted the Ensemble pipeline that is more accurate than more feasible OrthMCL method:
We applied the most recent Ensemble pipeline (www.ensemble.org/info/genome/compara/homolog_method.html) to calculate 1:1 orthologues of five species. We downloaded the corresponding protein and CDS sequences of five species from Ensemble website with the exception of goat, whose protein and CDS sequences were downloaded from Goat Genome website. The sequences of an additional outgroup species zebrafish were also downloaded from Ensemble website. The longest protein sequence for each protein coding gene was kept for further analysis. Such protein sequences were concatenated to a single fasta file and makeblastdb function of NCBI blast+ version 2.2.28 [4] was applied to generate the reference file. The concatenated protein sequence fasta file was blasted against the reference file using blastp function of NCBI blast+: in effect, each gene of six species were blasted against each other (both within and between species), using parameters -seg no -max_hsps_per_subject 1 -use_sw_tback -evalue 1e-10 -num_threads 1. Blast e-values were converted to weights based on MIN(100,ROUND (-LOG10(evalue)/2)), and Hcluster_sg (http://sourceforge.net/p/treesoft/code/HEAD/tree/) was utilized to cluster genes into families according to weights with parameters -m 750 -w 0 -s 0.34. Zebrafish was used as an outgroup species in this analysis by setting zebrafish genes to value 2 and non-zebrafish genes to value 1 in the category file, which was integrated into the analysis via

–C option. Large clusters with more than 400 genes were recursively split into sub-clusters by QuickTree version 1.1 [5] until the largest sub-cluster contained less than 400 genes. In detail, multiple sequences of each large cluster were first aligned via Mafft version 7.149b [6] with parameter –auto and then converted to stockholm format by esl-reformat function in hmmer version 3.1b1 [7]. QuickTree were used to build unrooted tree and custom python scripts were utilized to find the branch that roughly split the tree into two parts of comparable nodes, by making sure one of the two parts contained the smallest possible number of nodes over half of the total number. This splitting process was repeated until the largest of the final sub-clusters had less than 400 genes. The split clusters were combined with the original clusters with less than 400 genes. Multiple alignment of protein sequences for each cluster was then generated by Mafft if there were over 200 genes, or by a mixture of four aligners of mafftgins_msa, muscle_msa, kalign_msa and t_coffee_msa consensified of M-coffee version 10.00.r1613 [8] if otherwise. For each aligned cluster, we back-translated the protein sequences to CDS and applied TreeBeST (http://treesoft.sourceforge.net/treebest.shtml) to build phylogenetic trees reconciled with an inputted species tree. Custom python scripts were utilized to retrieve one-to-one orthologues. We also added the detailed method to the Supplementary Methods, hoping such information will help readers better understand our work.

Comment 2-6
2. Comparison of expression differences between high and low altitude animals and functional annotation analysis.
a) Supplemental Figure S3 shows that in some tissues there are large differences in mapping rate that are not reflected in the other altitude type. Did the authors check that mapping rate did not affect their differential expression calls? Also, please report the tissue type in this graph.

Response 2-6
As you suggested, we redrew the figures and compared the mapping ratios between low- and high-altitude populations for each vertebrate. Interestingly, we found that populations with a relatively lower mapping ratio of RNA-seq data had relatively higher genomic divergence from the reference genome (which was reflected by more SNPs based on whole-genome sequence data), and vice versa (Supplementary Fig. S3).
Thank you for pointing out that several tissues exhibited relatively lower mapping ratios. For example, hearts of high- and low-altitude pigs (Illumina HiSeq 2000 with 100-nt paired-end reads) and kidneys of low-altitude goats (Illumina HiSeq 2500 with 125-nt paired-end reads) (Supplementary Fig. S3) exhibited the lowest mapping ratios. This result indicated that the relatively lower mapping ratios may not be attributed to the idiosyncrasies of the different sequencing platforms.
We then considered that the discrepancies in mapping ratios might be attributable to bias from library construction, which can be effectively corrected during the normalization steps implemented in cuffdiff [9]: to correct for library sizes (i.e., sequencing depths), FPKMs and fragment counts are scaled via the median of the geometric means of fragment counts across all libraries, as described by Anders and Huber [10].

Comment 2-7
b) In Additional File 2, a large table provided the GO/KEGG/InterPro terms and whether lists of genes with specific difference in high/low altitude expression are significantly enriched for that

term. The authors should show the number of genes in the list for each comparison, or only show those with at least 5-10 genes in a list. Low representation in a pathway or term can be misleading for enrichment.

Response 2-7
Thank you for your valuable suggestions. We compared the similarities and differences of DE genes and their enriched categories between high altitude vertebrates and their low-altitude relatives within each tissue for each species (Supplementary Figs. S9-13, Additional Files 3-4). Then we retained gene lists with at least 5 genes, and updated all the relevant figure and tables accordingly.

Comment 2-8
c) More importantly, the authors do not indicate the background used for these analyses. It would be most appropriate to use the total number of genes expressed in each tissue for such analyses, so that the background reflects the genes that could possibly be shown to be differentially expressed, not the genome-wide background which is often the default.

Response 2-8
Thank you for your valuable comment. As previously reported [11-19], we used the annotated genes of whole-genome as the background for gene functional enrichment analysis in our initial submission. However, as you noted, it is more appropriate to use the genes expressed in each tissue as the background for gene functional enrichment analysis, which is more representative and could prevent the potential bias of over-representation of the tissue-specific expressed genes [20]. Based on your suggestion, we re-performed gene functional enrichment analysis by using ONLY the transcribed genes as the background, and found that the updated results were consistent with our initial results (Supplementary Figs. 12–13 and Additional Files 2, 4).

Reviewer 2:

Comment 3-1
First of all let me congratulate you and all authors for this piece of research. I have although some questions that I believe are important in order to improve your manuscript:
In Data Analysis:

Response 3-1
Thank you so much for your positive comments.

Comment 3-2
page 4, lines 85-88: may you specify how the data filtering was performed? which software did you use, or in case you have used in house developed scripts may you please provide them as supplemental information?

Response 3-2
Thank you so much for your questions. I used prinseq-0.20.4 [21], cutadapt-1.12 [22] and in house developed script to perform the filtering. The parameters used are ‘prinseq-lite.pl -fastq R1.fastq -fastq2 R2.fastq -out_format 3 -ns_max_p 10 -out_good output -out_bad null’, and

‘cutadapt -a AGATCGGAAGAGCACACGTCTGAACTCCAGTCAC --overlap=10 --error-rate=0.1 --discard-trimmed --paired-output tmp.2.fastq -o tmp.1.fastq R1_1.fastq R2_2.fastq’, ‘cutadapt -a AGATCGGAAGAGCGTCGTGTAGGGAAAGAGTGTAGATCTCGGTGGTCGCCGTATCATT --overlap=10 --error-rate=0.1 --discard-trimmed --paired-output result_1_filteradapt.fastq -o result_2_filteradapt.fastq tmp.2.fastq tmp.1.fastq’ (Supplementary Methods).

Comment 3-3
page 4 line 93, may you specify the parameters used for the analysis performed with EnsemblComparaGeneTrees method?

Response 3-3
Thank you for your valuable suggestions. We applied the most recent Ensemble pipeline (www.ensemble.org/info/genome/compara/homolog_method.html) to calculate 1:1 orthologues of five species. We downloaded the corresponding protein and CDS sequences of five species from Ensemble website with the exception of goat, whose protein and CDS sequences were downloaded from Goat Genome website. The sequences of an additional outgroup species zebrafish were also downloaded from Ensemble website. The longest protein sequence for each protein coding gene was kept for further analysis. Such protein sequences were concatenated to a single fasta file and makeblastdb function of NCBI blast+ version 2.2.28[4] was applied to generate the reference file. The merged protein sequence fasta file was blasted against the reference file using blastp function of NCBI blast+: in effect, each gene of six species were blasted against each other (both within and between species), using parameters -seg no -max_hsps_per_subject 1 -use_sw_tback -evalue 1e-10 -num_threads 1. Blast e-values were converted to weights based on MIN(100,ROUND (-LOG10(evalue)/2)), and Hcluster_sg (http://sourceforge.net/p/treesoft/code/HEAD/tree/) was utilized to cluster genes into families according to weights with parameters -m 750 -w 0 -s 0.34. Zebrafish was used as an outgroup species in this analysis by setting zebrafish genes to value 2 and non-zebrafish genes to value 1 in the category file, which was integrated into the analysis via –C option. Large clusters with more than 400 genes were recursively split into sub-clusters by QuickTree version 1.1 [5] until the largest sub-cluster contained less than 400 genes. In detail, multiple sequences of each large cluster were first aligned via Mafft version 7.149b [6] with parameter –auto and then converted to stockholm format by esl-reformat function in hmmer version 3.1b1 [7]. QuickTree were used to build unrooted tree and custom python scripts were utilized to find the branch that roughly split the tree into two parts of comparable nodes, by making sure one of the two parts contained the smallest possible number of nodes over half of the total number. This splitting process was repeated until the largest of the final sub-clusters had less than 400 genes. The split clusters were combined with the original clusters with less than 400 genes. Multiple alignment of protein sequences for each cluster was then generated by Mafft if there were over 200 genes, or by a mixture of four aligners of mafftgins_msa, muscle_msa, kalign_msa and t_coffee_msa consensified by M-coffee version 10.00.r1613 [8] if otherwise. For each aligned cluster, we back-translated the protein sequences to CDS and applied TreeBeST (http://treesoft.sourceforge.net/treebest.shtml) to build phylogenetic trees reconciled with an inputted species tree. Custom python scripts were utilized to retrieve one-to-one orthologues (Supplementary Methods).

Comment 3-4
page 4- line 96, may you please detail the parameters used for the BWA alignment?

Response 3-4
Thank you for the valuable suggestions. The parameters are ‘bwa mem -t 10 -k 32 -M’ (Supplementary Methods).

Comment 3-5
page 5- line 101- which were the parameters defined for GATK detection of SNPs and Indels? Parameters like Calling confidence and minimum read depth?

Response 3-5
Thank you for your valuable suggestions. AddOrReplaceReadGroups and BuildBamIndex function in Picard version 1.14 (http://sourceforge.net/projects/picard/) was applied to add read group information and index, separately. Indel realignment was performed using RealignerTargetCreator and IndelRealigner tools in GATK. We called variants by HaplotypeCaller, separated SNVs and Indels using SelectVariants, filtered SNVs with Fisher Strand values>60 or Qual By Depth values<2 or Mapping Quality values<40 or Mapping Quality Rank Sum Test values<-12.5 or Read Position Rank Sum Test values<-8, and filtered Indels with Fisher Strand values>200 or Qual By Depth values<2 or Read Position Rank Sum Test values<-20 (Supplementary Methods).

Comment 3-6
page 5 line 108- which parameters were used for the TopHat alignment?

Response 3-6
Thank you for your valuable suggestions. The parameters we used are ‘--library-type fr-firststrand -p 4 --output-dir myoutputdir –G myspecies.gtf myspecies_genomeindex read1.fq.gz read2.fq.gz’ (Supplementary Methods).

Comment 3-7
In Findings:
I am missing analysis that I was expecting in a study of adaptation to altitude which generated so much WGS data. I suggest that you study genetic divergence by Fst or by Tajima's D and make identification of selection footprints. It would be great then to compare the genes being harbored in selective sweeps and the changes at transcriptomic level.

Response 3-7
We greatly appreciate your valuable comments.
At present, few studies have sufficiently characterized the direct relationship between genes embedded in selected regions and expression changes. Consequently, exploring the potential impact of positive selection on gene transcription is of great interest. As far as we know, only three vertebrates have publicly available whole-genome sequences for multiple individuals of both low-altitude populations (Pengxian chickens, Rongchang pigs, and Jersey cattle) and their high-altitude relatives (Tibetan chickens, Tibetan pigs, and yak) [23-26] (Table R2 can be accessed from RL_FiguresandTables.pdf at:

https://www.dropbox.com/s/shgpb4784s409zw/RL_FiguresandTables.pdf?dl=0).
To investigate the effects of positive selection on gene expression, we downloaded the above datasets and identified the genes embedded in selected regions (see Fig. R1) for high-altitude populations (Tibetan chickens, Tibetan pigs, and yak) against their low-altitude relatives (Pengxian chickens, Rongchang pigs, and Jersey cattle) (see Figs. R2–4) (Figs. R1-4 can be can be accessed from RL_FiguresandTables.pdf at:
https://www.dropbox.com/s/shgpb4784s409zw/RL_FiguresandTables.pdf?dl=0).
We found the genes embedded in selected regions exhibited highly comparable expression levels between the high-altitude populations and their low-altitude relatives within each tissue for each vertebrate, which was similar (P values of Wilcoxon rank sum test range from 0.120 to 0.939) to the genes outside selected regions (see Fig. R2).
We further observed expression levels of genes embedded in selected regions are highly comparable with the genes outside selected regions within each tissue for high-altitude population of each vertebrate (P values of Wilcoxon rank sum test range from 0.297 to 0.934) (see Fig. R3), this tendency also exists in their respective low altitude relatives (P values of Wilcoxon rank sum test range from 0.346 to 0.940) (see Fig. R4).
In this study, we did not observe the effects of positive selection on gene expression, which was most likely due to the distinct functional roles of variations with highly skewed frequency spectra. Generally, SNPs can be classified as coding (synonymous, missense, and nonsense) and non-coding. It is essential to perform further functional analyses to assess the impact of variations on gene expression; it is especially necessary to decipher the impact of non-coding variations that are located in regulatory regions (in particular, promoters, enhancers, and silencers) on gene expression.
Additionally, it is worth noting that our investigation is based on different individuals and had a small sample size; further large-scale experiments with proper design would be beneficial for answering this question.

Comment 3-8
page 10 lines 230-235: Did this happen in the low altitude chicken or only in one? its hard to see this in the figure

Response 3-8
Thank you for your thoughtful comment. As shown in the updated Fig. 4a and 4b (see Response 2–4), the Tibetan chickens and their low-altitude relatives formed a distinct cluster from the mammals. We revised this part of the manuscript to: "Notably, tissues of birds (chickens) formed a distinct cluster, rather than with their mammalian counterparts, which indicates that divergence in gene expression among these species started to surpass that between different tissues around when birds diverged from mammals (approximately 300 million years ago)." (Figs. 4a and 4b)

Comment 3-9
page 11 lines 251-259: The way these results are presented its hard to infer if the pathways affected by adaptation to altitude if these were the same between species or not. This is an important question that your results would enable to answer. I would suggest that a table per species should be made as well as venn diagrams that would lead us to understand which pathways were commonly affected or were different between species and if these were the same also at tissue level. I would like to see this part of the manuscript more enhanced, giving a larger

value to the high value data that you have generated in your research.

Response 3-9
Thank you for your valuable suggestions, which are also commented by reviewer 1 (please see Response 2-3 as follows).
Thank you for your valuable suggestions. Based on your suggestions, we evaluated the amount of shared DE genes between the high- and low-altitude populations in each tissue among five vertebrates (Supplemental Figs. S9–10 and Additional File 3), and found that more closely related vertebrates shared more common DE genes (Supplemental Fig. S11). We also discovered that the enriched functional categories of DE genes substantially overlapped (Supplemental Figs. S12–13 and Additional File 4). We added Supplemental Figs. S9–13 and Additional Files 3–4 to the manuscript.
As shown in the newly added Supplemental Figs. S9-13 and Additional Files 3-4, expectedly, the more closely related vertebrates (Fig. 1) shared more DE genes (Supplementary Figs. S9–10 and Additional File 3). Compared with shared DE genes among mammals, especially between the two closely related members of Caprinae (goat and sheep), the birds (chickens) exhibited significantly fewer shared DE genes with mammals (Wilcoxon rank sum test, $P<0.0021$) (Supplementary Fig. S11). We also identified significantly enriched functional gene categories of DE genes (Chi-square test or Fisher's exact test, $P<1.03 \times 10-4$), which were shared among multiple pairwise comparisons (Supplementary Figs. S12–13 and Additional File 4), that were potentially related to the dramatic phenotypic changes shaped by high-altitude adaptation, such as response to hypoxia (typically, 'oxidation reduction', 'heme binding', 'oxygen binding' , 'oxygen transport' and 'oxygen transporter activity'), cardiovascular system ('angiogenesis' and 'positive regulation of angiogenesis'), the efficiency of biomass production in the resource-poor highland ('metabolic pathways', 'cholesterol biosynthetic process' and 'steroid metabolic process') as well as immune response ('responses of immune and defense') (Additional file 2) (the statement has been added to the main text, page 11, line 251-267).

1. Brooks MJ, Rajasimha HK, Roger JE and Swaroop A. Next-generation sequencing facilitates quantitative analysis of wild-type and Nrl(-/-) retinal transcriptomes. Mol Vis. 2011;17:3034-54.
2. Hugo W, Shi H, Sun L, Piva M, Song C, Kong X, et al. Non-genomic and Immune Evolution of Melanoma Acquiring MAPKi Resistance. Cell. 2015;162(6):1271-85. doi:10.1016/j.cell.2015.07.061.
3. Mele M, Ferreira PG, Reverter F, DeLuca DS, Monlong J, Sammeth M, et al. Human genomics. The human transcriptome across tissues and individuals. Science. 2015;348(6235):660-5. doi:10.1126/science.aaa0355.
4. Camacho C, Coulouris G, Avagyan V, Ma N, Papadopoulos J, Bealer K, et al. BLAST+: architecture and applications. BMC Bioinformatics. 2009;10:421. doi:10.1186/1471-2105-10-421.
5. Howe K, Bateman A and Durbin R. QuickTree: building huge Neighbour-Joining trees of protein sequences. Bioinformatics. 2002;18(11):1546-7.
6. Katoh K and Standley DM. MAFFT multiple sequence alignment software version 7: improvements in performance and usability. Mol Biol Evol. 2013;30(4):772-80. doi:10.1093/molbev/mst010.
7. Finn RD, Clements J and Eddy SR. HMMER web server: interactive sequence similarity searching. Nucleic Acids Res. 2011;39(Web Server issue):W29-37. doi:10.1093/nar/gkr367.

8. Wallace IM, O'Sullivan O, Higgins DG and Notredame C. M-Coffee: combining multiple sequence alignment methods with T-Coffee. Nucleic Acids Res. 2006;34 (6):1692-9. doi:10.1093/nar/gkl091.
9. Trapnell C, Hendrickson DG, Sauvageau M, Goff L, Rinn JL and Pachter L. Differential analysis of gene regulation at transcript resolution with RNA-seq. Nat Biotechnol. 2013;31(1):46-53. doi:10.1038/nbt.2450.
10. Anders S and Huber W. Differential expression analysis for sequence count data. Genome Biol. 2010;11(10):R106. doi:10.1186/gb-2010-11-10-r106.
11. Yamaji M, Jishage M, Meyer C, Suryawanshi H, Der E, Yamaji M, et al. DND1 maintains germline stem cells via recruitment of the CCR4-NOT complex to target mRNAs. Nature. 2017;543(7646):568-72. doi:10.1038/nature21690.
12. Zhang Y, Sloan SA, Clarke LE, Caneda C, Plaza CA, Blumenthal PD, et al. Purification and characterization of progenitor and mature human astrocytes reveals transcriptional and functional differences with mouse. Neuron. 2016;89 (1):37-53. doi:10.1016/j.neuron.2015.11.013.
13. Andor N, Graham TA, Jansen M, Xia LC, Aktipis CA, Petritsch C, et al. Pan-cancer analysis of the extent and consequences of intratumor heterogeneity. Nat Med. 2016;22(1):105-13. doi:10.1038/nm.3984.
14. Treutlein B, Lee QY, Camp JG, Mall M, Koh W, Shariati SA, et al. Dissecting direct reprogramming from fibroblast to neuron using single-cell RNA-seq. Nature. 2016;534(7607):391-5. doi:10.1038/nature18323.
15. Okin D and Medzhitov R. The effect of sustained inflammation on hepatic mevalonate pathway results in hyperglycemia. Cell. 2016;165(2):343-56. doi:10.1016/j.cell.2016.02.023.
16. Pimentel H, Parra M, Gee SL, Mohandas N, Pachter L and Conboy JG. A dynamic intron retention program enriched in RNA processing genes regulates gene expression during terminal erythropoiesis. Nucleic Acids Res. 2016;44(2):838-51. doi:10.1093/nar/gkv1168.
17. Mahat DB, Salamanca HH, Duarte FM, Danko CG and Lis JT. Mammalian heat shock response and mechanisms underlying its genome-wide transcriptional regulation. Mol Cell. 2016;62(1):63-78. doi:10.1016/j.molcel.2016.02.025.
18. Ortiz-Ramirez C, Hernandez-Coronado M, Thamm A, Catarino B, Wang M, Dolan L, et al. A transcriptome atlas of physcomitrella patens provides insights into the evolution and development of land plants. Mol Plant. 2016;9(2):205-20. doi:10.1016/j.molp.2015.12.002.
19. Lacar B, Linker SB, Jaeger BN, Krishnaswami S, Barron J, Kelder M, et al. Nuclear RNA-seq of single neurons reveals molecular signatures of activation. Nat Commun. 2016;7:11022. doi:10.1038/ncomms11022.
20. Timmons JA, Szkop KJ and Gallagher IJ. Multiple sources of bias confound functional enrichment analysis of global -omics data. Genome Biol. 2015;16:186. doi:10.1186/s13059-015-0761-7.
21. Schmieder R and Edwards R. Quality control and preprocessing of metagenomic datasets. Bioinformatics. 2011;27(6):863-4. doi:10.1093/bioinformatics/btr026.
22. Martin M. Cutadapt removes adapter sequences from high-throughput sequencing reads. EMBnetJournal. 2011;17 1:10-2.
23. Qiu Q, Wang L, Wang K, Yang Y, Ma T, Wang Z, et al. Yak whole-genome resequencing reveals domestication signatures and prehistoric population expansions. Nat Commun. 2015;6:10283. doi:10.1038/ncomms10283.
24. Daetwyler HD, Capitan A, Pausch H, Stothard P, van Binsbergen R, Brondum RF, et al. Whole-genome sequencing of 234 bulls facilitates mapping of monogenic and complex traits in

cattle. Nat Genet. 2014;46(8):858-65. doi:10.1038/ng.3034.
25. Ai H, Fang X, Yang B, Huang Z, Chen H, Mao L, et al. Adaptation and possible ancient interspecies introgression in pigs identified by whole-genome sequencing. Nat Genet. 2015;47(3):217-25. doi:10.1038/ng.3199.
26. Li D, Che T, Chen B, Tian S, Zhou X, Zhang G, et al. Genomic data for 78 chickens from 14 populations. GigaScience. 2017;6(6):1-5. doi:10.1093/gigascience/gix026.
