# Supplementary material for: Comparative transcriptomics of 5 high-altitude vertebrates and their low-altitude relatives
Source: Gigascience. 2017 Nov 15;6(12):1–9. doi: 10.1093/gigascience/gix105 (PMC5729692; doi:10.1093/gigascience/gix105)
Supplement: Response-to-Reviewer-Comments_Revision-1.pdf [file gix105_response-to-reviewer-comments_revision-1.pdf]

Author's Response To Reviewer Comments

Reviewer 1:

Comment 2-1
Your Series GSE93855 submission is labeled as unavailable until Jan 13 2020. Under the FAIR principles of publication in GigaScience, this must change if paper is accepted. Other datasets are also embargoed, far into 2018 or 2019.
Response 2-1
Thank you for your reminder. We have released all of datasets related to our manuscript, inducing a total of 180 RNA-seq data deposited in the NCBI Gene Expression Omnibus (GEO) under accession numbers GSE93855, GSE77020 and GSE66242, as well as the 7 whole-genome sequencing data deposited in the NCBI-sequence read archive (SRA) under accession number SRP096151.

GSE93855: https://www.ncbi.nlm.nih.gov/geo/query/acc.cgi?acc=GSE93855
GSE77020: https://www.ncbi.nlm.nih.gov/geo/query/acc.cgi?acc=GSE77020
GSE66242: https://www.ncbi.nlm.nih.gov/geo/query/acc.cgi?acc=GSE66242
SRP096151: https://trace.ncbi.nlm.nih.gov/Traces/sra/?study=SRP096151

Comment 2-2
The additional work performed to answer my reviewer comments is very good and shows interesting results. I don't have further major concerns, other than fixing the data availability issue above.
Response 2-2
Thank you for your positive comments.

Comment 2-3
The authors could consider revising Figure 4b and 4d, which are quite complex and difficult to see the relationships of all datasets to each other. The use of different color lines was not explained, and I tried to see why these were used, but was unable to see the logic.
Response 2-3
Thank you for your valuable suggestion. In Fig. 4b and 4d, the vertical leading lines with different colors from the plotted points dropping to the xy-plane serve to improve the demonstration of group separation by tissue based on the first and second principal components. We added the explanation of the dropping vertical lines to the legends of Fig. 4b and 4d, "The vertical leading lines with different colors from the plotted points dropping to the xy-plane show the separation of points based on the first and second principal components".
To further promote the clarity of depiction for the principal component analysis (PCA) results, we added the two-dimensional PCA figures as the Supplementary Figs. S14-15 (accessible from Response_sup.pdf at: https://www.dropbox.com/s/sivc1djpvqzty2s/Response_sup.pdf?dl=0)

Reviewer 2:

Comment 3-1
Thank you for addressing the previous points raised during the first reviewing stage. I believe

that most points have been addressed in an appropriate way, and once again I thank you for your work and the data generated.

Response 3-1

Thank you for your positive comments.
