# Supplementary material for: Comparative transcriptomics of 5 high-altitude vertebrates and their low-altitude relatives
Source: Gigascience. 2017 Nov 15;6(12):1–9. doi: 10.1093/gigascience/gix105 (PMC5729692; doi:10.1093/gigascience/gix105)
Supplement: Reviewer-1-Report-(Original-Submission).pdf [file gix105_reviewer-1-report-(original-submission).pdf]

Reviewer Report

Title: Comparative transcriptomics of five high-altitude vertebrates and their low-altitude relatives

Version: Original Submission **Date:** 4/7/2017

Reviewer name: Christopher Tuggle

Reviewer Comments to Author:

The authors report a well-developed project to better understand the gene expression differences in multiple tissues from 5 species (with the cattle-yak comparison counted as one). The data collected is enormous and clearly appears to be sufficient for the analyses proposed, but there are a number of questions regarding both the methods and results presented.Of highest importance, the authors present a set of analyses in which the output is a list of genes and a calculated expression level; these lists are then used in a number of ways to calculate expression and enriched function per tissue in several comparison. These lists (and not even the numbers of genes in each list) are not provided, so it is impossible to see these lists or use the lists as a resource for other work. Since one aspect of this publication would be as a resource for others, the authors must provide these lists as well as the calculated expression value for each gene. I realize these lists are extensive, but are a crucial component of the resource, especially for those readers who will not start with the raw data, but also for those who can repeat the analyses and compare their resulting normalized expression data with those that the authors created.Further, the authors describe some biological results on comparisons between high and low altitude, but fail to provide sufficient description of the results. The Supplementary file is incomplete (see below), but also the text on all tissue and species comparisons is only a few sentences. More is needed to justify this reporting. For example, a strength of the work is the multi-species comparison of the same question of adaptation to high altitude. A comparison of the high/low differentially expressed gene lists in the same tissue across species would seem minimal and potentially very interesting- i.e., are the genes and pathways identified similar (more similar than expectation?). This would provide more insight, as well as more evidence the analyses are providing biologically relevant information.1. Criterion for expression.a) On line 40, the authors indicate they are using a FPKM of 0.1. I was unable to find specific details on the sequencing data so that I could determine the number of counts this represents. I could not find the read length nor whether this was SE or PE. Assuming 100 nt read length and PE for the average of 5 Gb for each tissue reported, a FPKM of 0.1 is 2.5 counts for a 1 kb transcript. This is very low. The authors should justify this low cutoff, which affects all subsequent analyses. I would like to see the median expression level for each tissue, as well.b) On line 188, the authors use the term "high confidence single-copy orthologs" this is not defined. And is this homology based or expression based?2. Comparison of expression differences between high and low altitude animals and functional annotation analysis.a) Supplemental Figure S3 shows that in some tissues there are large differences in mapping rate that are not reflected in the other altitude type. Did the authors check that mapping rate did not affect their differential expression calls? Also, please report the tissue type in this graph.b) In Additional File 2, a large table provided the GO/KEGG/InterPro terms and whether lists of genes with specific difference in high/low altitude expression are significantly enriched for that term. The authors should

show the number of genes in the list for each comparison, or only show those with at least 5-10 genes in a list. Low representation in a pathway or term can be misleading for enrichment.c) More importantly, the authors do not indicate the background used for these analyses. It would be most appropriate to use the total number of genes expressed in each tissue for such analyses, so that the background reflects the genes that could possibly be shown to be differentially expressed, not the genome-wide background which is often the default.

Level of Interest

Please indicate how interesting you found the manuscript: An article of importance in its field

Quality of Written English

Please indicate the quality of language in the manuscript: Acceptable

Declaration of Competing Interests

Please complete a declaration of competing interests, considering the following questions:

- Have you in the past five years received reimbursements, fees, funding, or salary from an organisation that may in any way gain or lose financially from the publication of this manuscript, either now or in the future?
- Do you hold any stocks or shares in an organisation that may in any way gain or lose financially from the publication of this manuscript, either now or in the future?
- Do you hold or are you currently applying for any patents relating to the content of the manuscript?
- Have you received reimbursements, fees, funding, or salary from an organization that holds or has applied for patents relating to the content of the manuscript?
- Do you have any other financial competing interests?
- Do you have any non-financial competing interests in relation to this paper?

If you can answer no to all of the above, write 'I declare that I have no competing interests' below. If your reply is yes to any, please give details below.

I declare that I have no competing interests

I agree to the open peer review policy of the journal. I understand that my name will be included on my report to the authors and, if the manuscript is accepted for publication, my named report including any attachments I upload will be posted on the website along with the authors' responses. I agree for my report to be made available under an Open Access Creative Commons CC-BY license (http://creativecommons.org/licenses/by/4.0/). I understand that any comments which I do not wish to

be included in my named report can be included as confidential comments to the editors, which will not be published.

I agree to the open peer review policy of the journal
