# Supplementary material for: Comparative transcriptomics of 5 high-altitude vertebrates and their low-altitude relatives
Source: Gigascience. 2017 Nov 15;6(12):1–9. doi: 10.1093/gigascience/gix105 (PMC5729692; doi:10.1093/gigascience/gix105)
Supplement: Reviewer-2-Report-(Original-Submission).pdf [file gix105_reviewer-2-report-(original-submission).pdf]

Reviewer Report

Title: Comparative transcriptomics of five high-altitude vertebrates and their low-altitude relatives

Version: Original Submission **Date:** 5/2/2017

Reviewer name: Andreia Amaral

Reviewer Comments to Author:

Dear Dr Li,First of all let me congratulate you and all authors for this piece of research. I have although some questions that I believe are important in order to improve your manuscript:In Data Analysis:page 4, lines 85-88: may you specify how the data filtering was performed? which software did you use, or in case you have used in house developed scripts may you please provide them as supplemental information?page 4 line 93, may you specify the parameters used for the analysis performed with EnsemblComparaGeneTrees method?page 4- line 96, may you please detail the parameters used for the BWA alignment?page 5- line 101- which were the parameters defined for GATK detection of SNPs and Indels? Parameters like Calling confidence and minimum read depth?page 5 line 108- which parameters were used for the TopHat alignment?In Findings:I am missing analysis that I was expecting in a study of adaptation to altitude which generated so much WGS data. I suggest that you study genetic divergence by Fst or by Tajima's D and make identification of selection footprints. It would be great then to compare the genes being harbored in selective sweeps and the changes at transcriptomic level.page 10 lines 230-235: Did this happen in the low altitude chicken or only in one? its hard to see this in the figurepage 11 lines 251-259: The way these results are presented its hard to infer if the pathways affected by adaptation to altitude if these were the same between species or not. This is an important question that your results would enable to answer. I would suggest that a table per species should be made as well as venn diagrams that would lead us to understand which pathways were commonly affected or were different between species and if these were the same also at tissue level. I would like to see this part of the manuscript more enhanced, giving a larger value to the high value data that you have generated in your research.With kind regards,Andreia J. Amaral

Level of Interest

Please indicate how interesting you found the manuscript: An article of importance in its field

Quality of Written English

Please indicate the quality of language in the manuscript: Acceptable

Declaration of Competing Interests

Please complete a declaration of competing interests, considering the following questions:

- Have you in the past five years received reimbursements, fees, funding, or salary from an organisation that may in any way gain or lose financially from the publication of this manuscript, either now or in the future?
- Do you hold any stocks or shares in an organisation that may in any way gain or lose financially from the publication of this manuscript, either now or in the future?
- Do you hold or are you currently applying for any patents relating to the content of the manuscript?
- Have you received reimbursements, fees, funding, or salary from an organization that holds or has applied for patents relating to the content of the manuscript?
- Do you have any other financial competing interests?
- Do you have any non-financial competing interests in relation to this paper?

If you can answer no to all of the above, write 'I declare that I have no competing interests' below. If your reply is yes to any, please give details below.

I declare that I have no competing interests

I agree to the open peer review policy of the journal. I understand that my name will be included on my report to the authors and, if the manuscript is accepted for publication, my named report including any attachments I upload will be posted on the website along with the authors' responses. I agree for my report to be made available under an Open Access Creative Commons CC-BY license (http://creativecommons.org/licenses/by/4.0/). I understand that any comments which I do not wish to be included in my named report can be included as confidential comments to the editors, which will not be published.

I agree to the open peer review policy of the journal
